# Using real-world data for supporting regulatory decision making: Comparison of cardiovascular and safety outcomes of an empagliflozin randomized clinical trial versus real-world data

**DOI:** 10.3389/fphar.2022.928121

**Published:** 2022-08-30

**Authors:** Ha Young Jang, In-Wha Kim, Jung Mi Oh

**Affiliations:** College of Pharmacy and Research Institute of Pharmaceutical Sciences, Seoul National University, Seoul, South Korea

**Keywords:** real-world evidence, randomized controlled trial, emulation analysis, diabetes mellitus, sitagliptin, empagliflozin

## Abstract

**Aims:** In countries where a randomized clinical trial (RCT) is difficult to perform, a real-world evidence (RWE) study with a design similar to an RCT may be an option for drug regulatory decision-making. In this study, the objective was to find out to what extent the safety of empagliflozin from the RWE study in Korea is different from the one in RCT by emulating the design of foreign RCT. The outcome covers various safety outcomes including cardiovascular safety.

**Methods:** The EMPA-REG OUTCOME trial (NCT01131676) was selected for comparison. The inclusion/exclusion criteria and follow-up method for the RWE were matched to the comparison RCT. Major adverse cardiovascular events (MACEs) were used as a primary outcome and 15 other outcomes were also included for analysis.

**Result:** We followed 23,126 matched patients with type 2 diabetes mellitus (11,563 empagliflozin users and 11,563 sitagliptin users) for 2.7 years (median). Empagliflozin use was associated with a significantly decreased risk of MACEs [EMPA-REG DUPLICATE RWE: adjusted HR 0.87, 95% confidence interval (CI) 0.79–0.96]. The predefined estimate agreement, regulatory agreement, and standardized difference for RCT duplication were achieved [EMPA-REG OUTCOME RCT: adjusted HR 0.86, 95% (CI) 0.74–0.99]. According to the predefined criteria for 15 outcomes, 10 outcomes were evaluated as good, and three as moderate.

**Conclusion:** Our study results suggest that RWE in one country in comparison with an RCT has the potential for providing evidence for future regulatory decision-making in an environment where RCT could not be performed.

## Introduction

Randomized controlled trials (RCTs) are generally regarded as the gold standard for regulatory decision makings. Given the growing trend of globalization and the need to make new or extended-use medicines rapidly available to patients worldwide, the RCTs are usually conducted in multi-regional clinical settings (Quan et al., 2017). However, since most clinical trials are conducted in the US and Europe, the proportion of Asians is relatively low. It has been reported that the proportion of clinical trials in Korea among the total clinical trial is about 3% (ClinicalTrials.gov, 2022). Data from multi-regional clinical trials (MRCTs) are submitted to regulatory agencies, which currently find it difficult to evaluate such data for drug approval (Sohn et al., 2019). The main reason is that clinical trial subjects are of different races. Furthermore, it is difficult to conduct additional clinical trials for regulatory decisions like expanding drug indications or adding side effects information, due to time and cost (Revicki and Frank, 1999; Garrison et al., 2007).

Real-world evidence (RWE) is clinical evidence concerning the potential benefits or risks of a medication derived from analysis of real-world data (RWD). RWE has a relative advantage over RCTs because it enables a long-term follow-up study or research on rare populations. In the United States, the 21st Century Cures Act, passed in 2016, placed additional focus on the use of RWE to support regulatory decision making, including adding/modifying an indication, use in a new population, and adding comparative effectiveness or safety information (Food-and-Drug-Administration-FDA, 2018a; Food-and-Drug-Administration-FDA, 2018b; Food-and-Drug-Administration-FDA, 2019a; Food-and-Drug-Administration-FDA, 2019b). With a rise in observational COVID-19 study dissemination, this trend is being accelerated (Pundi et al., 2020). Rather than performing additional RCT in every country to verify new indications or side effects, performing an RWE study in other races and medical-practice conditions could be an alternative way. If the design and analysis method of the RWE study are implemented as closely as possible with the RCT, it will be easier to make regulatory decisions based on comparisons of results of RWEs and RCTs.

Empagliflozin is a sodium-glucose cotransporter 2 (SGLT2) inhibitor drug approved by US Food and Drug Administration (FDA) in 2014 for the treatment of type 2 diabetes (T2DM). After its approval for T2DM, several RCTs have been performed to demonstrate the safety of empagliflozin for other outcomes (Zinman et al., 2015; Packer et al., 2020). New indications such as reducing the risk of cardiovascular death in adults with T2DM and established cardiovascular disease and hospitalization for heart failure in adults with heart failure were added under FDA approval (Dailymed-Prescribing-information, 2022). However, the Ministry of Food and Drug Safety (MFDS) in Korea has not yet recognized the safety of empagliflozin for cardiovascular disease. This is because sufficient evidence has not been provided for whether the indication, “reducing the risk of cardiovascular death” could be demonstrated for Koreans as well. For this reason, empagliflozin has not yet been approved for reducing the risk of cardiovascular disease (MFDS-Prescribing-information, 2021).

In this study, we aimed to investigate to what extent the safety of empagliflozin from the RWE study in Korea is different from the one in RCT by emulating the design of foreign RCT. The outcome covers various safety outcomes including cardiovascular safety. We applied a RCT emulation analysis process that would be acceptable for regulation (Franklin and Schneeweiss, 2017; Franklin et al., 2020). If there were any discrepancies between the RCT and RWE, we investigated the circumstances under which this inconsistency occurs.

## Methods

### Study design and data sources

The study drug was selected through a pre-determined process (Supplementary Figure S1). Firstly, drugs that need to be re-evaluated under MFDS (date of announcement: 2021-01-24) were assessed (number of drugs: 498) (Supplementary Table S1). Secondly, according to the selection criteria set by the research team, 91 drugs were considered having high demand for safety evaluation. Of those, the anti-diabetic medications consisting largest number of drugs (number of drugs: 7) were selected (Supplementary Table S2) An additional selection process was carried out with considering each drugs’ adverse reaction profiles. Finally, empagliflozin and its pivotal study (EMPA-REG Outcome) were selected as a target drug and a target trial, respectively. This 1:1 matched cohort study included patients with type 2 diabetes mellitus and high cardiovascular risk, using the same inclusion/exclusion criteria, follow-up method and outcome definitions of a target RCT. The study assessed the effect of empagliflozin versus sitagliptin on cardiovascular and several safety outcomes of empagliflozin. The EMPA-REG OUTCOME trial (NCT01131676) (Zinman et al., 2015) was selected to target emulation (Franklin et al., 2020; Franklin et al., 2021). The EMPA-REG OUTCOME study provided strong evidence that the SGLT2 inhibitor empagliflozin protects against major adverse cardiovascular events (MACEs) and other secondary outcomes (Zinman et al., 2015).

The analyzed health insurance data was officially provided by the Korean Health Insurance Review & Assessment Service (HIRA) (Kim et al., 2017). The insurance data included demographic, diagnosis, procedure, and prescription data of patients. The requirement for written informed consent from participants was waived because all participants were anonymized using a randomized identification number. This study was approved by the institutional review board of Seoul National University (IRB No. E2101/001-003). This study followed the Strengthening the Reporting of Observational Studies in Epidemiology (STROBE) guideline (von Elm et al., 2014).

### Study patients

The target population is patients with T2DM and established cardiovascular disease. Patients who had been diagnosed with T2DM were included from 2011 to 2020, with a 3 years of study index period between May 2016 and May 2018. The period between January 2011 to May 2016 was used as a screening period for applying inclusion/exclusion criteria. Patients were selected according to the same inclusion/exclusion criteria as a RCT (Supplementary Table S3). All patients (≥18 years) had established cardiovascular disease and received empagliflozin or sitagliptin for the first time. Note that according to 2013 American College of Cardiology and American Heart Association guideline, patients who have been diagnosed with an established cardiovascular disease are classified as a high-risk group (Karmali et al., 2014). Therefore, included patients were considered as having high cardiovascular disease risks. We selected an active comparator (sitagliptin) as a proxy for the placebo, because it is well known for observational studies, that a non-user comparator group can differ substantially from actively treated patients, unlike RCTs (Food-and-Drug-Administration-FDA, 2013). Many other studies have also selected Dipeptidyl peptidase-4 inhibitors as comparators for assessment of SGLT-2 safety (Kim et al., 2018; Douros et al., 2020; Lee et al., 2020; Seong et al., 2020; Han et al., 2021). The index date was defined as the very first date each drug was prescribed.

### Key variables

Individuals were followed-up until May 2020, and outcomes were recorded between each individual’s index date and May 2020. MACEs outcome from the EMPA-REG OUTCOME trial was used as a primary outcome. Since HIRA does not provide cause of death information, modified MACEs (all-cause death, myocardial infarction (MI), and stroke) was applied (Yeom et al., 2015). A total of seven cardiovascular outcomes were analyzed: all-cause death, MI, hospitalization for unstable angina, coronary revascularization procedure, stroke, transient ischemic attack, and hospitalization for heart failure.

Eight safety outcomes were also analyzed: hypoglycemic events, urinary tract infections (UTIs), genital infections, volume depletion, acute kidney injury (AKI), diabetic ketoacidosis (DKA), thromboembolic events, and bone fracture. The operational definitions of outcomes were defined using the Korean Standard Classification of Diseases-9 codes or procedure codes and were directly matched to each Regulatory Activities Preferred Term (MedDRA PT) in the RCT (Supplementary Table S4). To minimize confounding variables (e.g., selection bias) as much as possible, 72 covariates were included viz. Demographics, comorbidities, and disease/outcome specific variables. Of those, the main variables included are as follows: Seven types of glucose-lowering therapies (Metformin, Insulins, Sulfonylureas, Glitazones, Glucagon-like peptide-1 agonists, Alpha-glucosidase inhibitors, and Meglitinides) [Diabetes treatment strategies], time since type 2 diabetes mellitus [Duration of continuous enrolment], number of inpatient/outpatient visit [Indicators of health care utilization of the patients], five types of cardiovascular risk factors (Coronary artery disease (CAD), Multi vessel CAD, MI, Coronary Artery Bypass Graft, and Stroke with proper cardiovascular procedures) [history of cardiovascular procedures]. All covariates within the preceding 1 year of index date were evaluated.

### Statistical analysis

Statistical analyses were performed for the intention-to-treat population. Each time an outcome was analyzed, a new cohort was constructed after excluding patients with a history of the corresponding outcome. Patients were followed up until the earliest of events, the date of last follow-up, the date of switching diabetic medication to the other comparison group, or the end of the study period. The maximum follow-up period was set at 48 months (same as in the RCT). Empagliflozin users were matched 1:1 to sitagliptin users and the distribution of the propensity score was inspected (Parsons, 2001). A standardized difference >0.1 was regarded as a sign of imbalance (NCSS-statistical-Software, 2017). As same with RCT, the age and sex-adjusted multivariate Cox proportional hazard regression was used to estimate the hazard ratio (HR) of empagliflozin for the cardiovascular outcome, with a 95% confidence interval (CI). For a safety outcome model, logistic regression was used to the odds ratio (OR) of empagliflozin.

Sensitivity analyses were performed the same as with the RCTs in two ways. First, patients who received at least one dose of the study drug were observed until ≤30 days after a patient’s last intake of medication. Additionally, we followed up patients who received the study drug for ≥30 days (cumulative) including events that only occurred ≤30 days after a patient’s last intake of medication (“as-treated” analysis). Analyses were performed with SAS Enterprise Guide version 7.1 (SAS Institute Inc., Cary, NC, United States).

### RCT-RWE agreement assessment

We defined three metrics below to make a binary decision on whether an RCT was successfully emulated, considering statistical significance, directionality, and CIs associated with the corresponding RWE study. The agreement criteria suggested by Franklin *et al.* were used for determining each agreement (Franklin et al., 2020). First, we defined regulatory agreement (RA) as the ability of the RWE study to emulate the direction and statistical significance of the randomized trial finding. A secondary agreement metric was the estimate agreement (EA), defined as an RWE estimate that lies within the RCT 95% CI. We also conducted hypothesis tests to evaluate whether there was a difference in findings by calculating the standardized difference (SD) between the RCT and RWE effect estimates. We considered a *p*-value < 0.05 (where SD is greater than 1.96) statistically significant for the SD agreement. For comparison of results, HRs for cardiovascular outcomes and ORs for safety outcomes were calculated and compared (HRs were not provided for safety outcomes in an RCT). We defined the emulation result as “good” or “moderate” if all three agreements or two of three agreements were achieved, respectively. If the emulation result achieved ≤ one of the agreements, we defined the result as ‘fail’.

## Results

**FIGURE 1:**
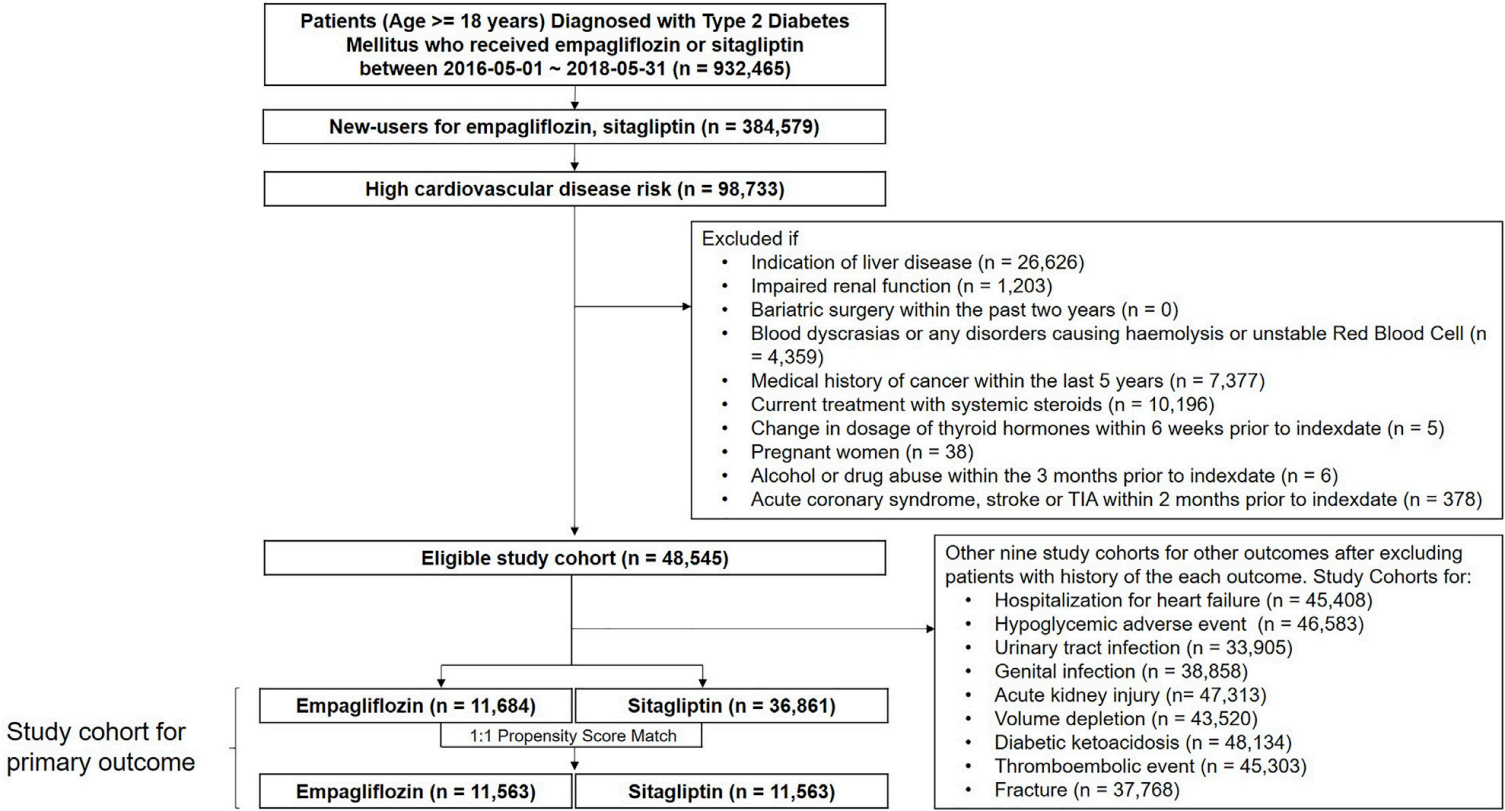
Study flow chart.

A total of 932,465 patients (age ≥18 years) diagnosed with diabetes who received empagliflozin or sitagliptin were identified. New empagliflozin or sitagliptin users (n = 384,579) were selected (Figure 1). Among 98,733 patients who have high cardiovascular disease risks, an eligible study cohort with 48,545 patients remained after excluding patients who do not meet predefined inclusion criteria. Sitagliptin users were older and visited clinics more frequently (inpatient/outpatient) than empagliflozin users (Table 1). A later index date of empagliflozin users was observed compared to sitagliptin users. Compared to sitagliptin users, empagliflozin users were more often diagnosed with coronary artery disease (including coronary revascularization) and had fewer strokes.

**TABLE 1:** Baseline characteristics.

Variables	Pre-Match			Post-Match			
	**Sitagliptin N = 36,861**	**EmpagliflozinN = 11,684**	**STD**	**Sitagliptin N = 11,563**	**Empagliflozin N = 11,563**		**STD**
Sex	20,289 (55)	6,913 (59.2)	−0.04	6,799 (58.8)	6,829 (59.1)		−0.004
Age	60.4 ± 11.4	55.4 ± 11	−0.4	55.5 ± 11.3	55.6 ± 10.9		0.008
Insurance type							
Normal	34,434 (93.4)	11,065 (94.7)	0.06	10,927 (94.5)	10,950 (94.7)		0.01
Medicaid	2,234 (6.1)	583 (5)		595 (5.2)	577 (5)		
No charge	193 (0.5)	36 (0.3)		41 (0.4)	36 (0.3)		
Number of Inpatient visit	0.8 ± 2.2	0.5 ± 1.2	−0.2	0.5 ± 1.1	0.5 ± 1.2		0.01
Number of outpatient visit	28.1 ± 27.5	25.5 ± 23.9	−0.1	25.3 ± 23.1	25.6 ± 23.9		0.01
Time since type 2 diabetes mellitus							
≤1 year	18,197 (49.4)	5,045 (43.2)	0.1	5,043 (43.6)	5,008 (43.3)		0.007
>1–5 years	16,927 (45.9)	5,941 (50.9)		5,827 (50.4)	5,866 (50.7)		
>5 years	1737 (4.7)	698 (6)		693 (6)	689 (6)		
Index year							
2016	10,568 (28.7)	2,247 (19.2)	0.2	2,281 (19.7)	2,247 (19.4)		0.01
2017	18,283 (49.6)	6,336 (54.2)		6,286 (54.4)	6,270 (54.2)		
2018	8,010 (21.7)	3,101 (26.5)		2,996 (25.9)	3,046 (26.3)		
Charlson comorbidity index							
0	1,521 (4.1)	473 (4.1)	0.03	469 (4.1)	468 (4.1)		0.004
1	3,361 (9.1)	1,144 (9.8)		1,150 (10)	1,138 (9.8)		
2	4,937 (13.4)	1,644 (14.1)		1,634 (14.1)	1,629 (14.1)		
3	27,042 (73.4)	8,423 (72.1)		8,310 (71.9)	8,328 (72)		
CV risk factor							
CAD	32,597 (88.4)	10,817 (92.6)	0.1	10,657 (92.2)	10,699 (92.5)		0.01
Multi vessel CAD	16,230 (44)	6,161 (52.7)	0.2	5,944 (51.4)	6,056 (52.4)		0.02
MI	1920 (5.2)	911 (7.8)	0.1	836 (7.2)	877 (7.6)		0.01
CABG	7,665 (20.8)	3,341 (28.6)	0.2	3,195 (27.6)	3,264 (28.2)		0.01
Stroke	5,299 (14.4)	1,062 (9.1)	−0.2	1,030 (8.9)	1,057 (9.1)		0.008
PAD	2,376 (6.5)	666 (5.7)	−0.03	687 (5.9)	661 (5.7)		−0.010
DM circulation	4,921 (13.4)	1802 (15.4)	0.06	1760 (15.2)	1775 (15.4)		0.004
DM foot	3 (0.0)	4 (0.0)	0.02	3 (0.0)	1 (0.0)		−0.01
DM nephropathy	2,365 (6.4)	1,009 (8.6)	0.08	965 (8.4)	985 (8.5)		0.006
DM neuropathy	5,274 (14.3)	1,591 (13.6)	−0.02	1,620 (14)	1,574 (13.6)		−0.01
DM other Complications	27,326 (74.1)	8,353 (71.5)	−0.06	8,288 (71.7)	8,287 (71.7)		0.000
Hyperglycemia	694 (1.9)	159 (1.4)	−0.04	160 (1.4)	154 (1.3)		−0.005
Comorbidities							
Hypertension	28,872 (78.3)	9,208 (78.8)	0.01	9,095 (78.7)	9,108 (78.8)		0.003
Edema	3,490 (9.5)	1,066 (9.1)	−0.01	1,065 (9.2)	1,056 (9.1)		−0.003
Kidney stone	585 (1.6)	168 (1.4)	−0.01	189 (1.6)	167 (1.4)		−0.02
Osteoarthritis	13,169 (35.7)	3,580 (30.6)	−0.1	3,561 (30.8)	3,568 (30.9)		0.001
Other arthritis	9,104 (24.7)	2,570 (22)	−0.06	2,484 (21.5)	2,554 (22.1)		0.02
PUD	9,380 (25.5)	2,828 (24.2)	−0.03	2,733 (23.6)	2,796 (24.2)		0.01
Pancreatitis	342 (0.9)	103 (0.9)	0.00	105 (0.9)	102 (0.9)		−0.003
UC	59 (0.2)	12 (0.1)	−0.02	10 (0.1)	12 (0.1)		0.006
Crohn	15 (0.0)	5 (0.0)	0.00	5 (0.0)	5 (0.0)		0.000
Asthma	5,421 (14.7)	1,626 (13.9)	−0.02	1,596 (13.8)	1,602 (13.9)		0.002
COPD	1,349 (3.7)	296 (2.5)	−0.07	300 (2.6)	295 (2.6)		−0.003
Bladder stone	29 (0.1)	5 (0.0)	−0.01	5 (0.0)	5 (0.0)		0.000
Dementia	5,993 (16.3)	1,153 (9.9)	−0.2	1,141 (9.9)	1,150 (10)		0.003
Electrolyte Imbalance	2,353 (6.4)	608 (5.2)	−0.05	585 (5.1)	600 (5.2)		0.006
Glaucoma/Cataract	10,509 (28.5)	3,176 (27.2)	−0.03	3,127 (27)	3,152 (27.3)		0.005
HONK	285 (0.8)	64 (0.6)	−0.03	65 (0.6)	63 (0.5)		−0.002
HTN nephropathy	166 (0.5)	54 (0.5)	0.00	40 (0.4)	51 (0.4)		0.02
Hyperthyroid disease	704 (1.9)	225 (1.9)	0.00	226 (2)	224 (1.9)		−0.001
Hypothyroid disease	1802 (4.9)	602 (5.2)	0.01	597 (5.2)	594 (5.1)		−0.001
Osteomyelitis	282 (0.8)	66 (0.6)	−0.02	56 (0.5)	66 (0.6)		0.01
Pneumonia	2,872 (7.8)	770 (6.6)	−0.05	749 (6.5)	763 (6.6)		0.005
Skin infection	1,438 (3.9)	459 (3.9)	0.00	459 (4)	455 (3.9)		−0.002
Glucose-lowering therapy							
Metformin	25,836 (70.1)	8,466 (72.5)	0.05	8,422 (72.8)	8,382 (72.5)		−0.008
Insulins	6,312 (17.1)	2,118 (18.1)	0.03	2098 (18.1)	2074 (17.9)		−0.005
SUs	16,898 (45.8)	5,499 (47.1)	0.02	5,428 (46.9)	5,441 (47.1)		0.002
Glitazones	3,280 (8.9)	1,328 (11.4)	0.08	1,309 (11.3)	1,301 (11.3)		−0.002
GLP-1 agonists	112 (0.3)	81 (0.7)	0.06	78 (0.7)	74 (0.6)		−0.004
AGIs	1,532 (4.2)	364 (3.1)	−0.06	366 (3.2)	362 (3.1)		−0.002
Meglitinides	253 (0.7)	85 (0.7)	0.00	83 (0.7)	82 (0.7)		−0.001
Co-medications							
Anticoagulants	1,650 (4.5)	564 (4.8)	0.02	523 (4.5)	550 (4.8)		0.01
Antiplatelets	24,499 (66.5)	8,232 (70.5)	0.09	8,111 (70.2)	8,141 (70.4)		0.006
Heparins	1,287 (3.5)	354 (3)	−0.03	338 (2.9)	352 (3)		0.007
Thrombolytics	58 (0.2)	10 (0.1)	−0.02	7 (0.1)	10 (0.1)		0.01
Statins	25,978 (70.5)	9,459 (81)	0.3	9,287 (80.3)	9,343 (80.8)		0.01
Other lipid Lowerings	3,903 (10.6)	1,678 (14.4)	0.1	1,627 (14.1)	1,633 (14.1)		0.002
Nitrates	6,264 (17)	2,441 (20.9)	0.1	2,378 (20.6)	2,393 (20.7)		0.003
Digoxin	5,390 (14.6)	2,134 (18.3)	0.1	2060 (17.8)	2087 (18.1)		0.006
ACEIs	2,127 (5.8)	963 (8.2)	0.1	929 (8)	927 (8)		−0.001
ARBs	21,506 (58.3)	7,292 (62.4)	0.08	7,171 (62)	7,198 (62.3)		0.005
Entresto	6 (0)	17 (0.2)	0.05	6 (0.1)	8 (0.1)		0.007
Other Anti HTNs	24,131 (65.5)	8,132 (69.6)	0.09	8,017 (69.3)	8,025 (69.4)		0.002
Loop diuretics	4,310 (11.7)	1,364 (11.7)	0.00	1,292 (11.2)	1,327 (11.5)		0.01
Other diuretics	10,016 (27.2)	3,223 (27.6)	0.01	3,076 (26.6)	3,165 (27.4)		0.02
Antianxieties	14,982 (40.6)	4,215 (36.1)	−0.09	4,133 (35.7)	4,183 (36.2)		0.009
Antipsychotics	1800 (4.9)	302 (2.6)	−0.1	299 (2.6)	301 (2.6)		0.001
Antidepressants	6,667 (18.1)	1771 (15.2)	−0.08	1777 (15.4)	1759 (15.2)		−0.004
Dementia	5,993 (16.3)	1,153 (9.9)	−0.2	1,141 (9.9)	1,150 (10)		0.003
Antiparkinsons	1,139 (3.1)	179 (1.5)	−0.1	164 (1.4)	179 (1.6)		0.01
Anticonvulsants	934 (2.5)	186 (1.6)	−0.07	200 (1.7)	186 (1.6)		−0.01
NSAIDs	28,032 (76.1)	8,810 (75.4)	−0.02	8,757 (75.7)	8,733 (75.5)		−0.005
Bisphos-phonates	1765 (4.8)	373 (3.2)	−0.08	379 (3.3)	371 (3.2)		−0.004
Opioids	16,376 (44.4)	4,778 (40.9)	−0.07	4,720 (40.8)	4,732 (40.9)		0.002

Values are represented as mean ± standard deviation or number (%); ACEis, angiotensin-converting enzyme inhibitors; AGIs, α-glucosidase Inhibitors; ARBs, angiotensin II, receptor blockers; CABG, coronary artery bypass graft; CAD, coronary artery disease; COPD, chronic obstructive pulmonary disease; CV, cardiovascular; DM, diabetes mellitus; HONK, hyperglycaemic hyperosmolar nonketotic coma; HTN, hypertensive; MI, myocardial infarction; NSAIDs, non-steroidal anti-inflammatory drugs; PAD, peripheral artery disease; PUD, peptic ulcer disease; STD, standardized difference; SUs, sulfonylureas; UC, ulcerative colitis.

After 11,563 empagliflozin users were matched to sitagliptin users, the above differences (age, number of clinic visits, index date, cardiovascular risk factors, comedications, and comorbidities) were reduced, and both groups were well balanced. Standardized differences were well below 0.1 for all 72 covariates. Median length of follow-up (2.7 years; median duration of anti-diabetic medications prescription during follow-up [1.7 (interquartile range 0.5–2.4) years]; and mean age of patients [55.6 years; men: 58.9% (n = 13,628)] were shown. In the other nine study cohorts for evaluating safety outcomes, the two drug user groups were also well balanced after 1:1 matching (Supplementary Tables S5–S13).

### Comparison of baseline characteristics between RCT and RWE

A lower proportion of men and a lower mean age were observed in our RWE cohort than in the corresponding RCT. (Table 2). Compared to the RCT, the RWE cohort was more often diagnosed with coronary artery disease (including coronary revascularization) and had fewer MIs, strokes, and peripheral artery disease. Rates of patients receiving glucose-lowering therapies were generally similar between the RCT and the RWE, except for the use of insulin. However, the proportions of patients who have been more than 5 years since their diagnosis of T2DM were 82.0 and 6.0% in RCT and RWE, respectively (*p-*value < 0.001).

**TABLE 2:** Comparison of baseline characteristics between RCT and RWE.

Characteristics	EMPA-REG outcome^®^ (RCT)		EMPA-REG Duplicate (RWE)	
	**Placebo N = 2,333**	**Empagliflozin N = 4,687**	**Sitagliptin N = 11,563**	**Empagliflozin N = 11,563**
Age	63.2 ± 8.8	63.1 ± 8.6	55.5 ± 11.3	55.6 ± 10.9
Male—no. (%)	1,680 (72.0)	3,336 (71.2)	6,799 (58.8)	6,829 (59.1)
CV risk factor				
Coronary artery disease	1763 (75.6)	3,545 (75.6)	10,657 (92.2)	10,699 (92.5)
Multi-vessel coronary artery disease	1,100 (47.1)	2,179 (46.5)	5,944 (51.4)	6,056 (52.4)
History of myocardial infarction	1,083 (46.4)	2,190 (46.7)	836 (7.2)	877 (7.6)
Coronary artery bypass graft	563 (24.1)	1,175 (25.1)	3,195 (27.6)	3,264 (28.2)
History of stroke	553 (23.7)	1,084 (23.1)	1,030 (8.9)	1,057 (9.1)
Peripheral artery disease	479 (20.5)	982 (21.0)	687 (5.9)	661 (5.7)
Glucose-lowering therapy				
Metformin	1734 (74.3)	3,459 (73.8)	8,422 (72.8)	8,382 (72.5)
Insulin	1,135 (48.6)	2,252 (48.0)	2098 (18.1)	2074 (17.9)
Sulfonylurea	992 (42.5)	2014 (43.0)	5,428 (46.9)	5,441 (47.1)
Thiazolidinedione	101 (4.3)	198 (4.2)	1,309 (11.3)	1,301 (11.3)
Glucagon-like peptide-1 agonist	70 (3.0)	126 (2.7)	78 (0.7)	74 (0.6)
Time since diagnosis of type 2 diabetes				
≤1 year	52 (2.2)	128 (2.7)	5,043 (43.6)	5,008 (43.3)
>1 to 5 years	371 (15.9)	712 (15.2)	5,827 (50.4)	5,866 (50.7)
>5 years	1910 (81.9)	3,847 (82.1)	693 (6.0)	689 (6.0)
Anti-hypertensives	2,221 (95.2)	4,446 (94.9)	9,625 (83.2)	9,685 (83.8)
Diuretics	988 (42.3)	2047 (43.7)	3,689 (31.9)	3,698 (32.0)
Lipid-lowering	1864 (79.9)	3,820 (81.5)	9,616 (83.2)	9,655 (83.5)
Anti-coagulants	2090 (89.6)	4,162 (88.8)	8,351 (72.2)	8,422 (72.8)

Values are represented as mean ± standard deviation or number (%); CV, cardiovascular; RCT, randomized clinical trial; RWE, real-world evidence.

### RCT-RWE agreement on cardiovascular outcomes

**FIGURE 2:**
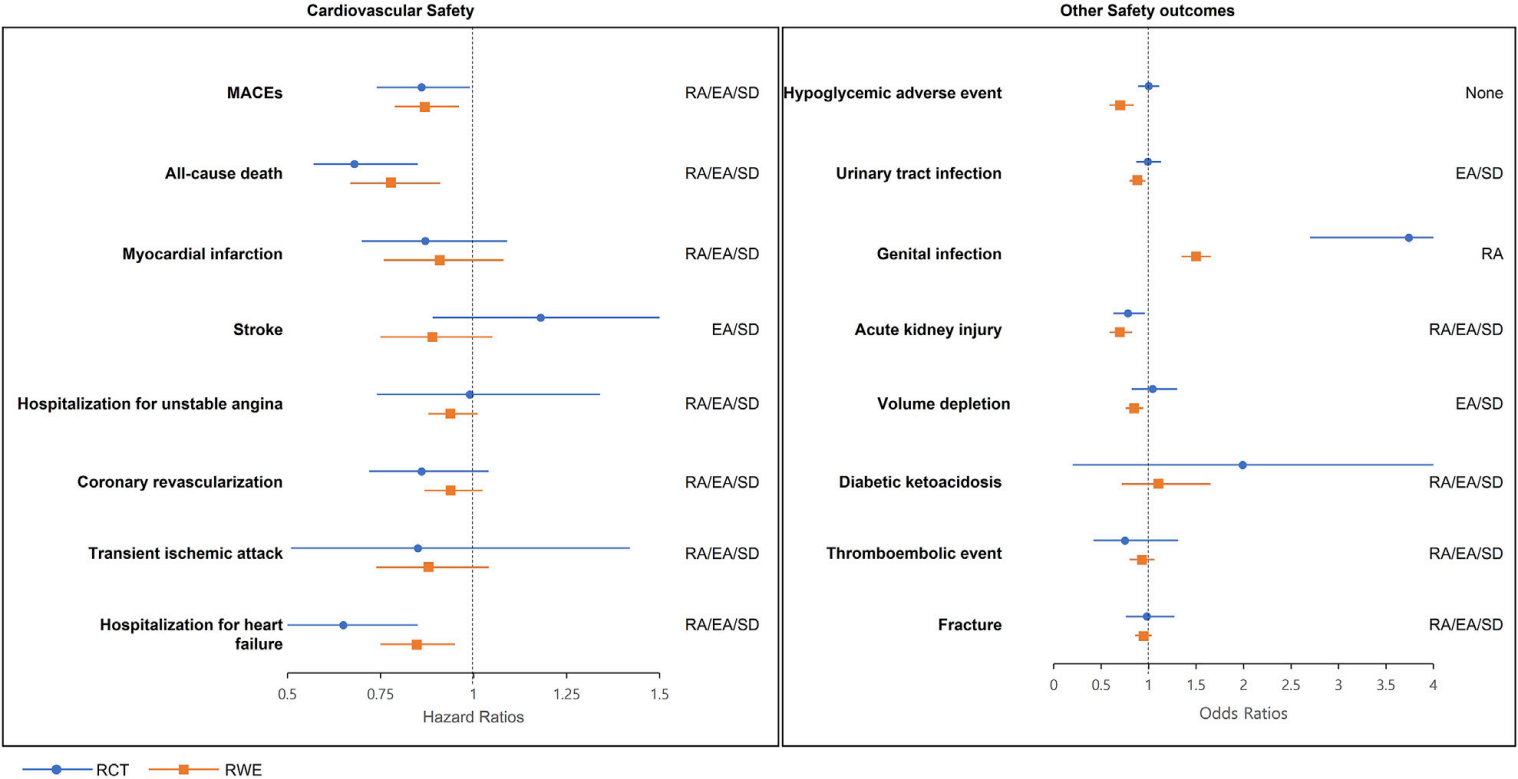
RCT-RWE agreements plots. EA, estimate agreement; MACEs, major adverse cardiovascular events; RA, regulatory agreement; RCT, randomized clinical trial; RWE, real-world evidence; SD, standardized difference.

From the results of RWE, empagliflozin was associated with a significantly decreased risk of MACEs (HR 0.87, 95% CI 0.79–0.96), all-cause mortality (HR 0.78, 95% CI 0.67–0.91), and heart failure (HR 0.85, 95% CI 0.75–0.95) comparing to sitagliptin (Table 3). MI, stroke, hospitalization for unstable angina, coronary revascularization, and transient ischemic attack were not significantly associated with empagliflozin use. As mentioned above, empagliflozin was related to a significantly decreased risk of MACEs [EMPA-REG DUPLICATE RWE: adjusted HR 0.87, 95% confidence interval (CI) 0.79–0.96]. The predifined estimate agreement, regulatory agreement, and standardized difference for RCT duplication were achieved (Figure 2) [EMPA-REG OUTCOME RCT: adjusted HR 0.86, 95% (CI) 0.74–0.99]. All of the eight cardiovascular outcomes except stroke achieved three agreements (RA/EA/SD) (point estimate HR in RCT and RWE = 0.86:0.87 [MACEs], 0.68:0.78 [all-cause death], 0.87:0.91 [MI], 0.99:0.94 [hospitalization for unstable angina], 0.86:0.94 [coronary revascularization], 0.85:0.88 [transient ischemic attack], and 0.65:0.85 [hospitalization for heart failure]). For stroke, the HR estimate of RWE 0.89 was in the opposite direction to that of RCT (disagreement of RA [point estimate HR of RCT: 1.18]), and two of three agreements (EA/SD) were achieved.

**TABLE 3:** RCT-RWE agreements for MACEs and each cardiovascular outcome component.

Outcomes	EMPA-REG Outcome^®^ (RCT)		EMPA-REG Duplicate (RWE)		STD	Agreement		
	Rate/1,000 Patient-yr	HR (95%CI)	Rate/1,000 Patient-yr	HR (95%CI)		RA	EA	SD
*MACEs*								
Sitagliptin	43.9	0.86 (0.74–0.99)	25.5	0.87 (0.79–0.96)	0.1	Y	Y	Y
Empagliflozin	37.4		22.5					
*All-cause death*								
Sitagliptin	28.6	0.68 (0.57–0.82)	12.0	0.78 (0.67–0.91)	1.0	Y	Y	Y
Empagliflozin	19.4		9.5					
*Myocardial infarction*								
Sitagliptin	19.3	0.87 (0.70–1.09)	8.7	0.91 (0.76–1.08)	0.3	Y	Y	Y
Empagliflozin	16.8		7.9					
*Stroke*								
Sitagliptin	10.5	1.18 (0.89–1.56)	9.1	0.89 (0.75–1.05)	−1.7	N	Y	Y
Empagliflozin	12.3		8.2					
*Hospitalization for unstable angina*								
Sitagliptin	10.0	0.99 (0.74–1.34)	50.5	0.94 (0.88–1.01)	−0.3	Y	Y	Y
Empagliflozin	10.0		48.1					
*Coronary revascularization*								
Sitagliptin	29.1	0.86 (0.72–1.04)	36.9	0.94 (0.87–1.02)	0.8	Y	Y	Y
Empagliflozin	25.1		35.2					
*Transient ischemic attack*								
Sitagliptin	3.5	0.85 (0.51–1.42)	9.2	0.88 (0.74–1.04)	0.1	Y	Y	Y
Empagliflozin	2.9		8.0					
*Hospitalization for heart failure*								
Sitagliptin	14.5	0.65 (0.50–0.85)	20.5	0.85 (0.75–0.95)	1.8	Y	Y	Y
Empagliflozin	9.4		17.4					

EA, estimate agreement; HR, hazard ratio; CI, confidence interval; MACEs, major adverse cardiovascular events; RA, regulatory agreement; RCT, randomized clinical trial; RWE, real-world evidence; SD, standardized difference; STD, standardized difference; Y, yes; N, no.

### RCT-RWE agreement of safety outcomes

For safety outcomes from RWE, empagliflozin was associated with lowered risk of hypoglycemia (OR 0.70, 95% CI 0.59–0.84), UTI (OR 0.87, 95% CI 0.81–0.94), AKI (OR 0.70, 95% CI 0.59–0.82), and volume depletion (OR 0.84, 95% CI 0.76–0.94) comparing to sitagliptin. Alternatively, the risk of genital infections significantly increased (OR 1.49, 95% CI 1.35–1.65) compared to sitagliptin. No significant associations were identified in DKA, thromboembolic event, and fracture (Table 4).

**TABLE 4:** RCT-RWE agreement for each safety outcome.

Outcomes	EMPA-REG Outcome^®^ (RCT)		EMPA-REG Duplicate (RWE)		STD	Agreement		
	Rate (%)	OR (95%CI)	Rate (%)	OR (95%CI)		RA	EA	SD
*Hypoglycemic adverse event*								
Sitagliptin	27.9	1.00 (0.89–1.11)	2.6	0.70 (0.59–0.84)	-3.3	N	N	N
Empagliflozin	27.8		1.9					
*Urinary tract infection*								
Sitagliptin	18.1	0.99 (0.87–1.13)	23.3	0.87 (0.81–0.94)	-1.7	N	Y	Y
Empagliflozin	18.0		20.9					
*Genital infection*								
Sitagliptin	1.8	3.74 (2.70–5.19)	7.9	1.49 (1.35–1.65)	-5.3	Y	N	N
Empagliflozin	6.4		11.4					
*Acute kidney injury*								
Sitagliptin	6.6	0.78 (0.63–0.96)	3.3	0.70 (0.59–0.82)	-0.8	Y	Y	Y
Empagliflozin	5.2		2.3					
*Volume depletion*								
Sitagliptin	4.9	1.04 (0.82–1.30)	7.1	0.84 (0.76–0.94)	-1.7	N	Y	Y
Empagliflozin	5.1		6.1					
*Diabetic ketoacidosis*								
Sitagliptin	0.04	1.99 (0.2–17.8)	0.38	1.09 (0.72–1.64)	-0.5	Y	Y	Y
Empagliflozin	0.1		0.42					
*Thromboembolic event*								
Sitagliptin	0.9	0.75 (0.42–1.31)	4.3	0.92 (0.80–1.05)	0.7	Y	Y	Y
Empagliflozin	0.6		3.9					
*Fracture*								
Sitagliptin	3.9	0.98 (0.76–1.27)	13.8	0.94 (0.87–1.03)	-0.3	Y	Y	Y
Empagliflozin	3.8		13.1					

EA, estimate agreement; HR, hazard ratio; CI, confidence interval; MACEs, major adverse cardiovascular events; RA, regulatory agreement; RCT, randomized clinical trial; RWE, real-world evidence; SD, standardized difference; STD, standardized difference; Y, yes; N, no.

In regulatory agreement, empagliflozin showed significantly lowered risk in RWE, whereas the RCT reported a non-significant effect on the hypoglycemic adverse event, UTI, and volume depletion. An estimate agreement was achieved for 6 of the 8 emulations, with the exception of a hypoglycemic adverse event (OR: 0.70) and genital infections (OR: 1.49) where the emulation estimates were below the lower 95% CI bound from the RCT (OR: 1.00; 95% CI: 0.89–1.11 and OR: 3.74; 95% CI: 2.70–5.19 for hypoglycemic adverse event and genital infection, respectively). Statistically significant disagreements in SDs were shown (SD: −3.3 and −5.3 for hypoglycemic adverse event and genital infections, respectively).

### Sensitivity analyses

After follow-up of patients who received at least one dose of study drugs until ≤30 days after the last intake of medication, similar results (HR for MACEs: 0.88; 95% CI: 0.77–0.99) were obtained (Supplementary Table S14). Additional sensitivity analysis (including patients who received study drugs for ≥30 days including only events that occurred ≤30 days after a patient’s last intake of medications) did not produce meaningful changes in the study findings (HR for MACEs: 0.87; 95% CI: 0.79–0.96) (Supplementary Table S15). All three agreements remained ‘Y’ for MACEs in both sensitivity analyses. In the same sensitivity analyses for eight safety outcomes, at least two of the three agreements were achieved in six safety outcomes (UTI, AKI, volume depletion, DKA, thromboembolic event, and fracture) (Supplementary Tables S16, S17). The hypoglycemic adverse event and genital infections still failed to show sufficient agreements, as in the main analysis.

## Discussion

Our study analyzed patients with high cardiovascular disease risks that were prescribed empagliflozin or sitagliptin for emulation of a pre-existing RCT. The primary objective of the study was to evaluate to what extent the safety of empagliflozin from the RWE study in Korea is different from the one in RCT by emulating the design of foreign RCT. This study emulated the cardiovascular outcomes including other safety outcomes of the EMPA-REG OUTCOME RCT in Korea. According to pre-specified agreement standards, successful agreements were achieved in cardiovascular disease including MACEs. For all outcomes, 14 of the 16 RCT outcomes including safety outcomes were successfully reproduced (graded as “good” or “moderate”). Our study results suggested that RWE can emulate RCT results satisfactorily and have the potential for providing evidence for future regulatory decision-making when RCT evidence is not available in Korea.

As shown in other studies, one must always keep in mind that some discrepancies may occur due to differences in study samples, study designs, or statistical methods. To date, various RWE studies have reported on the safety of SGLT-2 inhibitors including empagliflozin. There were discrepancies between findings, for example, the beneficial effect of SGLT-inhibitors on MACEs has been reported (Persson et al., 2018; Filion et al., 2020; Dave et al., 2021). However, two other studies have reported non-significant results in MACEs (Norhammar et al., 2019; Jeon et al., 2021). In other safety outcomes, Lega *et al.* reported a decreased risk of UTIs (Lega et al., 2019), while another study reported an association with an increased risk of UTI (Han et al., 2021). SGLT2 inhibitor use was associated with an elevated DKA risk (Wang et al., 2019); however, this study was not in Korea (Kim et al., 2018). We found both adverse (Ueda et al., 2018) and beneficial (Toulis et al., 2018) effects on fracture, although most results were non-significant. Most studies have reported decreased risks of SGLT2 inhibitors on AKI or impairment in renal function (Nadkarni et al., 2017; Cahn et al., 2019; Heerspink et al., 2020; Koh et al., 2021). Therefore, our study focused on emulating an existing RCT design and thereby confirming that the same results can be obtained from RWE. We have demonstrated SGLT-2 inhibitors’ associations with decreased cardiovascular outcomes including reducing MACEs and heart failure. Our results were consistent with the results of the target trial, and other studies including RCTs [MACEs (Mascolo et al., 2021) and heart failure (Kramer et al., 2010; Mascolo et al., 2021; Requena-Ibanez et al., 2021; Santos-Gallego et al., 2021; Ferreira et al., 2022; Neuen et al., 2022; Requena-Ibanez et al., 2022; Sauer, 2022)] which show that SGLT could induce reverse cardiac remodeling and improving quality of life, and also reduce myocardial fibrosis.

However, despite the substantial effort, there were disagreements between the RCT and RWE in several outcomes. Stroke is a well-known disease that can be captured with a high accuracy because of its seriousness. The incidence rates were similar between RCT and RWE results. However, our study result suggested that empagliflozin was associated with a decreased risk of stroke (although not significant) unlike its non-significant increase in the RCT. Several meta-analyses including all trials do show reductions in hemorrhagic stroke (Tsai et al., 2021) and in total stroke (Mascolo et al., 2021), which supports our results. Also, SGLT-2 inhibitors seem to reduce atrial fibrillation (Pandey et al., 2021), which can also explain the stroke protection. It seems reason for the discrepancy is not clear. Ethnic factors may have been involved because over 70% of patients were Caucasian, and only 20% were Asian in the RCT (Zinman et al., 2015). Asians are reported to have a lower risk of cardiovascular disease than other races (Jung et al., 2015). As this study was conducted on Koreans, the proportion of patients with a history of severe diseases such as MI or stroke was small at baseline, and the age and severity of diabetes (time since onset of T2DM) were also lower than those of the RCT. In the subgroup analysis reported by the RCT, empagliflozin was reported to have a HR of 0.88 and 1.48 for Caucasians and Blacks for MACEs respectively and 0.68 for Asians (Zinman et al., 2015). Another study showed the protective effect of the SGLT-2 inhibitors against stroke in Koreans (Han et al., 2021); therefore, racial factors may have influenced our findings.

Another hypothesis includes a possibility of physicians’ reluctance to prescribe empagliflozin because of its known side effects. It has been reported that cardiologists may be reluctant to prescribe SGLT2 inhibitors due to concerns of adverse effects (Vardeny and Vaduganathan, 2019). Owing to incomplete knowledge of its benefits and/or risks (Das et al., 2018), concerns with SGLT2 inhibitors have led to decreased use in clinical practice (Vaduganathan et al., 2018). The drug approval date of empagliflozin was May 2016 in Korea, and physicians may have paid attention to prescription in the early stages of approval during the index period (2016–2018) of this study. Typically, patients tend not to use drugs when they are not in good health (Glynn et al., 2001) and this phenomenon can be observed in a study that reported excessively large protective effects on cardiovascular disease by using statins (Glynn et al., 2006). In the case of a new drug, this point should be taken into account because physicians often intend to prescribe the medication to a person who is expected to be relatively healthy and has a good prognosis. This trend is expected to be more prominent in outcomes such as stroke and genital infection in which the point estimate was reported as one or higher in RCTs. The HR point estimate of such an outcome in RWE is either reversed or much lower than the value reported in the RCT. Stroke and genital infection showed HRs and ORs of 1.18 and 3.74 in the RCT, and 0.89 and 1.49 in our RWE study, respectively. Therefore, there is a possibility that undetected selection bias exists in our study.

In the hypoglycemic event, there was a >10-fold difference between the incidence in a RCT and that in RWE. The hypoglycemic event was less likely to be captured in real-world claim data, as shown in the event rates. Kim et al. reported that there is a possibility of underestimating the frequency of the hypoglycemic events when using HIRA data (Kim et al., 2016). Other studies share similar problems, showing the accuracy of diagnosis could be low owing to the nature of claims data because hypoglycemic events that can be self-treated do not need any medical management (Task Force Team for Basic Statistical Study of Korean Diabetes Mellitus of Korean Diabetes Association et al., 2013; Park et al., 2018). It appears that physicians in Korea consider hypoglycemic events to be temporary and do not often record a diagnostic code. Similarly, two observational studies in Korea showed low event rates of hypoglycemia (6.3%, self-reported outcome) (Hong et al., 2019), and 2.4 per 100 person-year (insurance claim data) (Han et al., 2021). The discrepancy in event rates could have led to the disagreement in treatment effect estimates. The event rate appears to be an important factor when conducting the RCT emulation study.

The intention-to-treat approach was applied in our study, and the median duration of observation time was 2.7 and 3.1 years in RWE and RCT studies, respectively. Adherence to medications in the RWE is often poor compared with the RCT (Freemantle et al., 2013), and the median duration of treatment was 1.7 (RWE) and 2.6 (RCT) years in this study. In sensitivity analysis, as-treatment analyses were performed to test whether our main outcome was affected by adherence. Similar results were obtained, and shorter duration of use for empagliflozin provided a benefit on several outcomes.

There are several limitations in our study. We tried to emulate as much of an RCT as possible, including inclusion and exclusion criteria, exposures, and results; however, because of the limitations of the healthcare database, accurate emulation was not possible. Our study is a retrospective cohort design and not all information is included in the HIRA data (e.g., lab results for blood glucose test, urine culture test, or body weight). Therefore, although we adjusted for all possible confounders, there still may be residual confounding factors present. There were regulatory disagreements in UTIs and volume depletion outcomes, indicating potential for residual confounding factors related to these outcomes. Additionally, note that unlike RCT, RWE cannot provide the exact cause and effect, and it could only show a significant association. The ultimate goal of our study was to utilize relevant RWE for regulatory decisions when no RCT evidence is available. The results of RCT and RWE are not always consistent. As mentioned above, event rates for testing specificity of outcome definition should be addressed. In addition, consideration of characteristics such as study participants, real-world clinical settings, and data availability might be important for enhancing the validity of study.

Our study results suggest that RWE emulating foreign RCT has the potential for providing evidence for future regulatory decision-making in an environment where RCT could not be performed. Further research is needed to determine whether RWE findings can be reliable evidence in various clinical settings or specific patient groups.

## Data Availability

The datasets presented in this article are not readily available because Viewing of data that shows all the records of a patient are difficult to share owing to the policy of the Korean National Health Insurance Service. It can only be viewed in anonymized form when analyzed. Therefore, if there is a request for original data, the statistical data obtained after the desired statistical processing on the server will be shared. Requests to access the datasets should be directed to National Health Insurance Service, nhiss. nhis.or.kr.
