# Supplementary material for: Using real-world data for supporting regulatory decision making: Comparison of cardiovascular and safety outcomes of an empagliflozin randomized clinical trial versus real-world data
Source: Front Pharmacol. 2022 Aug 30;13:928121. doi: 10.3389/fphar.2022.928121 (PMC9468970; doi:10.3389/fphar.2022.928121)
Supplement: Supplementary file 1 [file DataSheet1.docx]

**
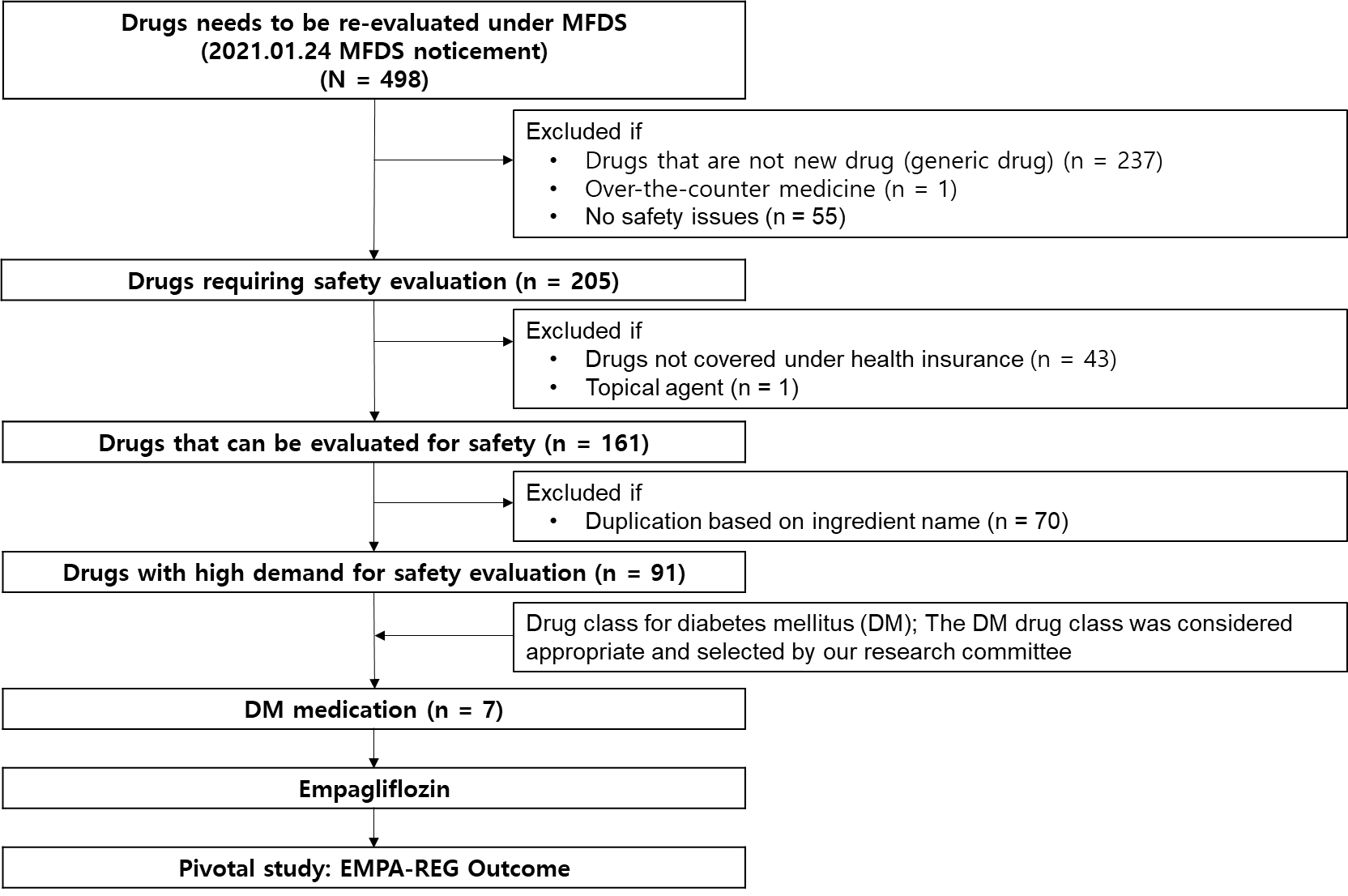
**

**Supplementary Figure S1 Flow chart for study drug selection. MFDS, Ministry of Food and Drug Safety**

**Supplementary Table S1 Candidate study drugs for selection in Korea**

|  | **Drug** | **Approval date** | **Start date of re-evaluation** | **End date of re-evaluation** | **ATC code** |
| --- | --- | --- | --- | --- | --- |
| 1 | cinacalcet | 2010-03-04 | 2010-03-04 | 2016-03-03 | H05BX01 |
| 2 | abatacept | 2010-03-29 | 2010-03-29 | 2016-03-28 | L04AA24 |
| 3 | paliperidone | 2010-07-26 | 2010-07-26 | 2016-07-25 | N05AX13 |
| 4 | prasugrel | 2010-07-29 | 2010-07-29 | 2016-07-28 | B01AC22 |
| 5 | azacitidine | 2006-01-27 | 2010-08-06 | 2016-08-05 | L01BC07 |
| 6 | pazopanib | 2010-08-11 | 2010-08-11 | 2016-08-10 | L01XE11 |
| 7 | dasatinib | 2007-01-25 | 2010-08-20 | 2016-08-19 | L01XE06 |
| 8 | indacaterol | 2010-08-26 | 2010-08-26 | 2016-08-25 | R03AC18 |
| 9 | darunavir | 2010-10-29 | 2010-10-29 | 2016-10-28 | J05AE10 |
| 10 | nilotinib | 2010-12-24 | 2010-12-24 | 2016-12-23 | L01XE08 |
| 11 | saxagliptin | 2011-01-24 | 2011-01-24 | 2017-01-23 | A10BH03 |
| 12 | dabigatran etexilate | 2011-02-18 | 2011-02-18 | 2017-02-17 | B01AE07 |
| 13 | lenalidomide | 2009-12-30 | 2011-03-09 | 2017-03-08 | L04AX04 |
| 14 | tenofovir disoproxil | 2010-06-23 | 2011-04-29 | 2017-04-28 | J05AF07 |
| 15 | roflumilast | 2011-05-16 | 2011-05-16 | 2017-05-15 | R03DX07 |
| 16 | ustekinumab | 2011-06-21 | 2011-06-21 | 2017-06-20 | L04AC05 |
| 17 | aprepitant | 2011-07-14 | 2011-07-14 | 2017-07-13 | A04AD12 |
| 18 | ticagrelor | 2011-07-22 | 2011-07-22 | 2017-07-21 | B01AC24 |
| 19 | tolvaptan | 2011-09-01 | 2011-09-01 | 2017-08-31 | C03XA01 |
| **20** | **linagliptin** | **2011-09-14** | **2011-09-14** | **2017-09-13** | **A10BH05** |
| 21 | apixaban | 2011-11-30 | 2011-11-30 | 2017-11-29 | B01AF02 |
| 22 | rilpivirine | 2012-01-31 | 2012-01-31 | 2018-01-30 | J05AG05 |
| 23 | tocilizumab | 2012-04-06 | 2012-04-06 | 2018-04-05 | L04AC07 |
| 24 | golimumab | 2012-08-14 | 2012-08-14 | 2018-08-13 | L04AB06 |
| 25 | eribulin | 2012-08-17 | 2012-08-17 | 2018-08-16 | L01XX41 |
| 26 | axitinib | 2012-08-22 | 2012-08-22 | 2018-08-21 | L01XE17 |
| 27 | aflibercept | 2013-03-20 | 2013-03-20 | 2019-03-19 | S01LA05 |
| 28 | degarelix | 2013-04-18 | 2013-04-18 | 2019-04-17 | L02BX02 |
| 29 | pertuzumab | 2013-05-29 | 2013-05-29 | 2019-05-29 | L01XC13 |
| **30** | **alogliptin** | **2013-05-31** | **2013-05-31** | **2019-05-30** | **A10BH04** |
| 31 | regorafenib | 2013-08-22 | 2013-08-22 | 2019-08-21 | L01XE21 |
| 32 | rasagiline | 2013-09-13 | 2013-09-13 | 2019-09-12 | N04BD02 |
| **33** | **dapagliflozin** | **2013-11-26** | **2013-11-26** | **2019-11-25** | **A10BK01** |
| 34 | mirabegron | 2013-12-31 | 2013-12-31 | 2019-12-30 | G04BD12 |
| 35 | trastuzumab emtansine | 2014-01-24 | 2014-01-24 | 2020-01-23 | L01XC14 |
| 36 | afatinib | 2014-01-29 | 2014-01-29 | 2020-01-28 | L01XE13 |
| 37 | desvenlafaxine | 2014-02-06 | 2014-02-06 | 2020-02-05 | N06AX23 |
| **38** | **insulin degludec** | **2014-03-20** | **2014-03-20** | **2020-03-19** | **A10AE06** |
| **39** | **insulin degludec/insulin aspart** | **2014-03-20** | **2014-03-20** | **2020-03-19** | **A10AD06** |
| 40 | tofacitinib | 2014-04-02 | 2014-04-02 | 2020-04-01 | L04AA29 |
| 41 | bendamustine | 2011-05-31 | 2014-06-16 | 2020-06-16 | L01AA09 |
| 42 | aclidinium bromide | 2014-06-30 | 2014-06-30 | 2020-06-29 | R03BB05 |
| 43 | vilanterol and fluticasone furoate | 2014-07-01 | 2014-07-01 | 2020-06-30 | R03AK10 |
| 44 | vilanterol and umeclidinium bromide | 2014-07-10 | 2014-07-10 | 2020-07-09 | R03AL03 |
| **45** | **empagliflozin** | **2014-08-12** | **2014-08-12** | **2020-08-11** | **A10BK03** |
| 46 | vortioxetine | 2014-08-19 | 2014-08-19 | 2020-08-18 | N06AX26 |
| 47 | dolutegravir | 2014-08-29 | 2014-08-29 | 2020-08-28 | J05AX12 |
| 48 | denosumab | 2014-09-29 | 2014-09-29 | 2020-09-28 | M05BX04 |
| 49 | macitentan | 2014-11-03 | 2014-11-03 | 2020-11-02 | C02KX04 |
| 50 | ramucirumab | 2015-04-15 | 2015-04-15 | 2021-04-14 | L01XC21 |
| 51 | daclatasvir | 2015-04-28 | 2015-04-28 | 2021-04-27 | J05AP07 |
| 52 | enzalutamide | 2013-06-26 | 2015-05-22 | 2021-05-21 | L02BB |
| 53 | dulaglutide | 2015-05-26 | 2015-05-26 | 2021-05-25 | A10BJ05 |
| 54 | perampanel | 2015-07-10 | 2015-07-10 | 2021-07-09 | N03AX22 |
| 55 | edoxaban | 2015-08-25 | 2015-08-25 | 2021-08-24 | B01AF03 |
| 56 | ambrisentan | 2009-04-20 | 2015-08-31 | 2021-08-30 | C02KX02 |
| 57 | olodaterol and tiotropiumbromide | 2015-08-31 | 2015-08-31 | 2021-08-30 | R03AL06 |
| 58 | sofosbuvir | 2015-09-10 | 2015-09-10 | 2021-09-09 | J05AP08 |
| 59 | secukinumab | 2015-09-24 | 2015-09-24 | 2021-09-23 | L04AC10 |
| 60 | lenvatinib | 2015-10-07 | 2015-10-07 | 2021-10-06 | L01XE29 |
| 61 | sofosbuvir and ledipasvir | 2015-10-13 | 2015-10-13 | 2021-10-12 | J05AP51 |
| 62 | dimethyl fumarate | 2016-01-22 | 2016-01-22 | 2022-01-21 | L04AX07 |
| 63 | nivolumab | 2015-03-20 | 2016-04-01 | 2022-03-31 | L01XC17 |
| 64 | valsartan and sacubitril | 2016-04-14 | 2016-04-14 | 2022-04-13 | C09DX04 |
| 65 | pembrolizumab | 2015-03-20 | 2016-04-29 | 2022-04-28 | L01XC18 |
| 66 | olmutinib | 2016-05-13 | 2016-05-13 | 2022-05-12 | L01XE40 |
| 67 | palbociclib | 2016-08-29 | 2016-08-29 | 2022-08-28 | L01XE33 |
| 68 | emtricitabine, tenofovir alafenamide, elvitegravir and cobicistat | 2016-09-13 | 2016-09-13 | 2022-09-12 | J05AR18 |
| 69 | elbasvir and grazoprevir | 2016-11-21 | 2016-11-21 | 2022-11-20 | J05AP54 |
| 70 | ruxolitinib | 2013-01-21 | 2016-11-29 | 2022-11-28 | L01XE18 |
| 71 | pasireotide | 2016-12-20 | 2016-12-20 | 2022-12-19 | H01CB05 |
| 72 | atezolizumab | 2017-01-12 | 2017-01-12 | 2023-01-11 | L01XC32 |
| 73 | carfilzomib | 2017-03-31 | 2017-03-31 | 2023-03-30 | L01XX45 |
| 74 | azilsartan medoxomil | 2017-05-26 | 2017-05-26 | 2023-05-25 | C09CA09 |
| 75 | pomalidomide | 2014-08-04 | 2017-06-09 | 2023-06-08 | L04AX06 |
| 76 | cabozantinib | 2017-09-26 | 2017-09-26 | 2023-09-25 | L01XE26 |
| 77 | baricitinib | 2017-12-11 | 2017-12-11 | 2023-12-10 | L04AA37 |
| 78 | glecaprevir and pibrentasvir | 2018-01-12 | 2018-01-12 | 2024-01-11 | J05AP57 |
| 79 | sucroferric oxyhydroxide | 2018-03-20 | 2018-03-20 | 2024-03-19 | V03AE05 |
| 80 | dupilumab | 2018-03-30 | 2018-03-30 | 2024-03-29 | D11AH05 |
| 81 | guselkumab | 2018-04-12 | 2018-04-12 | 2024-04-11 | L04AC16 |
| **82** | **ertugliflozin** | **2018-08-17** | **2018-08-17** | **2024-08-16** | **A10BK04** |
| 83 | durvalumab | 2018-12-04 | 2018-12-04 | 2024-12-03 | L01XC28 |
| 84 | osimertinib | 2016-05-19 | 2018-12-26 | 2024-12-25 | L01XE35 |
| 85 | emtricitabine, tenofovir alafenamide and bictegravir | 2019-01-18 | 2019-01-18 | 2025-01-17 | J05AR20 |
| 86 | abemaciclib | 2019-05-01 | 2019-05-01 | 2025-04-30 | L01XE50 |
| 87 | eliglustat | 2015-11-12 | 2019-10-02 | 2025-10-01 | A16AX10 |
| 88 | ribociclib | 2019-10-30 | 2019-10-30 | 2025-10-29 | L01XE42 |
| 89 | vedolizumab | 2015-06-19 | 2020-01-15 | 2026-01-14 | L04AA33 |
| 90 | venetoclax | 2019-05-29 | 2020-03-26 | 2026-03-25 | L01XX52 |
| 91 | brigatinib | 2018-11-30 | 2020-08-27 | 2026-08-26 | L01XE43 |

Anatomical Therapeutic Chemical, ATC

**Supplementary Table S2 Diabetes mellitus candidate study drugs**

| **Drugs** | **Warnings and precautions on FDA drug label** |
| --- | --- |
| saxagliptin | 1. Pancreatitis 2. Heart Failure 3. Hypoglycemia with Concomitant Use of Sulfonylurea or Insulin 4. Hypersensitivity Reactions 5. Severe and Disabling Arthralgia 6. Bullous Pemphigoid 7. Macrovascular Outcomes |
| linagliptin | 1. Pancreatitis 2. Heart Failure 3. Use with Medications Known to Cause Hypoglycemia 4. Hypersensitivity Reactions 5. Severe and Disabling Arthralgia 6. Bullous Pemphigoid |
| alogliptin | 1. Pancreatitis 2. Heart Failure 3. Hypersensitivity Reactions 4. Hepatic Effects 5. Use with Medications Known to Cause Hypoglycemia 6. Severe and Disabling Arthralgia 7. Bullous Pemphigoid 8. Macrovascular Outcomes |
| dapagliflozin | 1. Volume Depletion 2. Ketoacidosis in Patients with Diabetes Mellitus 3. Urosepsis and Pyelonephritis 4. Hypoglycemia with Concomitant Use with Insulin and Insulin Secretagogues 5. Necrotizing Fasciitis of the Perineum (Fournier’s Gangrene) 6. Genital Mycotic Infections |
| insulin degludec | 1. Never Share a TRESIBA Flex Touch Pen, Needle, or Syringe Between Patients 2. Hyperglycemia or Hypoglycemia with Changes in Insulin Regimen 3. Hypoglycemia 4. Hypoglycemia Due to Medication Errors 5. Hypersensitivity and Allergic Reactions 6. Hypokalemia 7. Fluid Retention and Congestive Heart Failure with Concomitant Use of a PPAR Gamma Agonist |
| **empagliflozin** | 1. **Hypotension** 2. **Ketoacidosis** 3. **Acute Kidney Injury and Impairment in Renal Function** 4. **Urosepsis and Pyelonephritis** 5. **Hypoglycemia with Concomitant Use with Insulin and Insulin Secretagogues** 6. **Necrotizing Fasciitis of the Perineum (Fournier’s Gangrene)** 7. **Genital Mycotic Infections** 8. **Hypersensitivity Reactions** 9. **Increased Low-Density Lipoprotein Cholesterol (LDL-C)** |
| ▼ | |
| SGLT2 inhibitors are attracting its attention because they have a drug mechanism that shows an insulin-independent effect of lowering blood sugar. Specific adverse events such as urinary tract infection, genital infection, and renal dysfunction have been confirmed from its clinical trials. In addition, diabetic ketoacidosis, pyelonephritis, urinary sepsis, and netrotizing fasciitis were reported in overseas post-marketing research.  In December 2016, a new indication was added to the FDA approval of empagliflozin, 'Reducing the risk of cardiovascular death in patients with type 2 diabetes with cardiovascular disease'. As the usage of the empagliflozin increases, the interest in safety is also increasing. Comprehensively, empagliflozin was selected as an analysis target among the drugs that have been re-examined. | |

Food and Drug Administration, FDA; sodium-glucose cotransporter 2, SGLT2

**Supplementary Table S3 Operational definitions of inclusion/exclusion criteria**

| **Criteria in EMPA-REG outcome trial** | | **Criteria in EMPA-REG duplicate study** | |
| --- | --- | --- | --- |
| **Inclusion criteria** | | | |
| Diagnosis of type 2 diabetes mellitus prior to informed consent | | History of diabetes mellitus (E11-E14) | |
| Male or female patients on diet and exercise regimen who are drug naive or pre-treated with any background therapy. Antidiabetic therapy has to be unchanged for 12 weeks prior to randomization. | | Drug (empagliflozin, sitagliptin)-naïve patients | |
| Glycosylated haemoglobin (HbA1c) of >= 7.0% and <=10% for patients on background therapy or HbA1c >= 7.0% and <= 9.0% for drug naive patients | | - | |
| Age >= 18 years | | Age >= 18 years | |
| Body Mass index <= 45 at Visit 1 | | - | |
| Signed and dated informed consent | | - | |
| High cardiovascular risk | |  | |
| History of myocardial infarction >2 months prior to informed consent | | History of Myocardial infarction (I20) >2 months prior to index date | |
| Evidence of multi-vessel coronary artery disease i.e. in ≥2 major coronary arteries or the left main coronary artery, documented by any of the following: | |  | |
| Presence of significant stenosis: ≥50% luminal narrowing during angiography  (coronary or multi-slice computed tomography) | | Chronic ischemic heart disease (I25) | |
| Previous revascularization (percutaneous transluminal coronary angioplasty ± stent or coronary artery bypass graft >2 months prior to consent | | Procedure code [Coronary angiography (HA670), aortocoronary venous bypass graft angiography (HA680, HA681, HA682), percutaneous transluminal coronary angioplasty (M6551, M6552), percutaneous transcatheter placement of intracoronary stent (M6561, M6562, M6563, M6564), percutaneous transluminal coronary atherectomy (M6571, M6572), mechanical thrombectomy (M6633), percutaneous thrombus removal (M6634), vascular bypass operation (O1641, O1642, O1647, OA641, OA642, OA647)] | |
| The combination of revascularization in one major coronary artery and significant  stenosis (≥50% luminal narrowing) in another major coronary artery | | - | |
| Evidence of single-vessel coronary artery disease, ≥50% luminal narrowing during angiography (coronary or multi-slice computed tomography) not subsequently successfully revascularized | | - | |
| Unstable angina >2 months prior to consent with evidence of single- or multi-vessel coronary artery disease | | History of unstable angina (I20) >2 months prior to index date | |
| History of stroke (ischemic or hemorrhagic) >2 months prior to consent | | History of stroke (I60-I64 with procedure) >2 months prior to index date | |
| Occlusive peripheral artery disease documented by any of the following: | |  | |
| Limb angioplasty, stenting, or bypass surgery | | Percutaneous intravascular installation of metallic stent-others (M6603), vascular bypass operation, femoral (O0161 - O0171), percutaneous transluminal angioplasty-others (M6597) | |
| Limb or foot amputation due to circulatory insufficiency | | Lower extremity amputation (Z89.4-Z89.6) | |
| Evidence of significant peripheral artery stenosis (>50% on angiography, or >50% or hemodynamically significant via non-invasive methods ) in 1 limb | | - | |
| Ankle brachial index <0.9 in ≥1 ankle | | Atherosclerosis of native arteries of extremities with intermittent claudication (I70.22) | |
| **Exclusion criteria** |  |  |  |
| Uncontrolled hyperglycaemia with a glucose level >240 mg/dl (>13.3 mmol/L) after an overnight fast during placebo run-in and confirmed by a second measurement (not on the same day) | | - | |
| Indication of liver disease, defined by serum levels of either alanine aminotransferase (ALT), aspartate aminotransferase ALT or alkaline phosphatase above 3 x upper limit of normal (ULN) as determined at screening and/or run in. | | History of viral hepatitis (B15-B19, B25.1), liver disease (K70-K77), esophageal varices (I85), biliary tract disease (K83), disorder of gallbladder (K87)  Jaundice (R17), ascite (R18)  within 3 months from index date | |
| Planned cardiac surgery or angioplasty within 3 months | | - | |
| Impaired renal function, defined as Glomerular Filtration Rate <30 ml/min (severe renal impairment, Modification of Diet in Renal Disease formula) during screening or run in. | | History of CKD (Chronic Kidney Disease) stage 3 (N18.4), 4 (N18.5), 5(N18.6)  within 3 months from index date | |
| Bariatric surgery within the past two years and other gastrointestinal surgeries that induce chronic malabsorption | | Bariatric surgery (Q2630, Q2633 – Q2639, QA630, QA633 – QA638, QA643 – QA647) | |
| Blood dyscrasias or any disorders causing haemolysis or unstable Red Blood Cell (e.g. malaria, babesiosis, haemolytic anemia) | | Blood dyscrasias (D50-D77),  disorders of iron metabolism (E83.1), history of malaria (B50-54), and babesiosis (B60.0)  within 3 months from index date | |
| Medical history of cancer (except for basal cell carcinoma) and/or treatment for cancer within the last 5 years | | History of cancer (C00-C97)  within 5 years from index date | |
| Contraindications to background therapy according to the local label | | - | |
| Treatment with anti-obesity drugs (e.g. sibutramine, orlistat) 3 months prior to informed consent or any other treatment at the time of screening (i.e. surgery, aggressive diet regimen, etc.) leading to unstable body weight | | Anti-obesity drugs prescription (sibutramine, ATC code: A08AA10; orlistat, ATC code: A08AB01) within 3 months from index date | |
| Current treatment with systemic steroids at time of informed consent or change in dosage of thyroid hormones within 6 weeks prior to informed consent or any other uncontrolled endocrine disorder except type 2 diabetes mellitus | | Systemic steroids prescription  within 6 weeks from index date  or  change in dosage of thyroid hormones (ATC code: H03AA01-H03AA05) within 6 weeks from index date | |
| Pre-menopausal women (last menstruation <+ 1 year prior to informed consent) are nursing or pregnant | | Normal pregnancy (Z34.0, Z34.8, Z34.9), pregnancy-related disease (O00-O99) | |
| Alcohol or drug abuse within the 3 months prior to informed consent that would interfere with trial participation or any ongoing condition leading to a decreased compliance to study procedures or study drug intake | | History of alcohol (F10-F19, G62.1, I42.6, K70)  or drug abuse (F55)  within 3 months from index date | |
| Participation in another trial with an investigational drug within 30 days prior to informed consent | | - | |
| Any other clinical condition that would jeopardize patients safety while participating in this clinical trial | | - | |
| Acute coronary syndrome, stroke or transient ischemic attackwithin 2 months prior to informed consent | | History of acute coronary syndrome (I20, I21, or revascularization procedure), stroke (I60-66 with procedure), or transient ischemic attack (G45) within 2 months from index date | |

Anatomical Therapeutic Chemical, ATC

**Supplementary Table S4 Operational definitions of outcomes**

| **Outcome definition**  **using MedDRA PT terminology**  **in EMPA-REG outcome trial** | **Operational Definition**  **in EMPA-REG duplicate study** |
| --- | --- |
| **Nonfatal myocardial infarction excluding silent myocardial infarction** | Myocardial infarction (I21) with procedure code |
| **Hospitalization for unstable angina** | Unstable angina (I20) with hospitalization |
| **Coronary revascularization procedure** | Procedure code [Coronary angiography (HA670), aortocoronary venous bypass graft angiography (HA680, HA681, HA682), percutaneous transluminal coronary angioplasty (M6551, M6552), percutaneous transcatheter placement of intracoronary stent (M6561, M6562, M6563, M6564), percutaneous transluminal coronary atherectomy (M6571, M6572), mechanical thrombectomy (M6633), percutaneous thrombus removal (M6634), vascular bypass operation (O1641, O1642, O1647, OA641, OA642, OA647)] |
| **Nonfatal stroke** | Cerebrovascular diseases (I60-I64) with procedure code |
| **Transient ischemic attack** | Transient ischemic attack (G45) |
| **Hospitalization for heart failure** |  |
| Cardiac failure | Heart failure (I50) |
| Left ventricular failure | Left ventricular failure (I50.1) |
| Cardiac asthma | Left ventricular failure (I50.1) |
| Cardiogenic shock | Cardiogenic shock (R57.0) |
| Cor pulmonale | ~~-~~ |
| Pulmonary oedema | Pulmonary edema (J81) |
| Ejection fraction decreased | Systolic (congestive) heart failure (I50.2) |
| **Confirmed hypoglycemic adverse event** | Type 2 diabetes mellitus with hypoglycemia (E11.63), other specified diabetes mellitus with hypoglycemia (E13.64),  nondiabetic hypoglycemic coma (E15),  drug-induced hypoglycemia without coma (E16.0), other hypoglycemia (E16.1),  hypoglycemia, unspecified (E16.2) |
| **Event consistent with urinary tract infection** | |
| Urinary tract infection (fungal, bacterial including Escherichia infection) | Urinary tract infection, site not specified (N39.0) |
| Cystitis | Cystitis (N30) |
| Bacteriuria (including asymptomatic) | Abnormal findings on microbiological examination of urine (R82.7) |
| Pyelonephritis (acute, chronic) | Acute pyelonephritis (N10), Chronic tubulo-interstitial nephritis (N11), Tubulo-interstitial nephritis, not specified (N12) |
| Urosepsis | Renal and perinephric abscess (N15.1) with any of above codes |
| **Event consistent with genital infection** | |
| Balanitis candida | Candida balanitis(B37.41) |
| Balanoposthitis (including infective) | Balanoposthitis (N48.1) |
| Cellulitis of male external genital organ | Cellulitis of corpus cavernosum and penis (N48.21) |
| Epididymitis/Orchitis (including Scrotal abscess) | Epididymitis and orchitis (N45) |
| Penile infection | Other inflammatory disorders of penis (N48.2) |
| Phimosis | ~~-~~ |
| Prostate infection | Acute prostatitis (N41.0)  Chronic prostatitis (N41.1)  Other inflammatory diseases of prostate (N41.8)  Inflammatory disease of prostate, unspecified (N41.9) |
| Prostatic abscess | Abscess of prostate (N41.2) |
| Prostatitis | Inflammatory diseases of prostate (N41) |
| Vaginal cellulitis | - |
| Vaginal infection (bacterial, fungal)  or  Bacterial vaginosis | Acute vaginitis (N76.0)  Subacute and chronic vaginitis (N76.1)  Acute vulvitis (N76.2)  Subacute and chronic vulvitis (N76.3)  Other specified inflammation of vagina and vulva (N76.8)  Vaginitis, vulvitis and vulvovaginitis in diseases classified elsewhere (N77.1) |
| Vulval abscess | Vulval abscess (N76.4) |
| Vulvovaginal candidiasis | candida of vulva and vagina (B37.3) |
| Vulvovaginitis | vaginitis and vulvovaginitis (N77.1, N76.0-N76.3) |
| Genital candidiasis | Candidiasis of other urogenital sites (B37.4) |
| Urogenital infection fungal | candida of other urogenital sites (B37.48) |
| Perineal abscess | ~~-~~ |
| **Event consistent with volume depletion** | |
| Hypotension | Hypotension (I95.9) |
| Syncope | Syncope and collapse (R55) |
| Dehydration | Dehydration (E86.0), Volume depletion, unspecified (E86.8) |
| Orthostatic hypotension | Orthostatic hypotension (I95.1) |
| Blood pressure decreased | Nonspecific low blood-pressure reading (R03.1) |
| Hypovolaemia | Hypovolemia (E86.1) |
| **Acute kidney injury** | Acute kidney failure (N17) |
| **Diabetic ketoacidosis** | Diabetic ketoacidosis (E11.1, E13.1, E14.1) |
| **Thromboembolic event** | |
| Deep vein thrombosis, Venous thrombosis,and Venous occlusion | Other venous embolism and thrombosis (I82) |
| Pulmonary embolism | Pulmonary embolism (I26) |
| Thrombophlebitis | Phlebitis and thrombophlebitis (I80) |
| Retinal vein occlusion | Other retinal vein occlusions (H34.8) |
| Thrombophlebitis supreficial | Phlebitis and thrombophlebitis, superficial (I80.0) |
| Deep vein thrombosis postoperative | Complication of vein following a procedure (T81.7) |
| Mesenteric vein thrombosis | Vascular disorders of intestine (K55) |
| Post thrombotic syndrome | Postthrombotic syndrome (I87.0) |
| **Bone fracture** | |
| Rib fracture | Fracture of sternum ([S22.2](https://www.icd10data.com/ICD10CM/Codes/S00-T88/S20-S29/S22-/S22.2))  Fracture of rib ([S22.3](https://www.icd10data.com/ICD10CM/Codes/S00-T88/S20-S29/S22-/S22.3))  Multiple fractures of ribs (S22.4) |
| Foot fracture | Foot Fracture (S92) |
| Upper limb fracture, Humerus fracture | Fracture of upper end of humerus (S42.2)  Fracture of shaft of humerus (S42.3)  Fracture of lower end of humerus (S42.4) |
| Ankle fracture | Fracture of medial malleolus (S82.5)  Fracture of lateral malleolus (S82.6) |
| Pathological fracture | Pathological fracture, not elsewhere classified (M84.4) |
| Hip fracture | Fracture of sacrum (S32.1)  Fracture of ilium (S32.3)  Fracture of other and unspecified parts of lumbar spine and pelvis (S32.8)  Fracture of ischium (S32.82) |
| Radius fracture | Fracture of upper end of radius (S52.1)  Fracture of shaft of radius (S52.3)  Fracture of lower end of radius (S52.5) |
| Tooth fracture | Fracture of tooth (S02.5) |
| Hand fracture | Fracture of navicular [scaphoid] bone of hand (S62.0)  Fracture of other carpal bone(s) (S62.1)  Fracture of thumb (S62.5)  Fracture of other finger (S62.6)  Multiple fracture of fingers (S62.7) |
| Tibia fracture | Fracture of upper end of tibia (S82.1)  Fracture of shaft of tibia (S82.2)  Fracture of lower end of tibia (S82.3) |
| Facial bones fracture | Fracture of skull and facial bones (S02, excluding S02.5, S02.6) |
| Wrist fracture | Fracture of first metacarpal bone (S62.2)  Fracture of other metacarpal bone (S62.3)  Multiple fracture of metacarpal bones (S62.4)  Fracture of other and unspecified parts of wrist and hand (S62.8) |
| Spinal compression fracture | Collapsed vertebra (M48.5) |
| Femoral neck fracture | Fracture of head and neck of femur (S72.0) |
| Femur fracture | Fracture of femur (S72) |
| Fibula fracture | Fracture of fibula alone (S82.4) |
| Pelvic fracture | Fracture of other and unspecified parts of lumbar spine and pelvis (S32.8) |
| Acetabulum fracture | Fracture of acetabulum ([S32.4](https://www.icd10data.com/ICD10CM/Codes/S00-T88/S30-S39/S32/S32.4-)) |
| Lumbar vertebral fracture | Fracture of lumbar vertebra (S32.0) |
| Osteoporotic fracture | Osteoporosis with pathological fracture (M80) |
| Patella fracture | Fracture of patella (S82.0) |
| Clavicle fracture | Fracture of clavicle (S42.0) |
| Forearm fracture | Multiple fracture of forearm (S52.7)  Fracture of other parts of forearm (S52.8)  Unspecified fracture of forearm (S52.9) |
| Jaw fracture | Fracture of mandible (S02.6) |
| Ulna fracture | Fracture of upper end of ulna (S52.0)  Fracture of shaft of ulna (S52.2)  Fracture of shafts of both ulna and radius (S52.4)  Fracture of lower end of both ulna and radius (S52.6) |
| Fractured coccyx | Fracture of coccyx ([S32.2](https://www.icd10data.com/ICD10CM/Codes/S00-T88/S30-S39/S32/S32.2-)) |
| Lower limb fracture | Other fractures of lower leg (S82.8)  Unspecified fracture of lower leg (S82.9) |
| Periprosthetic fracture | Fracture of bone following insertion of orthopaedic implant, joint prosthesis, or bone plate (M96.6) |
| Pubis fracture | Fracture of pubis ([S32.5](https://www.icd10data.com/ICD10CM/Codes/S00-T88/S30-S39/S32/S32.5-/S32.5)) |
| Avulsion fracture | ~~-~~ |
| Cervical vertebral fracture | Fracture of cervical vertebra and other parts of neck (S12) |
| Scapula fracture | Fracture of scapula (S42.1) |
| Skull fractured base | Fracture of base of skull (S02.1) |
| Thoracic vertebral fracture | Fracture of thoracic vertebra (S22.0) |
| Traumatic fracture | ~~-~~ |
| Open fracture | Included in each fracture code |

**Supplementary Table S5 Baseline characteristics of cohort for hospitalization for heart failure**

|  | **Pre-Match** | | **STD** | **Post-Match** | | | **STD** |
| --- | --- | --- | --- | --- | --- | --- | --- |
|  | **Sitagliptin**  **N= 34,541** | **Empagliflozin**  **N= 10,867** |  | **Sitagliptin**  **N= 10,779** | **Empagliflozin**  **N= 10,779** | |  |
| Sex | 19116 (55.4) | 6389 (58.8) | -0.03 | 6308 (58.5) | 6322 (58.7) | | 0.011 |
| Age | 60.1 ± 11.3 | 55.3 ± 10.9 | -0.43 | 55.5 ± 11.2 | 55.5 ± 10.8 | | -0.002 |
| Insurance type | | | | | | | |
| Normal | 32336 (93.7) | 10301 (94.8) | 0.05 | 10214 (94.8) | | 10216 (94.8) | 0.010 |
| Medicaid | 2007 (5.8) | 535 (4.9) |  | 539 (5.0) | 532 (4.9) | |  |
| No charge | 171 (0.5) | 31 (0.3) |  | 26 (0.2) | 31 (0.3) | |  |
| Number of  Inpatient visit | 0.7 ± 2.1 | 0.4 ± 1.1 | -0.20 | 0.4 ± 1.1 | 0.4 ± 1.1 | | 0.004 |
| Number of  outpatient visit | 28.0 ± 27.5 | 25.4 ± 23.6 | -0.10 | 25.6 ± 24.4 | 25.5 ± 23.7 | | -0.004 |
| Index year | | | | | | | |
| 2016 | 10000 (29.0) | 2148 (19.8) | 0.22 | 2171 (20.1) | 2147 (19.9) | | 0.010 |
| 2017 | 17111 (49.6) | 5869 (54.0) |  | 5840 (54.2) | 5819 (54.0) | |  |
| 2018 | 7403 (21.5) | 2850 (26.2) |  | 2768 (25.7) | 2813 (26.1) | |  |
| Charlson comorbidity index | | | | | | | |
| 0 | 1519 (4.4) | 471 (4.3) | 0.04 | 479 (4.4) | 468 (4.3) | | 0.019 |
| 1 | 3356 (9.7) | 1143 (10.5) |  | 1122 (10.4) | 1131 (10.5) | |  |
| 2 | 4870 (14.1) | 1611 (14.8) |  | 1529 (14.2) | 1599 (14.8) | |  |
| 3 | 24769 (71.8) | 7642 (70.3) |  | 7649 (71.0) | 7581 (70.3) | |  |
| CV risk factor | | | | | | | |
| CAD | 30402 (88.1) | 10020 (92.2) | 0.14 | 9876 (91.6) | 9937 (92.2) | | 0.021 |
| Multi vessel CAD | 14602 (42.3) | 5512 (50.7) | 0.17 | 5305 (49.2) | 5440 (50.5) | | 0.025 |
| MI | 1493 (4.3) | 714 (6.6) | 0.10 | 655 (6.1) | 687 (6.4) | | 0.012 |
| CABG | 6398 (18.5) | 2777 (25.6) | 0.17 | 2651 (24.6) | 2727 (25.3) | | 0.016 |
| Stroke | 4805 (13.9) | 953 (8.8) | -0.16 | 932 (8.7) | 950 (8.8) | | 0.006 |
| PAD | 2241 (6.5) | 624 (5.7) | -0.03 | 633 (5.9) | 620 (5.8) | | -0.005 |
| DM circulation | 4529 (13.1) | 1639 (15.1) | 0.06 | 1678 (15.6) | 1618 (15.0) | | -0.016 |
| DM foot | 2 (0.0) | 3 (0.0) | 0.02 | 2 (0.0) | 3 (0.0) | | 0.006 |
| DM nephropathy | 2176 (6.3) | 955 (8.8) | 0.09 | 912 (8.5) | 929 (8.6) | | 0.006 |
| DM neuropathy | 4913 (14.2) | 1476 (13.6) | -0.02 | 1522 (14.1) | 1468 (13.6) | | -0.015 |
| DM other  Complications | 25483 (73.8) | 7743 (71.3) | -0.06 | 7685 (71.3) | 7697 (71.4) | | 0.003 |
| Hyperglycemia | 615 (1.8) | 147 (1.4) | -0.03 | 146 (1.4) | 144 (1.3) | | -0.002 |
| Comorbidities | | | | | | | |
| Hypertension | 26809 (77.7) | 8503 (78.3) | 0.01 | 8474 (78.6) | 8430 (78.2) | | -0.010 |
| Edema | 3044 (8.8) | 914 (8.4) | -0.01 | 937 (8.7) | 902 (8.4) | | -0.012 |
| Kidney stone | 549 (1.6) | 156 (1.4) | -0.01 | 139 (1.3) | 155 (1.4) | | 0.013 |
| Osteoarthritis | 12265 (35.5) | 3346 (30.8) | -0.10 | 3395 (31.5) | 3337 (31.0) | | -0.012 |
| Other arthritis | 8470 (24.5) | 2347 (21.6) | -0.07 | 2397 (22.2) | 2337 (21.7) | | -0.013 |
| PUD | 8748 (25.4) | 2626 (24.2) | -0.03 | 2608 (24.2) | 2610 (24.2) | | 0.000 |
| Pancreatitis | 308 (0.9) | 91 (0.8) | -0.01 | 83 (0.8) | 91 (0.8) | | 0.008 |
| UC | 55 (0.2) | 12 (0.1) | -0.01 | 9 (0.1) | 12 (0.1) | | 0.009 |
| Crohn | 13 (0.0) | 4 (0.0) | 0.00 | 5 (0.1) | 4 (0.0) | | -0.005 |
| Asthma | 4889 (14.2) | 1446 (13.3) | -0.03 | 1415 (13.1) | 1436 (13.3) | | 0.006 |
| COPD | 1122 (3.3) | 241 (2.2) | -0.06 | 234 (2.2) | 241 (2.2) | | 0.004 |
| Bladder stone | 28 (0.1) | 5 (0.1) | -0.01 | 3 (0.0) | 5 (0.1) | | 0.010 |
| Dementia | 5459 (15.8) | 1044 (9.6) | -0.19 | 1025 (9.5) | 1043 (9.7) | | 0.006 |
| Electrolyte  Imbalance | 1973 (5.7) | 514 (4.7) | -0.04 | 515 (4.8) | 511 (4.7) | | -0.002 |
| Glaucoma  /Cataract | 9838 (28.5) | 2939 (27.1) | -0.03 | 2947 (27.3) | 2919 (27.1) | | -0.006 |
| HONK | 262 (0.8) | 61 (0.6) | -0.02 | 43 (0.4) | 61 (0.6) | | 0.024 |
| HTN  nephropathy | 149 (0.4) | 50 (0.5) | 0.00 | 44 (0.4) | 49 (0.5) | | 0.007 |
| Hyperthyroid  disease | 632 (1.8) | 208 (1.9) | 0.01 | 217 (2.0) | 207 (1.9) | | -0.007 |
| Hypothyroid  disease | 1640 (4.8) | 548 (5.0) | 0.01 | 555 (5.2) | 544 (5.1) | | -0.005 |
| Osteomyelitis | 254 (0.7) | 61 (0.6) | -0.02 | 62 (0.6) | 61 (0.6) | | -0.001 |
| Pneumonia | 2465 (7.1) | 662 (6.1) | -0.04 | 668 (6.2) | 660 (6.1) | | -0.003 |
| Skin infection | 1338 (3.9) | 419 (3.9) | 0.00 | 402 (3.7) | 417 (3.9) | | 0.007 |
| Glucose-lowering therapy | | | | | | | |
| Metformin | 24258 (70.3) | 7902 (72.7) | 0.05 | 7876 (73.1) | 7837 (72.7) | | -0.008 |
| Insulins | 5569 (16.1) | 1890 (17.4) | 0.03 | 1823 (16.9) | 1853 (17.2) | | 0.007 |
| SUs | 15829 (45.9) | 5142 (47.3) | 0.03 | 5146 (47.7) | 5097 (47.3) | | -0.009 |
| Glitazones | 3110 (9.0) | 1256 (11.6) | 0.08 | 1285 (11.9) | 1242 (11.5) | | -0.012 |
| GLP-1 agonists | 103 (0.3) | 76 (0.7) | 0.06 | 72 (0.7) | 69 (0.6) | | -0.004 |
| AGIs | 1425 (4.1) | 333 (3.1) | -0.06 | 354 (3.3) | 332 (3.1) | | -0.012 |
| Meglitinides | 222 (0.6) | 79 (0.7) | 0.01 | 72 (0.7) | 78 (0.7) | | 0.007 |
| Co-medications | | | | | | | |
| Anticoagulants | 1259 (3.7) | 405 (3.7) | 0.00 | 431 (4.0) | 401 (3.7) | | -0.014 |
| Antiplatelets | 22721 (65.8) | 7608 (70.0) | 0.09 | 7548 (70.0) | 7536 (69.9) | | -0.002 |
| Heparins | 1129 (3.3) | 306 (2.8) | -0.03 | 292 (2.7) | 305 (2.8) | | 0.007 |
| Thrombolytics | 50 (0.1) | 8 (0.1) | -0.02 | 3 (0.0) | 8 (0.1) | | 0.021 |
| Statins | 24127 (69.9) | 8756 (80.6) | 0.25 | 8687 (80.6) | 8668 (80.4) | | -0.004 |
| Other lipid  Lowerings | 3686 (10.7) | 1554 (14.3) | 0.11 | 1601 (14.9) | 1518 (14.1) | | -0.022 |
| Nitrates | 5419 (15.7) | 2110 (19.4) | 0.10 | 2002 (18.6) | 2077 (19.3) | | 0.018 |
| Digoxin | 4630 (13.4) | 1835 (16.9) | 0.10 | 1733 (16.1) | 1802 (16.7) | | 0.017 |
| ACEIs | 1769 (5.1) | 770 (7.1) | 0.08 | 697 (6.5) | 749 (7.0) | | 0.019 |
| ARBs | 20054 (58.1) | 6761 (62.2) | 0.08 | 6748 (62.6) | 6693 (62.1) | | -0.011 |
| Entresto | 1 (0.0) | 4 (0.0) | 0.02 | 1 (0.0) | 1 (0.0) | | 0.000 |
| Other  Anti HTNs | 22197 (64.3) | 7430 (68.4) | 0.09 | 7301 (67.7) | 7352 (68.2) | | 0.010 |
| Loop diuretics | 3222 (9.3) | 965 (8.9) | -0.02 | 923 (8.6) | 944 (8.8) | | 0.007 |
| Other diuretics | 8987 (26.0) | 2815 (25.9) | 0.00 | 2820 (26.2) | 2783 (25.8) | | -0.008 |
| Antianxieties | 13859 (40.2) | 3872 (35.6) | -0.09 | 3837 (35.6) | 3858 (35.8) | | 0.004 |
| Antipsychotics | 1537 (4.5) | 262 (2.4) | -0.11 | 263 (2.4) | 262 (2.4) | | -0.001 |
| Antidepressants | 6092 (17.7) | 1638 (15.1) | -0.07 | 1655 (15.4) | 1628 (15.1) | | -0.007 |
| Dementia | 5459 (15.8) | 1044 (9.6) | -0.19 | 1025 (9.5) | 1043 (9.7) | | 0.006 |
| Antiparkinsons | 1048 (3.0) | 163 (1.5) | -0.10 | 174 (1.6) | 163 (1.5) | | -0.008 |
| Anticonvulsants | 856 (2.5) | 170 (1.6) | -0.07 | 171 (1.6) | 170 (1.6) | | -0.001 |
| NSAIDs | 26296 (76.2) | 8236 (75.8) | -0.01 | 8158 (75.7) | 8177 (75.9) | | 0.004 |
| Bisphospho-nates | 1601 (4.6) | 341 (3.1) | -0.08 | 339 (3.2) | 341 (3.2) | | 0.001 |
| Opioids | 15169 (44.0) | 4389 (40.4) | -0.07 | 4349 (40.4) | 4364 (40.5) | | 0.003 |

Values are represented as mean ± standard deviation or number (%); ACEis, angiotensin-converting enzyme inhibitors; AGIs, α-glucosidase Inhibitors; ARBs, angiotensin II receptor blockers; CABG, coronary artery bypass graft; CAD, coronary artery disease; COPD, chronic obstructive pulmonary disease; CV, cardiovascular; DM, diabetes mellitus; HONK, hyperglycaemic hyperosmolar nonketotic coma; HTN, hypertensive; MI, myocardial infarction; NSAIDs, non-steroidal anti-inflammatory drugs; PAD, peripheral artery disease; PUD, peptic ulcer disease; STD, standardized difference; SUs, sulfonylureas; UC, ulcerative colitis;

**Supplementary Table S6 Baseline characteristics of cohort for hypoglycemic adverse event**

|  | **Pre-Match** | | **STD** | **Post-Match** | | | **STD** |
| --- | --- | --- | --- | --- | --- | --- | --- |
|  | **Sitagliptin**  **N= 35,240** | **Empagliflozin**  **N= 11,343** |  | **Sitagliptin**  **N= 11,216** | **Empagliflozin**  **N= 11,216** | |  |
| Sex | 19602 (55.6) | 6751 (59.5) | -0.04 | 6608 (58.9) | 6660 (59.4) | | -0.001 |
| Age | 60.1 ± 11.3 | 55.3 ± 10.9 | -0.43 | 55.5 ± 11.3 | 55.5 ± 10.8 | | -0.002 |
| Insurance type | | | | | | | |
| Normal | 33017 (93.7) | 10762 (94.9) | 0.05 | 10636 (94.8) | | 10637 (94.8) | 0.012 |
| Medicaid | 2041 (5.8) | 546 (4.8) |  | 552 (4.9) | 544 (4.9) | |  |
| No charge | 182 (0.5) | 35 (0.3) |  | 28 (0.3) | 35 (0.3) | |  |
| Number of  Inpatient visit | 0.8 ± 2.2 | 0.5 ± 1.2 | -0.20 | 0.5 ± 1.1 | 0.4 ± 1.2 | | -0.006 |
| Number of  outpatient visit | 27.8 ± 27.1 | 25.3 ± 23.6 | -0.10 | 25.6 ± 24.1 | 25.4 ± 23.6 | | -0.011 |
| Index year | | | | | | | |
| 2016 | 10131 (28.8) | 2189 (19.3) | 0.23 | 2186 (19.5) | 2186 (19.5) | | 0.001 |
| 2017 | 17468 (49.6) | 6129 (54.0) |  | 6058 (54.0) | 6063 (54.1) | |  |
| 2018 | 7641 (21.7) | 3025 (26.7) |  | 2972 (26.5) | 2967 (26.5) | |  |
| Charlson comorbidity index | | | | | | | |
| 0 | 1512 (4.3) | 469 (4.1) | 0.03 | 422 (3.8) | 466 (4.2) | | 0.022 |
| 1 | 3284 (9.3) | 1117 (9.9) |  | 1104 (9.8) | 1106 (9.9) | |  |
| 2 | 4779 (13.6) | 1612 (14.2) |  | 1570 (14.0) | 1598 (14.3) | |  |
| 3 | 25665 (72.8) | 8145 (71.8) |  | 8120 (72.4) | 8046 (71.7) | |  |
| CV risk factor | | | | | | | |
| CAD | 31197 (88.5) | 10511 (92.7) | 0.14 | 10320 (92.0) | 10386 (92.6) | | 0.022 |
| Multi vessel CAD | 15500 (44.0) | 5997 (52.9) | 0.18 | 5813 (51.8) | 5891 (52.5) | | 0.014 |
| MI | 1832 (5.2) | 878 (7.7) | 0.10 | 870 (7.8) | 847 (7.6) | | -0.008 |
| CABG | 7338 (20.8) | 3246 (28.6) | 0.18 | 3195 (28.5) | 3171 (28.3) | | -0.005 |
| Stroke | 4947 (14.0) | 1003 (8.8) | -0.16 | 1051 (9.4) | 1001 (8.9) | | -0.016 |
| PAD | 2229 (6.3) | 638 (5.6) | -0.03 | 664 (5.9) | 635 (5.7) | | -0.011 |
| DM circulation | 4621 (13.1) | 1725 (15.2) | 0.06 | 1670 (14.9) | 1695 (15.1) | | 0.006 |
| DM foot | 3 (0.0) | 4 (0.0) | 0.02 | 3 (0.0) | 1 (0.0) | | -0.013 |
| DM nephropathy | 2188 (6.2) | 972 (8.6) | 0.09 | 927 (8.3) | 950 (8.5) | | 0.007 |
| DM neuropathy | 4881 (13.9) | 1519 (13.4) | -0.01 | 1563 (13.9) | 1504 (13.4) | | -0.015 |
| DM other  Complications | 26049 (73.9) | 8092 (71.3) | -0.06 | 8052 (71.8) | 8020 (71.5) | | -0.006 |
| Hyperglycemia | 268 (0.8) | 84 (0.7) | 0.00 | 83 (0.7) | 82 (0.7) | | -0.001 |
| Comorbidities | | | | | | | |
| Hypertension | 27528 (78.1) | 8932 (78.7) | 0.02 | 8805 (78.5) | 8823 (78.7) | | 0.004 |
| Edema | 3255 (9.2) | 1013 (8.9) | -0.01 | 1027 (9.2) | 995 (8.9) | | -0.010 |
| Kidney stone | 554 (1.6) | 161 (1.4) | -0.01 | 175 (1.6) | 159 (1.4) | | -0.012 |
| Osteoarthritis | 12434 (35.3) | 3431 (30.3) | -0.11 | 3448 (30.7) | 3414 (30.4) | | -0.007 |
| Other arthritis | 8599 (24.4) | 2475 (21.8) | -0.06 | 2444 (21.8) | 2452 (21.9) | | 0.002 |
| PUD | 8881 (25.2) | 2717 (24.0) | -0.03 | 2680 (23.9) | 2695 (24.0) | | 0.003 |
| Pancreatitis | 314 (0.9) | 95 (0.8) | -0.01 | 102 (0.9) | 94 (0.8) | | -0.008 |
| UC | 56 (0.2) | 12 (0.1) | -0.01 | 10 (0.1) | 12 (0.1) | | 0.006 |
| Crohn | 14 (0.0) | 5 (0.0) | 0.00 | 2 (0.0) | 5 (0.0) | | 0.015 |
| Asthma | 5145 (14.6) | 1562 (13.8) | -0.02 | 1526 (13.6) | 1541 (13.7) | | 0.004 |
| COPD | 1264 (3.6) | 281 (2.5) | -0.06 | 271 (2.4) | 280 (2.5) | | 0.005 |
| Bladder stone | 27 (0.1) | 4 (0.0) | -0.02 | 3 (0.0) | 4 (0.0) | | 0.005 |
| Dementia | 5521 (15.7) | 1086 (9.6) | -0.18 | 1136 (10.1) | 1083 (9.7) | | -0.016 |
| Electrolyte  Imbalance | 2081 (5.9) | 566 (5.0) | -0.04 | 578 (5.2) | 555 (5.0) | | -0.009 |
| Glaucoma  /Cataract | 9992 (28.4) | 3050 (26.9) | -0.03 | 3069 (27.4) | 3027 (27.0) | | -0.008 |
| HONK | 257 (0.7) | 59 (0.5) | -0.03 | 65 (0.6) | 58 (0.5) | | -0.009 |
| HTN  nephropathy | 152 (0.4) | 50 (0.4) | 0.00 | 50 (0.5) | 50 (0.5) | | 0.000 |
| Hyperthyroid  disease | 654 (1.9) | 214 (1.9) | 0.00 | 228 (2.0) | 211 (1.9) | | -0.011 |
| Hypothyroid  disease | 1692 (4.8) | 578 (5.1) | 0.01 | 544 (4.9) | 571 (5.1) | | 0.011 |
| Osteomyelitis | 265 (0.8) | 64 (0.6) | -0.02 | 77 (0.7) | 64 (0.6) | | -0.015 |
| Pneumonia | 2667 (7.6) | 741 (6.5) | -0.04 | 753 (6.7) | 733 (6.5) | | -0.007 |
| Skin infection | 1357 (3.9) | 448 (4.0) | 0.01 | 444 (4.0) | 444 (4.0) | | 0.000 |
| Glucose-lowering therapy | | | | | | | |
| Metformin | 24676 (70.0) | 8222 (72.5) | 0.05 | 8136 (72.5) | 8128 (72.5) | | -0.002 |
| Insulins | 5693 (16.2) | 1997 (17.6) | 0.04 | 1933 (17.2) | 1949 (17.4) | | 0.004 |
| SUs | 15935 (45.2) | 5309 (46.8) | 0.03 | 5317 (47.4) | 5236 (46.7) | | -0.015 |
| Glitazones | 3104 (8.8) | 1275 (11.2) | 0.08 | 1307 (11.7) | 1252 (11.2) | | -0.015 |
| GLP-1 agonists | 107 (0.3) | 77 (0.7) | 0.05 | 71 (0.6) | 69 (0.6) | | -0.002 |
| AGIs | 1417 (4.0) | 344 (3.0) | -0.05 | 346 (3.1) | 342 (3.1) | | -0.002 |
| Meglitinides | 223 (0.6) | 82 (0.7) | 0.01 | 82 (0.7) | 79 (0.7) | | -0.003 |
| Co-medications | | | | | | | |
| Anticoagulants | 1563 (4.4) | 542 (4.8) | 0.02 | 547 (4.9) | 531 (4.7) | | -0.007 |
| Antiplatelets | 23363 (66.3) | 7991 (70.5) | 0.09 | 7898 (70.4) | 7897 (70.4) | | 0.000 |
| Heparins | 1217 (3.5) | 340 (3.0) | -0.03 | 373 (3.3) | 338 (3.0) | | -0.018 |
| Thrombolytics | 52 (0.2) | 10 (0.1) | -0.02 | 8 (0.1) | 10 (0.1) | | 0.006 |
| Statins | 24914 (70.7) | 9178 (80.9) | 0.24 | 9035 (80.6) | 9056 (80.7) | | 0.005 |
| Other lipid  Lowerings | 3753 (10.7) | 1622 (14.3) | 0.11 | 1605 (14.3) | 1585 (14.1) | | -0.005 |
| Nitrates | 5980 (17.0) | 2371 (20.9) | 0.10 | 2365 (21.1) | 2320 (20.7) | | -0.010 |
| Digoxin | 5151 (14.6) | 2072 (18.3) | 0.10 | 2061 (18.4) | 2025 (18.1) | | -0.008 |
| ACEIs | 2028 (5.8) | 930 (8.2) | 0.10 | 904 (8.1) | 893 (8.0) | | -0.004 |
| ARBs | 20506 (58.2) | 7077 (62.4) | 0.09 | 7016 (62.6) | 6984 (62.3) | | -0.006 |
| Entresto | 6 (0.0) | 17 (0.2) | 0.05 | 6 (0.1) | 8 (0.1) | | 0.007 |
| Other  Anti HTNs | 23030 (65.4) | 7893 (69.6) | 0.09 | 7779 (69.4) | 7779 (69.4) | | 0.000 |
| Loop diuretics | 3960 (11.2) | 1290 (11.4) | 0.00 | 1276 (11.4) | 1241 (11.1) | | -0.010 |
| Other diuretics | 9537 (27.1) | 3113 (27.4) | 0.01 | 3067 (27.3) | 3050 (27.2) | | -0.003 |
| Antianxieties | 14193 (40.3) | 4060 (35.8) | -0.09 | 4117 (36.7) | 4024 (35.9) | | -0.017 |
| Antipsychotics | 1614 (4.6) | 285 (2.5) | -0.11 | 268 (2.4) | 285 (2.5) | | 0.010 |
| Antidepressants | 6202 (17.6) | 1686 (14.9) | -0.07 | 1688 (15.1) | 1672 (14.9) | | -0.004 |
| Dementia | 5521 (15.7) | 1086 (9.6) | -0.18 | 1136 (10.1) | 1083 (9.7) | | -0.016 |
| Antiparkinsons | 1035 (2.9) | 169 (1.5) | -0.10 | 171 (1.5) | 169 (1.5) | | -0.002 |
| Anticonvulsants | 875 (2.5) | 177 (1.6) | -0.07 | 197 (1.8) | 177 (1.6) | | -0.014 |
| NSAIDs | 26783 (76.0) | 8538 (75.3) | -0.02 | 8525 (76.0) | 8449 (75.3) | | -0.016 |
| Bisphospho-nates | 1636 (4.6) | 352 (3.1) | -0.08 | 366 (3.3) | 351 (3.1) | | -0.008 |
| Opioids | 15515 (44.0) | 4608 (40.6) | -0.07 | 4558 (40.6) | 4558 (40.6) | | 0.000 |

Values are represented as mean ± standard deviation or number (%); ACEis, angiotensin-converting enzyme inhibitors; AGIs, α-glucosidase Inhibitors; ARBs, angiotensin II receptor blockers; CABG, coronary artery bypass graft; CAD, coronary artery disease; COPD, chronic obstructive pulmonary disease; CV, cardiovascular; DM, diabetes mellitus; HONK, hyperglycaemic hyperosmolar nonketotic coma; HTN, hypertensive; MI, myocardial infarction; NSAIDs, non-steroidal anti-inflammatory drugs; PAD, peripheral artery disease; PUD, peptic ulcer disease; STD, standardized difference; SUs, sulfonylureas; UC, ulcerative colitis;

**Supplementary Table S7 Baseline characteristics of cohort for urinary tract infections**

|  | **Pre-Match** | | **STD** | **Post-Match** | | | **STD** |
| --- | --- | --- | --- | --- | --- | --- | --- |
|  | **Sitagliptin**  **N= 25,547** | **Empagliflozin**  **N= 8,358** |  | **Sitagliptin**  **N= 8,223** | **Empagliflozin**  **N= 8,223** | |  |
| Sex | 16371 (64.1) | 5713 (68.4) | -0.04 | 5574 (67.8) | 5600 (68.1) | | -0.008 |
| Age | 59.5 ± 11.3 | 54.8 ± 10.9 | -0.43 | 55.1 ± 11.1 | 55.0 ± 10.7 | | -0.003 |
| Insurance type | | | | | | | |
| Normal | 24090 (94.3) | 7971 (95.4) | 0.05 | 7836 (95.3) | | 7839 (95.3) | 0.003 |
| Medicaid | 1315 (5.2) | 361 (4.3) |  | 362 (4.4) | 358 (4.4) | |  |
| No charge | 142 (0.6) | 26 (0.3) |  | 25 (0.3) | 26 (0.3) | |  |
| Number of  Inpatient visit | 0.7 ± 1.9 | 0.4 ± 1.0 | -0.18 | 0.4 ± 1.0 | 0.4 ± 1.0 | | -0.008 |
| Number of  outpatient visit | 25.4 ± 25.5 | 22.7 ± 21.4 | -0.11 | 22.9 ± 21.4 | 22.8 ± 21.5 | | -0.003 |
| Index year | | | | | | | |
| 2016 | 7954 (31.1) | 1704 (20.4) | 0.26 | 1712 (20.8) | 1701 (20.7) | | 0.005 |
| 2017 | 12562 (49.2) | 4538 (54.3) |  | 4472 (54.4) | 4468 (54.3) | |  |
| 2018 | 5031 (19.7) | 2116 (25.3) |  | 2039 (24.8) | 2054 (25.0) | |  |
| Charlson comorbidity index | | | | | | | |
| 0 | 1306 (5.1) | 408 (4.9) | 0.02 | 378 (4.6) | 404 (4.9) | | 0.015 |
| 1 | 2703 (10.6) | 929 (11.1) |  | 924 (11.2) | 918 (11.2) | |  |
| 2 | 3758 (14.7) | 1253 (15.0) |  | 1241 (15.1) | 1239 (15.1) | |  |
| 3 | 17780 (69.6) | 5768 (69.0) |  | 5680 (69.1) | 5662 (68.9) | |  |
| CV risk factor | | | | | | | |
| CAD | 22574 (88.4) | 7730 (92.5) | 0.14 | 7600 (92.4) | 7600 (92.4) | | 0.000 |
| Multi vessel CAD | 11298 (44.2) | 4589 (54.9) | 0.21 | 4419 (53.7) | 4478 (54.5) | | 0.014 |
| MI | 1399 (5.5) | 719 (8.6) | 0.12 | 683 (8.3) | 678 (8.3) | | -0.002 |
| CABG | 5424 (21.2) | 2542 (30.4) | 0.21 | 2457 (29.9) | 2456 (29.9) | | 0.000 |
| Stroke | 3498 (13.7) | 727 (8.7) | -0.16 | 729 (8.9) | 722 (8.8) | | -0.003 |
| PAD | 1631 (6.4) | 460 (5.5) | -0.04 | 474 (5.8) | 456 (5.6) | | -0.010 |
| DM circulation | 3325 (13.0) | 1244 (14.9) | 0.05 | 1245 (15.1) | 1216 (14.8) | | -0.010 |
| DM foot | 0 (0.0) | 3 (0.0) | 0.03 | 0 (0.0) | 1 (0.0) | | 0.016 |
| DM nephropathy | 1542 (6.0) | 711 (8.5) | 0.10 | 639 (7.8) | 684 (8.3) | | 0.020 |
| DM neuropathy | 3405 (13.3) | 1049 (12.6) | -0.02 | 1092 (13.3) | 1036 (12.6) | | -0.020 |
| DM other  Complications | 18483 (72.4) | 5888 (70.5) | -0.04 | 5794 (70.5) | 5811 (70.7) | | 0.005 |
| Hyperglycemia | 422 (1.7) | 95 (1.1) | -0.04 | 92 (1.1) | 94 (1.1) | | 0.002 |
| Comorbidities | | | | | | | |
| Hypertension | 19761 (77.4) | 6581 (78.7) | 0.03 | 6447 (78.4) | 6466 (78.6) | | 0.006 |
| Edema | 2078 (8.1) | 626 (7.5) | -0.02 | 636 (7.7) | 611 (7.4) | | -0.012 |
| Kidney stone | 248 (1.0) | 75 (0.9) | -0.01 | 74 (0.9) | 74 (0.9) | | 0.000 |
| Osteoarthritis | 8109 (31.7) | 2217 (26.5) | -0.12 | 2242 (27.3) | 2206 (26.8) | | -0.010 |
| Other arthritis | 5680 (22.2) | 1629 (19.5) | -0.07 | 1638 (19.9) | 1602 (19.5) | | -0.011 |
| PUD | 6010 (23.5) | 1795 (21.5) | -0.05 | 1788 (21.7) | 1775 (21.6) | | -0.004 |
| Pancreatitis | 193 (0.8) | 61 (0.7) | 0.00 | 62 (0.8) | 59 (0.7) | | -0.004 |
| UC | 44 (0.2) | 7 (0.1) | -0.02 | 7 (0.1) | 7 (0.1) | | 0.000 |
| Crohn | 10 (0.0) | 3 (0.0) | 0.00 | 6 (0.1) | 3 (0.0) | | -0.016 |
| Asthma | 3371 (13.2) | 1038 (12.4) | -0.02 | 1063 (12.9) | 1020 (12.4) | | -0.016 |
| COPD | 850 (3.3) | 182 (2.2) | -0.07 | 187 (2.3) | 182 (2.2) | | -0.004 |
| Bladder stone | 9 (0.0) | 2 (0.0) | -0.01 | 3 (0.0) | 2 (0.0) | | -0.007 |
| Dementia | 3515 (13.8) | 654 (7.8) | -0.19 | 628 (7.6) | 652 (7.9) | | 0.011 |
| Electrolyte  Imbalance | 1308 (5.1) | 378 (4.5) | -0.03 | 374 (4.6) | 369 (4.5) | | -0.003 |
| Glaucoma  /Cataract | 6792 (26.6) | 2117 (25.3) | -0.03 | 2110 (25.7) | 2093 (25.5) | | -0.005 |
| HONK | 196 (0.8) | 39 (0.5) | -0.04 | 37 (0.5) | 38 (0.5) | | 0.002 |
| HTN  nephropathy | 106 (0.4) | 36 (0.4) | 0.00 | 33 (0.4) | 34 (0.4) | | 0.002 |
| Hyperthyroid  disease | 425 (1.7) | 150 (1.8) | 0.01 | 148 (1.8) | 148 (1.8) | | 0.000 |
| Hypothyroid  disease | 1080 (4.2) | 353 (4.2) | 0.00 | 359 (4.4) | 343 (4.2) | | -0.010 |
| Osteomyelitis | 168 (0.7) | 45 (0.5) | -0.02 | 46 (0.6) | 45 (0.6) | | -0.002 |
| Pneumonia | 1721 (6.7) | 473 (5.7) | -0.04 | 496 (6.0) | 464 (5.6) | | -0.017 |
| Skin infection | 928 (3.6) | 301 (3.6) | 0.00 | 311 (3.8) | 299 (3.6) | | -0.008 |
| Glucose-lowering therapy | | | | | | | |
| Metformin | 17811 (69.7) | 6048 (72.4) | 0.06 | 5918 (72.0) | 5951 (72.4) | | 0.009 |
| Insulins | 3928 (15.4) | 1421 (17.0) | 0.04 | 1396 (17.0) | 1373 (16.7) | | -0.008 |
| SUs | 11568 (45.3) | 3892 (46.6) | 0.03 | 3863 (47.0) | 3830 (46.6) | | -0.008 |
| Glitazones | 2223 (8.7) | 928 (11.1) | 0.08 | 924 (11.2) | 902 (11.0) | | -0.009 |
| GLP-1 agonists | 66 (0.3) | 53 (0.6) | 0.06 | 45 (0.6) | 42 (0.5) | | -0.005 |
| AGIs | 1023 (4.0) | 261 (3.1) | -0.05 | 260 (3.2) | 256 (3.1) | | -0.003 |
| Meglitinides | 171 (0.7) | 61 (0.7) | 0.01 | 61 (0.7) | 60 (0.7) | | -0.001 |
| Co-medications | | | | | | | |
| Anticoagulants | 1095 (4.3) | 386 (4.6) | 0.02 | 389 (4.7) | 375 (4.6) | | -0.008 |
| Antiplatelets | 17092 (66.9) | 6002 (71.8) | 0.11 | 5865 (71.3) | 5901 (71.8) | | 0.010 |
| Heparins | 908 (3.6) | 251 (3.0) | -0.03 | 239 (2.9) | 247 (3.0) | | 0.006 |
| Thrombolytics | 39 (0.2) | 8 (0.1) | -0.02 | 4 (0.1) | 8 (0.1) | | 0.018 |
| Statins | 17792 (69.6) | 6775 (81.1) | 0.27 | 6665 (81.1) | 6644 (80.8) | | -0.007 |
| Other lipid  Lowerings | 2708 (10.6) | 1239 (14.8) | 0.13 | 1186 (14.4) | 1184 (14.4) | | -0.001 |
| Nitrates | 4349 (17.0) | 1800 (21.5) | 0.11 | 1819 (22.1) | 1749 (21.3) | | -0.021 |
| Digoxin | 3736 (14.6) | 1577 (18.9) | 0.11 | 1574 (19.1) | 1528 (18.6) | | -0.014 |
| ACEIs | 1550 (6.1) | 749 (9.0) | 0.11 | 711 (8.7) | 708 (8.6) | | -0.001 |
| ARBs | 14830 (58.1) | 5195 (62.2) | 0.08 | 5077 (61.7) | 5099 (62.0) | | 0.006 |
| Entresto | 4 (0.0) | 15 (0.2) | 0.05 | 4 (0.1) | 5 (0.1) | | 0.005 |
| Other  Anti HTNs | 16593 (65.0) | 5884 (70.4) | 0.12 | 5749 (69.9) | 5764 (70.1) | | 0.004 |
| Loop diuretics | 2571 (10.1) | 914 (10.9) | 0.03 | 870 (10.6) | 873 (10.6) | | 0.001 |
| Other diuretics | 6630 (26.0) | 2226 (26.6) | 0.02 | 2217 (27.0) | 2168 (26.4) | | -0.014 |
| Antianxieties | 9460 (37.0) | 2687 (32.2) | -0.10 | 2660 (32.4) | 2650 (32.2) | | -0.003 |
| Antipsychotics | 995 (3.9) | 185 (2.2) | -0.10 | 190 (2.3) | 185 (2.3) | | -0.004 |
| Antidepressants | 4030 (15.8) | 1053 (12.6) | -0.09 | 1064 (12.9) | 1043 (12.7) | | -0.008 |
| Dementia | 3515 (13.8) | 654 (7.8) | -0.19 | 628 (7.6) | 652 (7.9) | | 0.011 |
| Antiparkinsons | 656 (2.6) | 110 (1.3) | -0.09 | 109 (1.3) | 110 (1.3) | | 0.001 |
| Anticonvulsants | 565 (2.2) | 119 (1.4) | -0.06 | 109 (1.3) | 119 (1.5) | | 0.010 |
| NSAIDs | 18728 (73.3) | 6054 (72.4) | -0.02 | 6012 (73.1) | 5963 (72.5) | | -0.013 |
| Bisphospho-nates | 898 (3.5) | 187 (2.2) | -0.08 | 184 (2.2) | 187 (2.3) | | 0.003 |
| Opioids | 10423 (40.8) | 3154 (37.7) | -0.06 | 3117 (37.9) | 3104 (37.8) | | -0.003 |

Values are represented as mean ± standard deviation or number (%); ACEis, angiotensin-converting enzyme inhibitors; AGIs, α-glucosidase Inhibitors; ARBs, angiotensin II receptor blockers; CABG, coronary artery bypass graft; CAD, coronary artery disease; COPD, chronic obstructive pulmonary disease; CV, cardiovascular; DM, diabetes mellitus; HONK, hyperglycaemic hyperosmolar nonketotic coma; HTN, hypertensive; MI, myocardial infarction; NSAIDs, non-steroidal anti-inflammatory drugs; PAD, peripheral artery disease; PUD, peptic ulcer disease; STD, standardized difference; SUs, sulfonylureas; UC, ulcerative colitis;

**Supplementary Table S8 Baseline characteristics of cohort for genital infections**

|  | **Pre-Match** | | **STD** | **Post-Match** | | | **STD** |
| --- | --- | --- | --- | --- | --- | --- | --- |
|  | **Sitagliptin**  **N= 29,638** | **Empagliflozin**  **N= 9,220** |  | **Sitagliptin**  **N= 9,107** | **Empagliflozin**  **N= 9,107** | |  |
| Sex | 17594 (59.4) | 5978 (64.8) | -0.04 | 5908 (64.9) | 5885 (64.6) | | -0.003 |
| Age | 60.6 ± 11.4 | 55.7 ± 11.1 | -0.44 | 55.9 ± 11.4 | 55.9 ± 10.9 | | 0.005 |
| Insurance type | | | | | | | |
| Normal | 27696 (93.5) | 8728 (94.7) | 0.05 | 8619 (94.6) | | 8622 (94.7) | 0.017 |
| Medicaid | 1777 (6.0) | 460 (5.0) |  | 464 (5.1) | 453 (5.0) | |  |
| No charge | 165 (0.6) | 32 (0.4) |  | 24 (0.3) | 32 (0.4) | |  |
| Number of  Inpatient visit | 0.8 ± 2.3 | 0.5 ± 1.2 | -0.21 | 0.5 ± 1.2 | 0.5 ± 1.2 | | -0.005 |
| Number of  outpatient visit | 26.5 ± 26.5 | 24.0 ± 22.7 | -0.10 | 24.1 ± 24.1 | 24.0 ± 22.8 | | -0.003 |
| Index year | | | | | | | |
| 2016 | 8831 (29.8) | 1837 (19.9) | 0.23 | 1817 (20.0) | 1836 (20.2) | | 0.016 |
| 2017 | 14655 (49.5) | 5058 (54.9) |  | 4948 (54.3) | 4993 (54.8) | |  |
| 2018 | 6152 (20.8) | 2325 (25.2) |  | 2342 (25.7) | 2278 (25.0) | |  |
| Charlson comorbidity index | | | | | | | |
| 0 | 1344 (4.5) | 404 (4.4) | 0.02 | 394 (4.3) | 399 (4.4) | | 0.009 |
| 1 | 2820 (9.5) | 937 (10.2) |  | 923 (10.1) | 930 (10.2) | |  |
| 2 | 4123 (13.9) | 1297 (14.1) |  | 1265 (13.9) | 1287 (14.1) | |  |
| 3 | 21351 (72.0) | 6582 (71.4) |  | 6525 (71.7) | 6491 (71.3) | |  |
| CV risk factor | | | | | | | |
| CAD | 26104 (88.1) | 8527 (92.5) | 0.15 | 8395 (92.2) | 8417 (92.4) | | 0.009 |
| Multi vessel CAD | 13114 (44.3) | 4969 (53.9) | 0.19 | 4822 (53.0) | 4873 (53.5) | | 0.011 |
| MI | 1638 (5.5) | 788 (8.6) | 0.12 | 733 (8.1) | 755 (8.3) | | 0.009 |
| CABG | 6205 (20.9) | 2730 (29.6) | 0.20 | 2662 (29.2) | 2653 (29.1) | | -0.002 |
| Stroke | 4395 (14.8) | 862 (9.4) | -0.17 | 887 (9.7) | 855 (9.4) | | -0.012 |
| PAD | 1865 (6.3) | 510 (5.5) | -0.03 | 501 (5.5) | 507 (5.6) | | 0.003 |
| DM circulation | 3920 (13.2) | 1394 (15.1) | 0.05 | 1381 (15.2) | 1374 (15.1) | | -0.002 |
| DM foot | 2 (0.0) | 4 (0.0) | 0.02 | 1 (0.0) | 2 (0.0) | | 0.009 |
| DM nephropathy | 1877 (6.3) | 782 (8.5) | 0.08 | 718 (7.9) | 765 (8.4) | | 0.019 |
| DM neuropathy | 4103 (13.8) | 1221 (13.2) | -0.02 | 1196 (13.1) | 1212 (13.3) | | 0.005 |
| DM other  Complications | 21790 (73.5) | 6550 (71.0) | -0.06 | 6516 (71.6) | 6479 (71.1) | | -0.009 |
| Hyperglycemia | 533 (1.8) | 127 (1.4) | -0.03 | 123 (1.4) | 124 (1.4) | | 0.001 |
| Comorbidities | | | | | | | |
| Hypertension | 23389 (78.9) | 7307 (79.3) | 0.01 | 7189 (78.9) | 7209 (79.2) | | 0.005 |
| Edema | 2681 (9.1) | 789 (8.6) | -0.02 | 794 (8.7) | 777 (8.5) | | -0.007 |
| Kidney stone | 405 (1.4) | 106 (1.2) | -0.02 | 109 (1.2) | 106 (1.2) | | -0.003 |
| Osteoarthritis | 10015 (33.8) | 2625 (28.5) | -0.12 | 2623 (28.8) | 2620 (28.8) | | -0.001 |
| Other arthritis | 7035 (23.7) | 1909 (20.7) | -0.07 | 1925 (21.1) | 1896 (20.8) | | -0.008 |
| PUD | 7125 (24.0) | 2105 (22.8) | -0.03 | 2084 (22.9) | 2089 (22.9) | | 0.001 |
| Pancreatitis | 265 (0.9) | 75 (0.8) | -0.01 | 82 (0.9) | 74 (0.8) | | -0.010 |
| UC | 41 (0.1) | 10 (0.1) | -0.01 | 9 (0.1) | 10 (0.1) | | 0.003 |
| Crohn | 10 (0.0) | 5 (0.1) | 0.01 | 4 (0.0) | 5 (0.1) | | 0.005 |
| Asthma | 4197 (14.2) | 1198 (13.0) | -0.03 | 1200 (13.2) | 1187 (13.0) | | -0.004 |
| COPD | 1095 (3.7) | 234 (2.5) | -0.07 | 248 (2.7) | 234 (2.6) | | -0.010 |
| Bladder stone | 19 (0.1) | 3 (0.0) | -0.01 | 1 (0.0) | 3 (0.0) | | 0.015 |
| Dementia | 4731 (16.0) | 880 (9.5) | -0.19 | 857 (9.4) | 877 (9.6) | | 0.008 |
| Electrolyte  Imbalance | 1837 (6.2) | 469 (5.1) | -0.05 | 425 (4.7) | 460 (5.1) | | 0.018 |
| Glaucoma  /Cataract | 8090 (27.3) | 2419 (26.2) | -0.02 | 2374 (26.1) | 2399 (26.3) | | 0.006 |
| HONK | 224 (0.8) | 52 (0.6) | -0.02 | 55 (0.6) | 50 (0.6) | | -0.007 |
| HTN  nephropathy | 128 (0.4) | 47 (0.5) | 0.01 | 49 (0.5) | 45 (0.5) | | -0.006 |
| Hyperthyroid  disease | 525 (1.8) | 165 (1.8) | 0.00 | 159 (1.8) | 163 (1.8) | | 0.003 |
| Hypothyroid  disease | 1337 (4.5) | 443 (4.8) | 0.01 | 457 (5.0) | 433 (4.8) | | -0.012 |
| Osteomyelitis | 218 (0.7) | 53 (0.6) | -0.02 | 53 (0.6) | 53 (0.6) | | 0.000 |
| Pneumonia | 2241 (7.6) | 570 (6.2) | -0.05 | 562 (6.2) | 563 (6.2) | | 0.001 |
| Skin infection | 1066 (3.6) | 331 (3.6) | 0.00 | 322 (3.5) | 327 (3.6) | | 0.003 |
| Glucose-lowering therapy | | | | | | | |
| Metformin | 20702 (69.9) | 6652 (72.2) | 0.05 | 6577 (72.2) | 6571 (72.2) | | -0.002 |
| Insulins | 4923 (16.6) | 1637 (17.8) | 0.03 | 1579 (17.3) | 1597 (17.5) | | 0.005 |
| SUs | 13584 (45.8) | 4368 (47.4) | 0.03 | 4312 (47.4) | 4314 (47.4) | | 0.000 |
| Glitazones | 2617 (8.8) | 1069 (11.6) | 0.09 | 1053 (11.6) | 1047 (11.5) | | -0.002 |
| GLP-1 agonists | 70 (0.2) | 65 (0.7) | 0.07 | 55 (0.6) | 56 (0.6) | | 0.001 |
| AGIs | 1267 (4.3) | 294 (3.2) | -0.06 | 275 (3.0) | 292 (3.2) | | 0.011 |
| Meglitinides | 200 (0.7) | 70 (0.8) | 0.01 | 73 (0.8) | 66 (0.7) | | -0.009 |
| Co-medications | | | | | | | |
| Anticoagulants | 1387 (4.7) | 459 (5.0) | 0.01 | 438 (4.8) | 450 (4.9) | | 0.006 |
| Antiplatelets | 19993 (67.5) | 6609 (71.7) | 0.09 | 6570 (72.1) | 6518 (71.6) | | -0.013 |
| Heparins | 1062 (3.6) | 284 (3.1) | -0.03 | 283 (3.1) | 283 (3.1) | | 0.000 |
| Thrombolytics | 48 (0.2) | 9 (0.1) | -0.02 | 7 (0.1) | 9 (0.1) | | 0.007 |
| Statins | 20801 (70.2) | 7451 (80.8) | 0.25 | 7325 (80.4) | 7341 (80.6) | | 0.004 |
| Other lipid  Lowerings | 3120 (10.5) | 1347 (14.6) | 0.12 | 1300 (14.3) | 1305 (14.3) | | 0.002 |
| Nitrates | 5090 (17.2) | 1961 (21.3) | 0.10 | 1927 (21.2) | 1911 (21.0) | | -0.004 |
| Digoxin | 4341 (14.7) | 1707 (18.5) | 0.10 | 1662 (18.3) | 1657 (18.2) | | -0.001 |
| ACEIs | 1804 (6.1) | 803 (8.7) | 0.10 | 754 (8.3) | 769 (8.4) | | 0.006 |
| ARBs | 17464 (58.9) | 5792 (62.8) | 0.08 | 5660 (62.2) | 5702 (62.6) | | 0.010 |
| Entresto | 6 (0.0) | 12 (0.1) | 0.04 | 4 (0.0) | 6 (0.1) | | 0.009 |
| Other  Anti HTNs | 19587 (66.1) | 6520 (70.7) | 0.10 | 6438 (70.7) | 6420 (70.5) | | -0.004 |
| Loop diuretics | 3457 (11.7) | 1102 (12.0) | 0.01 | 1037 (11.4) | 1067 (11.7) | | 0.010 |
| Other diuretics | 8112 (27.4) | 2545 (27.6) | 0.01 | 2435 (26.7) | 2489 (27.3) | | 0.013 |
| Antianxieties | 11436 (38.6) | 3124 (33.9) | -0.10 | 3055 (33.6) | 3101 (34.1) | | 0.011 |
| Antipsychotics | 1378 (4.7) | 235 (2.6) | -0.11 | 232 (2.6) | 235 (2.6) | | 0.002 |
| Antidepressants | 5076 (17.1) | 1296 (14.1) | -0.08 | 1309 (14.4) | 1290 (14.2) | | -0.006 |
| Dementia | 4731 (16.0) | 880 (9.5) | -0.19 | 857 (9.4) | 877 (9.6) | | 0.008 |
| Antiparkinsons | 879 (3.0) | 132 (1.4) | -0.10 | 115 (1.3) | 132 (1.5) | | 0.016 |
| Anticonvulsants | 716 (2.4) | 135 (1.5) | -0.07 | 142 (1.6) | 135 (1.5) | | -0.006 |
| NSAIDs | 21866 (73.8) | 6736 (73.1) | -0.02 | 6656 (73.1) | 6658 (73.1) | | 0.001 |
| Bisphospho-nates | 1328 (4.5) | 271 (2.9) | -0.08 | 285 (3.1) | 271 (3.0) | | -0.009 |
| Opioids | 12629 (42.6) | 3619 (39.3) | -0.07 | 3605 (39.6) | 3578 (39.3) | | -0.006 |

Values are represented as mean ± standard deviation or number (%); ACEis, angiotensin-converting enzyme inhibitors; AGIs, α-glucosidase Inhibitors; ARBs, angiotensin II receptor blockers; CABG, coronary artery bypass graft; CAD, coronary artery disease; COPD, chronic obstructive pulmonary disease; CV, cardiovascular; DM, diabetes mellitus; HONK, hyperglycaemic hyperosmolar nonketotic coma; HTN, hypertensive; MI, myocardial infarction; NSAIDs, non-steroidal anti-inflammatory drugs; PAD, peripheral artery disease; PUD, peptic ulcer disease; STD, standardized difference; SUs, sulfonylureas; UC, ulcerative colitis;

**Supplementary Table S9 Baseline characteristics of cohort for acute kidney injury**

|  | **Pre-Match** | | **STD** | **Post-Match** | | | **STD** |
| --- | --- | --- | --- | --- | --- | --- | --- |
|  | **Sitagliptin**  **N= 35,914** | **Empagliflozin**  **N= 11,399** |  | **Sitagliptin**  **N= 11,278** | **Empagliflozin**  **N= 11,278** | |  |
| Sex | 19791 (55.1) | 6724 (59.0) | -0.03 | 33593 (93.5) | 10796 (94.7) | | 0.001 |
| Age | 60.3 ± 11.4 | 55.4 ± 10.9 | -0.44 | 55.5 ± 11.3 | 55.6 ± 10.8 | | 0.004 |
| Insurance type | | | | | | | |
| Normal | 33593 (93.5) | 10796 (94.7) | 0.05 | 10685 (94.7) | | 10683 (94.7) | 0.002 |
| Medicaid | 2138 (6.0) | 568 (5.0) |  | 559 (5.0) | 560 (5.0) | |  |
| No charge | 183 (0.5) | 35 (0.3) |  | 34 (0.3) | 35 (0.3) | |  |
| Number of  Inpatient visit | 0.8 ± 2.2 | 0.5 ± 1.2 | -0.20 | 0.5 ± 1.2 | 0.5 ± 1.2 | | -0.013 |
| Number of  outpatient visit | 28.0 ± 27.4 | 25.4 ± 23.6 | -0.10 | 25.6 ± 23.5 | 25.5 ± 23.7 | | -0.006 |
| Index year | | | | | | | |
| 2016 | 10312 (28.7) | 2209 (19.4) | 0.22 | 2228 (19.8) | 2207 (19.6) | | 0.014 |
| 2017 | 17818 (49.6) | 6177 (54.2) |  | 6164 (54.7) | 6116 (54.2) | |  |
| 2018 | 7784 (21.7) | 3013 (26.4) |  | 2886 (25.6) | 2955 (26.2) | |  |
| Charlson comorbidity index | | | | | | | |
| 0 | 1516 (4.2) | 470 (4.1) | 0.03 | 467 (4.1) | 467 (4.1) | | 0.013 |
| 1 | 3345 (9.3) | 1138 (10.0) |  | 1090 (9.7) | 1127 (10.0) | |  |
| 2 | 4876 (13.6) | 1627 (14.3) |  | 1594 (14.1) | 1613 (14.3) | |  |
| 3 | 26177 (72.9) | 8164 (71.6) |  | 8127 (72.1) | 8071 (71.6) | |  |
| CV risk factor | | | | | | | |
| CAD | 31760 (88.4) | 10545 (92.5) | 0.14 | 10368 (91.9) | 10428 (92.5) | | 0.020 |
| Multi vessel CAD | 15760 (43.9) | 5985 (52.5) | 0.17 | 5773 (51.2) | 5880 (52.1) | | 0.019 |
| MI | 1831 (5.1) | 875 (7.7) | 0.11 | 826 (7.3) | 836 (7.4) | | 0.003 |
| CABG | 7419 (20.7) | 3219 (28.2) | 0.18 | 3113 (27.6) | 3138 (27.8) | | 0.005 |
| Stroke | 5078 (14.1) | 1020 (9.0) | -0.16 | 1063 (9.4) | 1016 (9.0) | | -0.014 |
| PAD | 2299 (6.4) | 652 (5.7) | -0.03 | 662 (5.9) | 648 (5.8) | | -0.005 |
| DM circulation | 4762 (13.3) | 1754 (15.4) | 0.06 | 1753 (15.5) | 1721 (15.3) | | -0.008 |
| DM foot | 2 (0.0) | 3 (0.0) | 0.02 | 1 (0.0) | 3 (0.0) | | 0.013 |
| DM nephropathy | 2239 (6.2) | 969 (8.5) | 0.09 | 956 (8.5) | 945 (8.4) | | -0.004 |
| DM neuropathy | 5091 (14.2) | 1547 (13.6) | -0.02 | 1581 (14.0) | 1530 (13.6) | | -0.013 |
| DM other  Complications | 26592 (74.0) | 8138 (71.4) | -0.06 | 8062 (71.5) | 8073 (71.6) | | 0.002 |
| Hyperglycemia | 650 (1.8) | 148 (1.3) | -0.04 | 155 (1.4) | 145 (1.3) | | -0.008 |
| Comorbidities | | | | | | | |
| Hypertension | 28045 (78.1) | 8967 (78.7) | 0.01 | 8858 (78.5) | 8864 (78.6) | | 0.001 |
| Edema | 3325 (9.3) | 1027 (9.0) | -0.01 | 994 (8.8) | 1013 (9.0) | | 0.006 |
| Kidney stone | 555 (1.6) | 157 (1.4) | -0.01 | 167 (1.5) | 156 (1.4) | | -0.008 |
| Osteoarthritis | 12790 (35.6) | 3476 (30.5) | -0.11 | 3556 (31.5) | 3459 (30.7) | | -0.019 |
| Other arthritis | 8799 (24.5) | 2482 (21.8) | -0.06 | 2448 (21.7) | 2463 (21.8) | | 0.003 |
| PUD | 9104 (25.4) | 2738 (24.0) | -0.03 | 2773 (24.6) | 2713 (24.1) | | -0.012 |
| Pancreatitis | 319 (0.9) | 101 (0.9) | 0.00 | 109 (1.0) | 100 (0.9) | | -0.008 |
| UC | 57 (0.2) | 12 (0.1) | -0.01 | 10 (0.1) | 12 (0.1) | | 0.006 |
| Crohn | 14 (0.0) | 4 (0.0) | 0.00 | 6 (0.1) | 4 (0.0) | | -0.008 |
| Asthma | 5241 (14.6) | 1580 (13.9) | -0.02 | 1575 (14.0) | 1560 (13.8) | | -0.004 |
| COPD | 1277 (3.6) | 278 (2.4) | -0.07 | 294 (2.6) | 278 (2.5) | | -0.009 |
| Bladder stone | 27 (0.1) | 4 (0.0) | -0.02 | 7 (0.1) | 4 (0.0) | | -0.012 |
| Dementia | 5740 (16.0) | 1106 (9.7) | -0.19 | 1101 (9.8) | 1104 (9.8) | | 0.001 |
| Electrolyte  Imbalance | 2151 (6.0) | 561 (4.9) | -0.05 | 563 (5.0) | 551 (4.9) | | -0.005 |
| Glaucoma  /Cataract | 10232 (28.5) | 3087 (27.1) | -0.03 | 3081 (27.3) | 3062 (27.2) | | -0.004 |
| HONK | 276 (0.8) | 60 (0.5) | -0.03 | 66 (0.6) | 60 (0.5) | | -0.007 |
| HTN  nephropathy | 154 (0.4) | 50 (0.4) | 0.00 | 53 (0.5) | 48 (0.4) | | -0.007 |
| Hyperthyroid  disease | 676 (1.9) | 222 (2.0) | 0.00 | 231 (2.1) | 220 (2.0) | | -0.007 |
| Hypothyroid  disease | 1737 (4.8) | 578 (5.1) | 0.01 | 533 (4.7) | 567 (5.0) | | 0.014 |
| Osteomyelitis | 261 (0.7) | 65 (0.6) | -0.02 | 72 (0.6) | 65 (0.6) | | -0.008 |
| Pneumonia | 2710 (7.6) | 746 (6.5) | -0.04 | 757 (6.7) | 736 (6.5) | | -0.008 |
| Skin infection | 1403 (3.9) | 445 (3.9) | 0.00 | 470 (4.2) | 440 (3.9) | | -0.014 |
| Glucose-lowering therapy | | | | | | | |
| Metformin | 25217 (70.2) | 8276 (72.6) | 0.05 | 8263 (73.3) | 8186 (72.6) | | -0.015 |
| Insulins | 5952 (16.6) | 2035 (17.9) | 0.03 | 1990 (17.6) | 1988 (17.6) | | -0.001 |
| SUs | 16426 (45.7) | 5342 (46.9) | 0.02 | 5323 (47.2) | 5283 (46.8) | | -0.007 |
| Glitazones | 3190 (8.9) | 1289 (11.3) | 0.08 | 1271 (11.3) | 1262 (11.2) | | -0.003 |
| GLP-1 agonists | 105 (0.3) | 78 (0.7) | 0.06 | 80 (0.7) | 69 (0.6) | | -0.012 |
| AGIs | 1478 (4.1) | 355 (3.1) | -0.05 | 358 (3.2) | 353 (3.1) | | -0.003 |
| Meglitinides | 233 (0.7) | 83 (0.7) | 0.01 | 78 (0.7) | 81 (0.7) | | 0.003 |
| Co-medications | | | | | | | |
| Anticoagulants | 1575 (4.4) | 538 (4.7) | 0.02 | 507 (4.5) | 526 (4.7) | | 0.008 |
| Antiplatelets | 23836 (66.4) | 8019 (70.4) | 0.09 | 7947 (70.5) | 7928 (70.3) | | -0.004 |
| Heparins | 1229 (3.4) | 339 (3.0) | -0.03 | 330 (2.9) | 335 (3.0) | | 0.003 |
| Thrombolytics | 54 (0.2) | 9 (0.1) | -0.02 | 8 (0.1) | 9 (0.1) | | 0.003 |
| Statins | 25306 (70.5) | 9223 (80.9) | 0.25 | 9113 (80.8) | 9106 (80.7) | | -0.002 |
| Other lipid  Lowerings | 3804 (10.6) | 1628 (14.3) | 0.11 | 1626 (14.4) | 1580 (14.0) | | -0.012 |
| Nitrates | 6070 (16.9) | 2369 (20.8) | 0.10 | 2340 (20.8) | 2315 (20.5) | | -0.006 |
| Digoxin | 5227 (14.6) | 2068 (18.1) | 0.10 | 2038 (18.1) | 2016 (17.9) | | -0.005 |
| ACEIs | 2054 (5.7) | 932 (8.2) | 0.10 | 904 (8.0) | 901 (8.0) | | -0.001 |
| ARBs | 20924 (58.3) | 7097 (62.3) | 0.08 | 7050 (62.5) | 7007 (62.1) | | -0.008 |
| Entresto | 5 (0.0) | 17 (0.2) | 0.05 | 4 (0.0) | 6 (0.1) | | 0.008 |
| Other  Anti HTNs | 23439 (65.3) | 7910 (69.4) | 0.09 | 7850 (69.6) | 7810 (69.3) | | -0.008 |
| Loop diuretics | 4014 (11.2) | 1281 (11.2) | 0.00 | 1267 (11.2) | 1243 (11.0) | | -0.007 |
| Other diuretics | 9668 (26.9) | 3128 (27.4) | 0.01 | 3047 (27.0) | 3072 (27.2) | | 0.005 |
| Antianxieties | 14514 (40.4) | 4096 (35.9) | -0.09 | 4093 (36.3) | 4068 (36.1) | | -0.005 |
| Antipsychotics | 1679 (4.7) | 284 (2.5) | -0.12 | 285 (2.5) | 284 (2.5) | | -0.001 |
| Antidepressants | 6420 (17.9) | 1713 (15.0) | -0.08 | 1734 (15.4) | 1705 (15.1) | | -0.007 |
| Dementia | 5740 (16.0) | 1106 (9.7) | -0.19 | 1101 (9.8) | 1104 (9.8) | | 0.001 |
| Antiparkinsons | 1090 (3.0) | 170 (1.5) | -0.10 | 158 (1.4) | 170 (1.5) | | 0.009 |
| Anticonvulsants | 889 (2.5) | 178 (1.6) | -0.07 | 171 (1.5) | 178 (1.6) | | 0.005 |
| NSAIDs | 27323 (76.1) | 8609 (75.5) | -0.01 | 8518 (75.5) | 8522 (75.6) | | 0.001 |
| Bisphospho-nates | 1699 (4.7) | 364 (3.2) | -0.08 | 344 (3.1) | 364 (3.2) | | 0.010 |
| Opioids | 15869 (44.2) | 4655 (40.8) | -0.07 | 4671 (41.4) | 4610 (40.9) | | -0.011 |

Values are represented as mean ± standard deviation or number (%); ACEis, angiotensin-converting enzyme inhibitors; AGIs, α-glucosidase Inhibitors; ARBs, angiotensin II receptor blockers; CABG, coronary artery bypass graft; CAD, coronary artery disease; COPD, chronic obstructive pulmonary disease; CV, cardiovascular; DM, diabetes mellitus; HONK, hyperglycaemic hyperosmolar nonketotic coma; HTN, hypertensive; MI, myocardial infarction; NSAIDs, non-steroidal anti-inflammatory drugs; PAD, peripheral artery disease; PUD, peptic ulcer disease; STD, standardized difference; SUs, sulfonylureas; UC, ulcerative colitis;

**Supplementary Table S10 Baseline characteristics of cohort for volume depletion**

|  | **Pre-Match** | | **STD** | **Post-Match** | | | **STD** |
| --- | --- | --- | --- | --- | --- | --- | --- |
|  | **Sitagliptin**  **N= 32,945** | **Empagliflozin**  **N= 10,575** |  | **Sitagliptin**  **N= 32,945** | **Empagliflozin** | |  |
| Sex | 18390 (55.8) | 6328 (59.8) | -0.04 | 6253 (59.8) | 6251 (59.7) | | -0.003 |
| Age | 60.1 ± 11.3 | 55.3 ± 10.9 | -0.44 | 55.3 ± 11.2 | 55.4 ± 10.8 | | 0.016 |
| Insurance type | | | | | | | |
| Normal | 30904 (93.8) | 10053 (95.1) | 0.06 | 9937 (95.0) | | 9948 (95.1) | 0.013 |
| Medicaid | 1882 (5.7) | 490 (4.6) |  | 503 (4.8) | 486 (4.6) | |  |
| No charge | 159 (0.5) | 32 (0.3) |  | 26 (0.3) | 32 (0.3) | |  |
| Number of  Inpatient visit | 0.8 ± 2.1 | 0.4 ± 1.1 | -0.20 | 0.4 ± 1.1 | 0.4 ± 1.1 | | -0.009 |
| Number of  outpatient visit | 27.3 ± 26.6 | 24.7 ± 22.9 | -0.10 | 24.9 ± 23.3 | 24.8 ± 23.0 | | -0.006 |
| Index year | | | | | | | |
| 2016 | 9581 (29.1) | 2075 (19.6) | 0.23 | 2080 (19.9) | 2073 (19.8) | | 0.002 |
| 2017 | 16301 (49.5) | 5719 (54.1) |  | 5654 (54.0) | 5662 (54.1) | |  |
| 2018 | 7063 (21.4) | 2781 (26.3) |  | 2732 (26.1) | 2731 (26.1) | |  |
| Charlson comorbidity index | | | | | | | |
| 0 | 1442 (4.4) | 455 (4.3) | 0.03 | 479 (4.6) | 449 (4.3) | | 0.024 |
| 1 | 3134 (9.5) | 1086 (10.3) |  | 1013 (9.7) | 1077 (10.3) | |  |
| 2 | 4575 (13.9) | 1531 (14.5) |  | 1541 (14.7) | 1519 (14.5) | |  |
| 3 | 23794 (72.2) | 7503 (71.0) |  | 7433 (71.0) | 7421 (70.9) | |  |
| CV risk factor | | | | | | | |
| CAD | 29110 (88.4) | 9794 (92.6) | 0.15 | 9629 (92.0) | 9688 (92.6) | | 0.021 |
| Multi vessel CAD | 14399 (43.7) | 5559 (52.6) | 0.18 | 5296 (50.6) | 5470 (52.3) | | 0.033 |
| MI | 1664 (5.1) | 810 (7.7) | 0.11 | 767 (7.3) | 781 (7.5) | | 0.005 |
| CABG | 6760 (20.5) | 2976 (28.1) | 0.18 | 2873 (27.5) | 2910 (27.8) | | 0.008 |
| Stroke | 4601 (14.0) | 913 (8.6) | -0.17 | 922 (8.8) | 910 (8.7) | | -0.004 |
| PAD | 2094 (6.4) | 586 (5.5) | -0.03 | 595 (5.7) | 582 (5.6) | | -0.005 |
| DM circulation | 4349 (13.2) | 1606 (15.2) | 0.06 | 1607 (15.4) | 1585 (15.1) | | -0.006 |
| DM foot | 2 (0.0) | 2 (0.0) | 0.01 | 1 (0.0) | 2 (0.0) | | 0.008 |
| DM nephropathy | 2106 (6.4) | 914 (8.6) | 0.09 | 887 (8.5) | 891 (8.5) | | 0.001 |
| DM neuropathy | 4625 (14.0) | 1413 (13.4) | -0.02 | 1418 (13.6) | 1399 (13.4) | | -0.005 |
| DM other  Complications | 24263 (73.7) | 7517 (71.1) | -0.06 | 7471 (71.4) | 7449 (71.2) | | -0.005 |
| Hyperglycemia | 559 (1.7) | 130 (1.2) | -0.04 | 116 (1.1) | 128 (1.2) | | 0.011 |
| Comorbidities | | | | | | | |
| Hypertension | 25733 (78.1) | 8325 (78.7) | 0.01 | 8200 (78.4) | 8235 (78.7) | | 0.008 |
| Edema | 2983 (9.1) | 917 (8.7) | -0.01 | 919 (8.8) | 904 (8.6) | | -0.005 |
| Kidney stone | 510 (1.6) | 144 (1.4) | -0.02 | 149 (1.4) | 144 (1.4) | | -0.004 |
| Osteoarthritis | 11445 (34.7) | 3141 (29.7) | -0.11 | 3124 (29.9) | 3132 (29.9) | | 0.002 |
| Other arthritis | 7852 (23.8) | 2277 (21.5) | -0.06 | 2267 (21.7) | 2259 (21.6) | | -0.002 |
| PUD | 8128 (24.7) | 2492 (23.6) | -0.03 | 2488 (23.8) | 2467 (23.6) | | -0.005 |
| Pancreatitis | 275 (0.8) | 86 (0.8) | 0.00 | 89 (0.9) | 85 (0.8) | | -0.004 |
| UC | 55 (0.2) | 11 (0.1) | -0.02 | 14 (0.1) | 11 (0.1) | | -0.008 |
| Crohn | 13 (0.0) | 4 (0.0) | 0.00 | 3 (0.0) | 4 (0.0) | | 0.005 |
| Asthma | 4680 (14.2) | 1437 (13.6) | -0.02 | 1419 (13.6) | 1418 (13.6) | | 0.000 |
| COPD | 1117 (3.4) | 251 (2.4) | -0.06 | 256 (2.5) | 251 (2.4) | | -0.003 |
| Bladder stone | 28 (0.1) | 3 (0.0) | -0.02 | 4 (0.0) | 3 (0.0) | | -0.005 |
| Dementia | 5064 (15.4) | 986 (9.3) | -0.18 | 988 (9.4) | 983 (9.4) | | -0.002 |
| Electrolyte  Imbalance | 1884 (5.7) | 511 (4.8) | -0.04 | 517 (4.9) | 504 (4.8) | | -0.006 |
| Glaucoma  /Cataract | 9274 (28.2) | 2803 (26.5) | -0.04 | 2797 (26.7) | 2782 (26.6) | | -0.003 |
| HONK | 239 (0.7) | 59 (0.6) | -0.02 | 60 (0.6) | 58 (0.6) | | -0.003 |
| HTN  nephropathy | 145 (0.4) | 49 (0.5) | 0.00 | 55 (0.5) | 47 (0.5) | | -0.011 |
| Hyperthyroid  disease | 613 (1.9) | 195 (1.8) | 0.00 | 207 (2.0) | 193 (1.8) | | -0.010 |
| Hypothyroid  disease | 1565 (4.8) | 526 (5.0) | 0.01 | 515 (4.9) | 517 (4.9) | | 0.001 |
| Osteomyelitis | 244 (0.7) | 61 (0.6) | -0.02 | 70 (0.7) | 61 (0.6) | | -0.011 |
| Pneumonia | 2405 (7.3) | 674 (6.4) | -0.04 | 648 (6.2) | 663 (6.3) | | 0.006 |
| Skin infection | 1263 (3.8) | 398 (3.8) | 0.00 | 348 (3.3) | 394 (3.8) | | 0.024 |
| Glucose-lowering therapy | | | | | | | |
| Metformin | 23128 (70.2) | 7651 (72.4) | 0.05 | 7551 (72.2) | 7571 (72.3) | | 0.004 |
| Insulins | 5396 (16.4) | 1860 (17.6) | 0.03 | 1885 (18.0) | 1820 (17.4) | | -0.016 |
| SUs | 15086 (45.8) | 4967 (47.0) | 0.02 | 4808 (45.9) | 4913 (46.9) | | 0.020 |
| Glitazones | 2944 (8.9) | 1195 (11.3) | 0.08 | 1196 (11.4) | 1169 (11.2) | | -0.008 |
| GLP-1 agonists | 99 (0.3) | 71 (0.7) | 0.05 | 63 (0.6) | 66 (0.6) | | 0.004 |
| AGIs | 1362 (4.1) | 322 (3.0) | -0.06 | 312 (3.0) | 321 (3.1) | | 0.005 |
| Meglitinides | 217 (0.7) | 75 (0.7) | 0.01 | 77 (0.7) | 72 (0.7) | | -0.006 |
| Co-medications | | | | | | | |
| Anticoagulants | 1415 (4.3) | 504 (4.8) | 0.02 | 488 (4.7) | 494 (4.7) | | 0.003 |
| Antiplatelets | 21916 (66.5) | 7452 (70.5) | 0.09 | 7342 (70.2) | 7364 (70.4) | | 0.005 |
| Heparins | 1128 (3.4) | 314 (3.0) | -0.03 | 330 (3.2) | 313 (3.0) | | -0.009 |
| Thrombolytics | 48 (0.2) | 9 (0.1) | -0.02 | 9 (0.1) | 9 (0.1) | | 0.000 |
| Statins | 23258 (70.6) | 8550 (80.9) | 0.24 | 8415 (80.4) | 8445 (80.7) | | 0.007 |
| Other lipid  Lowerings | 3482 (10.6) | 1487 (14.1) | 0.11 | 1480 (14.1) | 1454 (13.9) | | -0.007 |
| Nitrates | 5554 (16.9) | 2202 (20.8) | 0.10 | 2108 (20.1) | 2152 (20.6) | | 0.010 |
| Digoxin | 4767 (14.5) | 1914 (18.1) | 0.10 | 1833 (17.5) | 1867 (17.8) | | 0.009 |
| ACEIs | 1908 (5.8) | 877 (8.3) | 0.10 | 836 (8.0) | 840 (8.0) | | 0.001 |
| ARBs | 19223 (58.4) | 6598 (62.4) | 0.08 | 6529 (62.4) | 6519 (62.3) | | -0.002 |
| Entresto | 6 (0.0) | 17 (0.2) | 0.05 | 6 (0.1) | 10 (0.1) | | 0.014 |
| Other  Anti HTNs | 21524 (65.3) | 7350 (69.5) | 0.09 | 7210 (68.9) | 7255 (69.3) | | 0.009 |
| Loop diuretics | 3682 (11.2) | 1195 (11.3) | 0.00 | 1134 (10.8) | 1164 (11.1) | | 0.009 |
| Other diuretics | 8883 (27.0) | 2899 (27.4) | 0.01 | 2827 (27.0) | 2847 (27.2) | | 0.004 |
| Antianxieties | 13011 (39.5) | 3679 (34.8) | -0.10 | 3649 (34.9) | 3645 (34.8) | | -0.001 |
| Antipsychotics | 1467 (4.5) | 260 (2.5) | -0.11 | 277 (2.7) | 260 (2.5) | | -0.010 |
| Antidepressants | 5660 (17.2) | 1499 (14.2) | -0.08 | 1531 (14.6) | 1490 (14.2) | | -0.011 |
| Dementia | 5064 (15.4) | 986 (9.3) | -0.18 | 988 (9.4) | 983 (9.4) | | -0.002 |
| Antiparkinsons | 934 (2.8) | 151 (1.4) | -0.10 | 162 (1.6) | 151 (1.4) | | -0.009 |
| Anticonvulsants | 766 (2.3) | 154 (1.5) | -0.06 | 156 (1.5) | 154 (1.5) | | -0.002 |
| NSAIDs | 24821 (75.3) | 7894 (74.7) | -0.02 | 7813 (74.7) | 7820 (74.7) | | 0.002 |
| Bisphospho-nates | 1506 (4.6) | 308 (2.9) | -0.09 | 312 (3.0) | 307 (2.9) | | -0.003 |
| Opioids | 14297 (43.4) | 4211 (39.8) | -0.07 | 4161 (39.8) | 4178 (39.9) | | 0.003 |

Values are represented as mean ± standard deviation or number (%); ACEis, angiotensin-converting enzyme inhibitors; AGIs, α-glucosidase Inhibitors; ARBs, angiotensin II receptor blockers; CABG, coronary artery bypass graft; CAD, coronary artery disease; COPD, chronic obstructive pulmonary disease; CV, cardiovascular; DM, diabetes mellitus; HONK, hyperglycaemic hyperosmolar nonketotic coma; HTN, hypertensive; MI, myocardial infarction; NSAIDs, non-steroidal anti-inflammatory drugs; PAD, peripheral artery disease; PUD, peptic ulcer disease; STD, standardized difference; SUs, sulfonylureas; UC, ulcerative colitis;

**Supplementary Table S11 Baseline characteristics of cohort for diabetic ketoacidosis**

|  | **Pre-Match** | | **STD** | **Post-Match** | | | **STD** |
| --- | --- | --- | --- | --- | --- | --- | --- |
|  | **Sitagliptin**  **N= 36,546** | **Empagliflozin**  **N= 11,588** |  | **Sitagliptin**  **N= 11,469** | **Empagliflozin**  **N= 11,469** | |  |
| Sex | 20108 (55.0) | 6861 (59.2) | -0.04 | 6752 (58.9) | 6774 (59.1) | | 0.008 |
| Age | 60.4 ± 11.4 | 55.4 ± 10.9 | -0.44 | 55.6 ± 11.2 | 55.6 ± 10.8 | | 0.003 |
| Insurance type | | | | | | | |
| Normal | 34152 (93.5) | 10975 (94.7) | 0.06 | 10844 (94.6) | | 10860 (94.7) | 0.010 |
| Medicaid | 2204 (6.0) | 577 (5.0) |  | 593 (5.2) | 573 (5.0) | |  |
| No charge | 190 (0.5) | 36 (0.3) |  | 32 (0.3) | 36 (0.3) | |  |
| Number of  Inpatient visit | 0.8 ± 2.2 | 0.5 ± 1.2 | -0.21 | 0.5 ± 1.2 | 0.5 ± 1.2 | | 0.001 |
| Number of  outpatient visit | 28.1 ± 27.5 | 25.6 ± 24.0 | -0.10 | 25.5 ± 24.0 | 25.6 ± 24.0 | | 0.006 |
| Index year | | | | | | | |
| 2016 | 10489 (28.7) | 2229 (19.2) | 0.23 | 2192 (19.1) | 2227 (19.4) | | 0.010 |
| 2017 | 18117 (49.6) | 6284 (54.2) |  | 6276 (54.7) | 6222 (54.3) | |  |
| 2018 | 7940 (21.7) | 3075 (26.5) |  | 3001 (26.2) | 3020 (26.3) | |  |
| Charlson comorbidity index | | | | | | | |
| 0 | 1515 (4.2) | 473 (4.1) | 0.03 | 447 (3.9) | 467 (4.1) | | 0.012 |
| 1 | 3344 (9.2) | 1138 (9.8) |  | 1147 (10.0) | 1132 (9.9) | |  |
| 2 | 4900 (13.4) | 1629 (14.1) |  | 1641 (14.3) | 1610 (14.0) | |  |
| 3 | 26787 (73.3) | 8348 (72.0) |  | 8234 (71.8) | 8260 (72.0) | |  |
| CV risk factor | | | | | | | |
| CAD | 32319 (88.4) | 10729 (92.6) | 0.14 | 10573 (92.2) | 10615 (92.6) | | 0.014 |
| Multi vessel CAD | 16087 (44.0) | 6100 (52.6) | 0.17 | 5868 (51.2) | 6000 (52.3) | | 0.023 |
| MI | 1903 (5.2) | 902 (7.8) | 0.10 | 820 (7.2) | 872 (7.6) | | 0.017 |
| CABG | 7602 (20.8) | 3309 (28.6) | 0.18 | 3134 (27.3) | 3235 (28.2) | | 0.020 |
| Stroke | 5242 (14.3) | 1049 (9.1) | -0.17 | 1045 (9.1) | 1043 (9.1) | | -0.001 |
| PAD | 2356 (6.5) | 659 (5.7) | -0.03 | 680 (5.9) | 655 (5.7) | | -0.009 |
| DM circulation | 4883 (13.4) | 1792 (15.5) | 0.06 | 1795 (15.7) | 1767 (15.4) | | -0.007 |
| DM foot | 3 (0.0) | 4 (0.0) | 0.02 | 2 (0.0) | 3 (0.0) | | 0.006 |
| DM nephropathy | 2338 (6.4) | 1001 (8.6) | 0.09 | 963 (8.4) | 972 (8.5) | | 0.003 |
| DM neuropathy | 5218 (14.3) | 1575 (13.6) | -0.02 | 1594 (13.9) | 1561 (13.6) | | -0.008 |
| DM other  Complications | 27116 (74.2) | 8288 (71.5) | -0.06 | 8205 (71.5) | 8217 (71.7) | | 0.002 |
| Hyperglycemia | 678 (1.9) | 155 (1.3) | -0.04 | 161 (1.4) | 153 (1.3) | | -0.006 |
| Comorbidities | | | | | | | |
| Hypertension | 28640 (78.4) | 9133 (78.8) | 0.01 | 8998 (78.5) | 9032 (78.8) | | 0.007 |
| Edema | 3458 (9.5) | 1057 (9.1) | -0.01 | 1079 (9.4) | 1042 (9.1) | | -0.011 |
| Kidney stone | 581 (1.6) | 165 (1.4) | -0.01 | 164 (1.4) | 164 (1.4) | | 0.000 |
| Osteoarthritis | 13057 (35.7) | 3546 (30.6) | -0.11 | 3564 (31.1) | 3538 (30.9) | | -0.005 |
| Other arthritis | 9027 (24.7) | 2544 (22.0) | -0.07 | 2536 (22.1) | 2524 (22.0) | | -0.003 |
| PUD | 9297 (25.4) | 2793 (24.1) | -0.03 | 2772 (24.2) | 2768 (24.1) | | -0.001 |
| Pancreatitis | 335 (0.9) | 103 (0.9) | 0.00 | 103 (0.9) | 102 (0.9) | | -0.001 |
| UC | 59 (0.2) | 12 (0.1) | -0.02 | 11 (0.1) | 12 (0.1) | | 0.003 |
| Crohn | 15 (0.0) | 5 (0.0) | 0.00 | 6 (0.1) | 5 (0.0) | | -0.004 |
| Asthma | 5375 (14.7) | 1615 (13.9) | -0.02 | 1636 (14.3) | 1598 (13.9) | | -0.010 |
| COPD | 1337 (3.7) | 294 (2.5) | -0.06 | 309 (2.7) | 294 (2.6) | | -0.008 |
| Bladder stone | 28 (0.1) | 5 (0.0) | -0.01 | 3 (0.0) | 5 (0.0) | | 0.009 |
| Dementia | 5932 (16.2) | 1144 (9.9) | -0.19 | 1130 (9.9) | 1144 (10.0) | | 0.004 |
| Electrolyte  Imbalance | 2314 (6.3) | 600 (5.2) | -0.05 | 574 (5.0) | 593 (5.2) | | 0.008 |
| Glaucoma  /Cataract | 10408 (28.5) | 3151 (27.2) | -0.03 | 3152 (27.5) | 3119 (27.2) | | -0.007 |
| HONK | 273 (0.8) | 62 (0.5) | -0.03 | 57 (0.5) | 61 (0.5) | | 0.005 |
| HTN  nephropathy | 165 (0.5) | 53 (0.5) | 0.00 | 63 (0.6) | 51 (0.4) | | -0.015 |
| Hyperthyroid  disease | 697 (1.9) | 224 (1.9) | 0.00 | 235 (2.1) | 223 (1.9) | | -0.008 |
| Hypothyroid  disease | 1777 (4.9) | 594 (5.1) | 0.01 | 577 (5.0) | 586 (5.1) | | 0.004 |
| Osteomyelitis | 279 (0.8) | 65 (0.6) | -0.03 | 70 (0.6) | 65 (0.6) | | -0.006 |
| Pneumonia | 2842 (7.8) | 763 (6.6) | -0.05 | 742 (6.5) | 755 (6.6) | | 0.005 |
| Skin infection | 1424 (3.9) | 455 (3.9) | 0.00 | 451 (3.9) | 452 (3.9) | | 0.000 |
| Glucose-lowering therapy | | | | | | | |
| Metformin | 25606 (70.1) | 8395 (72.5) | 0.05 | 8244 (71.9) | 8307 (72.4) | | 0.012 |
| Insulins | 6188 (16.9) | 2086 (18.0) | 0.03 | 2043 (17.8) | 2039 (17.8) | | -0.001 |
| SUs | 16734 (45.8) | 5450 (47.0) | 0.02 | 5349 (46.6) | 5385 (47.0) | | 0.006 |
| Glitazones | 3252 (8.9) | 1316 (11.4) | 0.08 | 1323 (11.5) | 1284 (11.2) | | -0.011 |
| GLP-1 agonists | 112 (0.3) | 77 (0.7) | 0.05 | 78 (0.7) | 72 (0.6) | | -0.007 |
| AGIs | 1511 (4.1) | 363 (3.1) | -0.05 | 359 (3.1) | 363 (3.2) | | 0.002 |
| Meglitinides | 250 (0.7) | 84 (0.7) | 0.00 | 79 (0.7) | 80 (0.7) | | 0.001 |
| Co-medications | | | | | | | |
| Anticoagulants | 1631 (4.5) | 551 (4.8) | 0.01 | 535 (4.7) | 541 (4.7) | | 0.003 |
| Antiplatelets | 24305 (66.5) | 8171 (70.5) | 0.09 | 8005 (69.8) | 8079 (70.4) | | 0.014 |
| Heparins | 1277 (3.5) | 351 (3.0) | -0.03 | 328 (2.9) | 349 (3.0) | | 0.011 |
| Thrombolytics | 57 (0.2) | 10 (0.1) | -0.02 | 7 (0.1) | 10 (0.1) | | 0.010 |
| Statins | 25765 (70.5) | 9378 (80.9) | 0.25 | 9239 (80.6) | 9261 (80.8) | | 0.005 |
| Other lipid  Lowerings | 3872 (10.6) | 1667 (14.4) | 0.11 | 1640 (14.3) | 1622 (14.1) | | -0.005 |
| Nitrates | 6222 (17.0) | 2419 (20.9) | 0.10 | 2355 (20.5) | 2375 (20.7) | | 0.004 |
| Digoxin | 5352 (14.6) | 2113 (18.2) | 0.10 | 2032 (17.7) | 2070 (18.1) | | 0.009 |
| ACEIs | 2108 (5.8) | 951 (8.2) | 0.10 | 894 (7.8) | 914 (8.0) | | 0.007 |
| ARBs | 21347 (58.4) | 7239 (62.5) | 0.08 | 7122 (62.1) | 7149 (62.3) | | 0.005 |
| Entresto | 6 (0.0) | 17 (0.2) | 0.05 | 6 (0.1) | 9 (0.1) | | 0.010 |
| Other  Anti HTNs | 23952 (65.5) | 8062 (69.6) | 0.09 | 7977 (69.6) | 7952 (69.3) | | -0.005 |
| Loop diuretics | 4263 (11.7) | 1353 (11.7) | 0.00 | 1293 (11.3) | 1316 (11.5) | | 0.006 |
| Other diuretics | 9953 (27.2) | 3198 (27.6) | 0.01 | 3153 (27.5) | 3149 (27.5) | | -0.001 |
| Antianxieties | 14845 (40.6) | 4181 (36.1) | -0.09 | 4153 (36.2) | 4151 (36.2) | | 0.000 |
| Antipsychotics | 1776 (4.9) | 297 (2.6) | -0.12 | 298 (2.6) | 296 (2.6) | | -0.001 |
| Antidepressants | 6589 (18.0) | 1748 (15.1) | -0.08 | 1694 (14.8) | 1737 (15.2) | | 0.011 |
| Dementia | 5932 (16.2) | 1144 (9.9) | -0.19 | 1130 (9.9) | 1144 (10.0) | | 0.004 |
| Antiparkinsons | 1121 (3.1) | 178 (1.5) | -0.10 | 179 (1.6) | 178 (1.6) | | -0.001 |
| Anticonvulsants | 921 (2.5) | 182 (1.6) | -0.07 | 189 (1.7) | 182 (1.6) | | -0.005 |
| NSAIDs | 27797 (76.1) | 8731 (75.4) | -0.02 | 8660 (75.5) | 8649 (75.4) | | -0.002 |
| Bisphospho-nates | 1752 (4.8) | 368 (3.2) | -0.08 | 385 (3.4) | 368 (3.2) | | -0.008 |
| Opioids | 16234 (44.4) | 4735 (40.9) | -0.07 | 4669 (40.7) | 4695 (40.9) | | 0.005 |

Values are represented as mean ± standard deviation or number (%); ACEis, angiotensin-converting enzyme inhibitors; AGIs, α-glucosidase Inhibitors; ARBs, angiotensin II receptor blockers; CABG, coronary artery bypass graft; CAD, coronary artery disease; COPD, chronic obstructive pulmonary disease; CV, cardiovascular; DM, diabetes mellitus; HONK, hyperglycaemic hyperosmolar nonketotic coma; HTN, hypertensive; MI, myocardial infarction; NSAIDs, non-steroidal anti-inflammatory drugs; PAD, peripheral artery disease; PUD, peptic ulcer disease; STD, standardized difference; SUs, sulfonylureas; UC, ulcerative colitis;

**Supplementary Table S12 Baseline characteristics of cohort for thromboembolic event**

|  | **Pre-Match** | | **STD** | **Post-Match** | | | **STD** |
| --- | --- | --- | --- | --- | --- | --- | --- |
|  | **Sitagliptin**  **N= 34,363** | **Empagliflozin**  **N= 10,940** |  | **Sitagliptin**  **N= 10,808** | **Empagliflozin**  **N= 10,808** | |  |
| Sex | 19092 (55.6) | 6510 (59.5) | -0.04 | 6418 (59.4) | 6414 (59.3) | | 0.001 |
| Age | 60.1 ± 11.4 | 55.2 ± 10.9 | -0.44 | 55.3 ± 11.2 | 55.4 ± 10.8 | | 0.009 |
| Insurance type | | | | | | | |
| Normal | 32172 (93.6) | 10388 (95.0) | 0.06 | 10220 (94.6) | | 10259 (94.9) | 0.016 |
| Medicaid | 2021 (5.9) | 521 (4.8) |  | 555 (5.1) | 518 (4.8) | |  |
| No charge | 170 (0.5) | 31 (0.3) |  | 33 (0.3) | 31 (0.3) | |  |
| Number of  Inpatient visit | 0.8 ± 2.2 | 0.4 ± 1.2 | -0.20 | 0.5 ± 1.1 | 0.4 ± 1.2 | | -0.009 |
| Number of  outpatient visit | 27.6 ± 27.2 | 25.1 ± 23.7 | -0.10 | 25.0 ± 23.6 | 25.1 ± 23.6 | | 0.005 |
| Index year | | | | | | | |
| 2016 | 9966 (29.0) | 2111 (19.3) | 0.23 | 2119 (19.6) | 2107 (19.5) | | 0.004 |
| 2017 | 17022 (49.5) | 5944 (54.3) |  | 5854 (54.2) | 5874 (54.4) | |  |
| 2018 | 7375 (21.5) | 2885 (26.4) |  | 2835 (26.2) | 2827 (26.2) | |  |
| Charlson comorbidity index | | | | | | | |
| 0 | 1477 (4.3) | 459 (4.2) | 0.03 | 439 (4.1) | 455 (4.2) | | 0.009 |
| 1 | 3220 (9.4) | 1109 (10.1) |  | 1082 (10.0) | 1097 (10.2) | |  |
| 2 | 4701 (13.7) | 1558 (14.2) |  | 1548 (14.3) | 1536 (14.2) | |  |
| 3 | 24965 (72.7) | 7814 (71.4) |  | 7739 (71.6) | 7720 (71.4) | |  |
| CV risk factor | | | | | | | |
| CAD | 30375 (88.4) | 10143 (92.7) | 0.15 | 9998 (92.5) | 10015 (92.7) | | 0.006 |
| Multi vessel CAD | 15081 (43.9) | 5789 (52.9) | 0.18 | 5569 (51.5) | 5677 (52.5) | | 0.020 |
| MI | 1775 (5.2) | 865 (7.9) | 0.11 | 795 (7.4) | 824 (7.6) | | 0.010 |
| CABG | 7085 (20.6) | 3123 (28.6) | 0.18 | 3045 (28.2) | 3042 (28.2) | | -0.001 |
| Stroke | 4830 (14.1) | 950 (8.7) | -0.17 | 940 (8.7) | 946 (8.8) | | 0.002 |
| PAD | 2134 (6.2) | 596 (5.5) | -0.03 | 611 (5.7) | 592 (5.5) | | -0.008 |
| DM circulation | 4534 (13.2) | 1661 (15.2) | 0.06 | 1606 (14.9) | 1629 (15.1) | | 0.006 |
| DM foot | 3 (0.0) | 4 (0.0) | 0.02 | 2 (0.0) | 1 (0.0) | | -0.008 |
| DM nephropathy | 2187 (6.4) | 949 (8.7) | 0.09 | 910 (8.4) | 919 (8.5) | | 0.003 |
| DM neuropathy | 4803 (14.0) | 1452 (13.3) | -0.02 | 1467 (13.6) | 1436 (13.3) | | -0.008 |
| DM other  Complications | 25412 (74.0) | 7806 (71.4) | -0.06 | 7771 (71.9) | 7727 (71.5) | | -0.009 |
| Hyperglycemia | 616 (1.8) | 142 (1.3) | -0.04 | 138 (1.3) | 139 (1.3) | | 0.001 |
| Comorbidities | | | | | | | |
| Hypertension | 26802 (78.0) | 8582 (78.5) | 0.01 | 8465 (78.3) | 8475 (78.4) | | 0.002 |
| Edema | 3118 (9.1) | 942 (8.6) | -0.02 | 912 (8.4) | 932 (8.6) | | 0.007 |
| Kidney stone | 538 (1.6) | 156 (1.4) | -0.01 | 155 (1.4) | 155 (1.4) | | 0.000 |
| Osteoarthritis | 12005 (34.9) | 3268 (29.9) | -0.11 | 3275 (30.3) | 3252 (30.1) | | -0.005 |
| Other arthritis | 8279 (24.1) | 2319 (21.2) | -0.07 | 2323 (21.5) | 2294 (21.2) | | -0.007 |
| PUD | 8626 (25.1) | 2583 (23.6) | -0.03 | 2554 (23.6) | 2557 (23.7) | | 0.001 |
| Pancreatitis | 324 (0.9) | 97 (0.9) | -0.01 | 86 (0.8) | 95 (0.9) | | 0.009 |
| UC | 49 (0.1) | 12 (0.1) | -0.01 | 8 (0.1) | 12 (0.1) | | 0.012 |
| Crohn | 12 (0.0) | 5 (0.1) | 0.01 | 5 (0.1) | 4 (0.0) | | -0.005 |
| Asthma | 4939 (14.4) | 1495 (13.7) | -0.02 | 1469 (13.6) | 1478 (13.7) | | 0.002 |
| COPD | 1197 (3.5) | 257 (2.4) | -0.07 | 268 (2.5) | 255 (2.4) | | -0.008 |
| Bladder stone | 28 (0.1) | 5 (0.1) | -0.01 | 4 (0.0) | 5 (0.1) | | 0.005 |
| Dementia | 5424 (15.8) | 1055 (9.6) | -0.19 | 1025 (9.5) | 1050 (9.7) | | 0.008 |
| Electrolyte  Imbalance | 2092 (6.1) | 529 (4.8) | -0.06 | 534 (4.9) | 521 (4.8) | | -0.006 |
| Glaucoma  /Cataract | 9553 (27.8) | 2899 (26.5) | -0.03 | 2873 (26.6) | 2869 (26.6) | | -0.001 |
| HONK | 262 (0.8) | 59 (0.5) | -0.03 | 54 (0.5) | 58 (0.5) | | 0.005 |
| HTN  nephropathy | 150 (0.4) | 50 (0.5) | 0.00 | 62 (0.6) | 50 (0.5) | | -0.016 |
| Hyperthyroid  disease | 649 (1.9) | 202 (1.9) | 0.00 | 192 (1.8) | 200 (1.9) | | 0.006 |
| Hypothyroid  disease | 1663 (4.8) | 554 (5.1) | 0.01 | 535 (5.0) | 547 (5.1) | | 0.005 |
| Osteomyelitis | 256 (0.7) | 60 (0.6) | -0.02 | 76 (0.7) | 60 (0.6) | | -0.019 |
| Pneumonia | 2597 (7.6) | 712 (6.5) | -0.04 | 698 (6.5) | 700 (6.5) | | 0.001 |
| Skin infection | 1329 (3.9) | 419 (3.8) | 0.00 | 412 (3.8) | 414 (3.8) | | 0.001 |
| Glucose-lowering therapy | | | | | | | |
| Metformin | 24059 (70.0) | 7941 (72.6) | 0.06 | 7830 (72.5) | 7844 (72.6) | | 0.003 |
| Insulins | 5724 (16.7) | 1919 (17.5) | 0.02 | 1874 (17.3) | 1876 (17.4) | | 0.001 |
| SUs | 15687 (45.7) | 5119 (46.8) | 0.02 | 5028 (46.5) | 5050 (46.7) | | 0.004 |
| Glitazones | 3053 (8.9) | 1236 (11.3) | 0.08 | 1218 (11.3) | 1202 (11.1) | | -0.005 |
| GLP-1 agonists | 98 (0.3) | 77 (0.7) | 0.06 | 63 (0.6) | 72 (0.7) | | 0.011 |
| AGIs | 1403 (4.1) | 334 (3.1) | -0.06 | 336 (3.1) | 333 (3.1) | | -0.002 |
| Meglitinides | 225 (0.7) | 79 (0.7) | 0.01 | 80 (0.7) | 79 (0.7) | | -0.001 |
| Co-medications | | | | | | | |
| Anticoagulants | 1365 (4.0) | 468 (4.3) | 0.02 | 428 (4.0) | 454 (4.2) | | 0.012 |
| Antiplatelets | 22686 (66.0) | 7698 (70.4) | 0.09 | 7575 (70.1) | 7592 (70.2) | | 0.003 |
| Heparins | 1092 (3.2) | 275 (2.5) | -0.04 | 281 (2.6) | 272 (2.5) | | -0.005 |
| Thrombolytics | 49 (0.1) | 9 (0.1) | -0.02 | 10 (0.1) | 9 (0.1) | | -0.003 |
| Statins | 24161 (70.3) | 8852 (80.9) | 0.25 | 8752 (81.0) | 8724 (80.7) | | -0.007 |
| Other lipid  Lowerings | 3672 (10.7) | 1583 (14.5) | 0.11 | 1551 (14.4) | 1543 (14.3) | | -0.002 |
| Nitrates | 5794 (16.9) | 2268 (20.7) | 0.10 | 2241 (20.7) | 2217 (20.5) | | -0.006 |
| Digoxin | 4985 (14.5) | 1974 (18.0) | 0.10 | 1948 (18.0) | 1924 (17.8) | | -0.006 |
| ACEIs | 1994 (5.8) | 900 (8.2) | 0.10 | 875 (8.1) | 860 (8.0) | | -0.005 |
| ARBs | 19956 (58.1) | 6802 (62.2) | 0.08 | 6698 (62.0) | 6708 (62.1) | | 0.002 |
| Entresto | 6 (0.0) | 15 (0.1) | 0.04 | 6 (0.1) | 9 (0.1) | | 0.011 |
| Other  Anti HTNs | 22385 (65.1) | 7586 (69.3) | 0.09 | 7509 (69.5) | 7475 (69.2) | | -0.007 |
| Loop diuretics | 3803 (11.1) | 1213 (11.1) | 0.00 | 1146 (10.6) | 1178 (10.9) | | 0.010 |
| Other diuretics | 9198 (26.8) | 2956 (27.0) | 0.01 | 2867 (26.5) | 2898 (26.8) | | 0.007 |
| Antianxieties | 13759 (40.0) | 3881 (35.5) | -0.09 | 3834 (35.5) | 3851 (35.6) | | 0.003 |
| Antipsychotics | 1602 (4.7) | 272 (2.5) | -0.12 | 260 (2.4) | 271 (2.5) | | 0.007 |
| Antidepressants | 6019 (17.5) | 1606 (14.7) | -0.08 | 1581 (14.6) | 1598 (14.8) | | 0.004 |
| Dementia | 5424 (15.8) | 1055 (9.6) | -0.19 | 1025 (9.5) | 1050 (9.7) | | 0.008 |
| Antiparkinsons | 1009 (2.9) | 156 (1.4) | -0.10 | 174 (1.6) | 156 (1.4) | | -0.014 |
| Anticonvulsants | 853 (2.5) | 166 (1.5) | -0.07 | 173 (1.6) | 166 (1.5) | | -0.005 |
| NSAIDs | 26008 (75.7) | 8215 (75.1) | -0.01 | 8119 (75.1) | 8124 (75.2) | | 0.001 |
| Bisphospho-nates | 1573 (4.6) | 328 (3.0) | -0.08 | 341 (3.2) | 327 (3.0) | | -0.008 |
| Opioids | 15076 (43.9) | 4380 (40.0) | -0.08 | 4398 (40.7) | 4335 (40.1) | | -0.012 |

Values are represented as mean ± standard deviation or number (%); ACEis, angiotensin-converting enzyme inhibitors; AGIs, α-glucosidase Inhibitors; ARBs, angiotensin II receptor blockers; CABG, coronary artery bypass graft; CAD, coronary artery disease; COPD, chronic obstructive pulmonary disease; CV, cardiovascular; DM, diabetes mellitus; HONK, hyperglycaemic hyperosmolar nonketotic coma; HTN, hypertensive; MI, myocardial infarction; NSAIDs, non-steroidal anti-inflammatory drugs; PAD, peripheral artery disease; PUD, peptic ulcer disease; STD, standardized difference; SUs, sulfonylureas; UC, ulcerative colitis;

**Supplementary Table S13 Baseline characteristics of cohort for fracture**

|  | **Pre-Match** | | **STD** | **Post-Match** | | | **STD** |
| --- | --- | --- | --- | --- | --- | --- | --- |
|  | **Sitagliptin**  **N= 28,301** | **Empagliflozin**  **N= 9,467** |  | **Sitagliptin**  **N= 9,360** | **Empagliflozin**  **N= 9,360** | |  |
| Sex | 16621 (58.7) | 5866 (62.0) | -0.03 | 5770 (61.7) | 5782 (61.8) | | -0.007 |
| Age | 59.5 ± 11.3 | 54.9 ± 10.9 | -0.42 | 55.0 ± 11.2 | 55.1 ± 10.7 | | 0.007 |
| Insurance type | | | | | | | |
| Normal | 26627 (94.1) | 9026 (95.3) | 0.06 | 8954 (95.7) | | 8925 (95.4) | 0.016 |
| Medicaid | 1515 (5.4) | 413 (4.4) |  | 382 (4.1) | 407 (4.4) | |  |
| No charge | 159 (0.6) | 28 (0.3) |  | 24 (0.3) | 28 (0.3) | |  |
| Number of  Inpatient visit | 0.7 ± 2.0 | 0.4 ± 1.1 | -0.17 | 0.4 ± 1.1 | 0.4 ± 1.1 | | 0.005 |
| Number of  outpatient visit | 26.2 ± 25.3 | 24.2 ± 22.9 | -0.08 | 23.9 ± 21.8 | 24.3 ± 23.0 | | 0.014 |
| Index year | | | | | | | |
| 2016 | 8459 (29.9) | 1860 (19.7) | 0.25 | 1878 (20.1) | 1859 (19.9) | | 0.006 |
| 2017 | 13972 (49.4) | 5105 (53.9) |  | 5046 (53.9) | 5046 (53.9) | |  |
| 2018 | 5870 (20.7) | 2502 (26.4) |  | 2436 (26.0) | 2455 (26.2) | |  |
| Charlson comorbidity index | | | | | | | |
| 0 | 1302 (4.6) | 430 (4.5) | 0.02 | 444 (4.7) | 422 (4.5) | | 0.015 |
| 1 | 2771 (9.8) | 974 (10.3) |  | 936 (10.0) | 967 (10.3) | |  |
| 2 | 4003 (14.1) | 1357 (14.3) |  | 1355 (14.5) | 1353 (14.5) | |  |
| 3 | 20225 (71.5) | 6706 (70.8) |  | 6625 (70.8) | 6618 (70.7) | |  |
| CV risk factor | | | | | | | |
| CAD | 25173 (89.0) | 8780 (92.7) | 0.13 | 8662 (92.5) | 8677 (92.7) | | 0.006 |
| Multi vessel CAD | 12600 (44.5) | 4996 (52.8) | 0.17 | 4849 (51.8) | 4911 (52.5) | | 0.013 |
| MI | 1520 (5.4) | 764 (8.1) | 0.11 | 713 (7.6) | 731 (7.8) | | 0.007 |
| CABG | 6024 (21.3) | 2767 (29.2) | 0.18 | 2648 (28.3) | 2701 (28.9) | | 0.013 |
| Stroke | 3730 (13.2) | 817 (8.6) | -0.15 | 818 (8.7) | 809 (8.6) | | -0.003 |
| PAD | 1777 (6.3) | 527 (5.6) | -0.03 | 522 (5.6) | 524 (5.6) | | 0.001 |
| DM circulation | 3674 (13.0) | 1426 (15.1) | 0.06 | 1414 (15.1) | 1410 (15.1) | | -0.001 |
| DM foot | 3 (0.0) | 3 (0.0) | 0.01 | 1 (0.0) | 3 (0.0) | | 0.015 |
| DM nephropathy | 1816 (6.4) | 820 (8.7) | 0.09 | 782 (8.4) | 794 (8.5) | | 0.005 |
| DM neuropathy | 3828 (13.5) | 1232 (13.0) | -0.02 | 1221 (13.0) | 1220 (13.0) | | 0.000 |
| DM other  Complications | 20682 (73.1) | 6696 (70.7) | -0.05 | 6664 (71.2) | 6628 (70.8) | | -0.009 |
| Hyperglycemia | 488 (1.7) | 118 (1.3) | -0.04 | 110 (1.2) | 117 (1.3) | | 0.007 |
| Comorbidities | | | | | | | |
| Hypertension | 22064 (78.0) | 7428 (78.5) | 0.01 | 7368 (78.7) | 7343 (78.5) | | -0.007 |
| Edema | 2502 (8.8) | 801 (8.5) | -0.01 | 744 (8.0) | 790 (8.4) | | 0.018 |
| Kidney stone | 446 (1.6) | 130 (1.4) | -0.02 | 116 (1.2) | 129 (1.4) | | 0.012 |
| Osteoarthritis | 9217 (32.6) | 2680 (28.3) | -0.09 | 2661 (28.4) | 2670 (28.5) | | 0.002 |
| Other arthritis | 6515 (23.0) | 1917 (20.3) | -0.07 | 1870 (20.0) | 1903 (20.3) | | 0.009 |
| PUD | 6838 (24.2) | 2212 (23.4) | -0.02 | 2188 (23.4) | 2198 (23.5) | | 0.003 |
| Pancreatitis | 240 (0.9) | 75 (0.8) | -0.01 | 77 (0.8) | 75 (0.8) | | -0.002 |
| UC | 46 (0.2) | 12 (0.1) | -0.01 | 11 (0.1) | 12 (0.1) | | 0.003 |
| Crohn | 12 (0.0) | 3 (0.0) | -0.01 | 4 (0.0) | 3 (0.0) | | -0.006 |
| Asthma | 3933 (13.9) | 1256 (13.3) | -0.02 | 1244 (13.3) | 1246 (13.3) | | 0.001 |
| COPD | 935 (3.3) | 211 (2.2) | -0.07 | 224 (2.4) | 211 (2.3) | | -0.009 |
| Bladder stone | 23 (0.1) | 3 (0.0) | -0.02 | 6 (0.1) | 3 (0.0) | | -0.015 |
| Dementia | 4055 (14.3) | 864 (9.1) | -0.16 | 828 (8.9) | 861 (9.2) | | 0.012 |
| Electrolyte  Imbalance | 1615 (5.7) | 481 (5.1) | -0.03 | 469 (5.0) | 472 (5.0) | | 0.002 |
| Glaucoma  /Cataract | 7812 (27.6) | 2493 (26.3) | -0.03 | 2434 (26.0) | 2476 (26.5) | | 0.010 |
| HONK | 205 (0.7) | 50 (0.5) | -0.02 | 46 (0.5) | 50 (0.5) | | 0.006 |
| HTN  nephropathy | 131 (0.5) | 43 (0.5) | 0.00 | 44 (0.5) | 41 (0.4) | | -0.005 |
| Hyperthyroid  disease | 530 (1.9) | 181 (1.9) | 0.00 | 164 (1.8) | 180 (1.9) | | 0.013 |
| Hypothyroid  disease | 1325 (4.7) | 470 (5.0) | 0.01 | 457 (4.9) | 459 (4.9) | | 0.001 |
| Osteomyelitis | 192 (0.7) | 50 (0.5) | -0.02 | 57 (0.6) | 50 (0.5) | | -0.010 |
| Pneumonia | 2053 (7.3) | 605 (6.4) | -0.03 | 608 (6.5) | 597 (6.4) | | -0.005 |
| Skin infection | 1042 (3.7) | 353 (3.7) | 0.00 | 374 (4.0) | 350 (3.7) | | -0.013 |
| Glucose-lowering therapy | | | | | | | |
| Metformin | 19907 (70.3) | 6824 (72.1) | 0.04 | 6724 (71.8) | 6757 (72.2) | | 0.008 |
| Insulins | 4420 (15.6) | 1629 (17.2) | 0.04 | 1549 (16.6) | 1584 (16.9) | | 0.010 |
| SUs | 12905 (45.6) | 4358 (46.0) | 0.01 | 4298 (45.9) | 4302 (46.0) | | 0.001 |
| Glitazones | 2484 (8.8) | 1047 (11.1) | 0.08 | 1034 (11.1) | 1028 (11.0) | | -0.002 |
| GLP-1 agonists | 88 (0.3) | 70 (0.7) | 0.06 | 62 (0.7) | 60 (0.6) | | -0.003 |
| AGIs | 1138 (4.0) | 286 (3.0) | -0.05 | 284 (3.0) | 285 (3.0) | | 0.001 |
| Meglitinides | 184 (0.7) | 72 (0.8) | 0.01 | 69 (0.7) | 68 (0.7) | | -0.001 |
| Co-medications | | | | | | | |
| Anticoagulants | 1226 (4.3) | 444 (4.7) | 0.02 | 410 (4.4) | 433 (4.6) | | 0.012 |
| Antiplatelets | 18893 (66.8) | 6675 (70.5) | 0.08 | 6553 (70.0) | 6592 (70.4) | | 0.009 |
| Heparins | 982 (3.5) | 282 (3.0) | -0.03 | 297 (3.2) | 280 (3.0) | | -0.011 |
| Thrombolytics | 40 (0.1) | 9 (0.1) | -0.01 | 4 (0.0) | 9 (0.1) | | 0.020 |
| Statins | 20036 (70.8) | 7687 (81.2) | 0.25 | 7576 (80.9) | 7583 (81.0) | | 0.002 |
| Other lipid  Lowerings | 3119 (11.0) | 1383 (14.6) | 0.11 | 1381 (14.8) | 1340 (14.3) | | -0.012 |
| Nitrates | 4868 (17.2) | 1981 (20.9) | 0.09 | 1946 (20.8) | 1940 (20.7) | | -0.002 |
| Digoxin | 4187 (14.8) | 1724 (18.2) | 0.09 | 1698 (18.1) | 1684 (18.0) | | -0.004 |
| ACEIs | 1697 (6.0) | 793 (8.4) | 0.09 | 752 (8.0) | 758 (8.1) | | 0.002 |
| ARBs | 16568 (58.5) | 5903 (62.4) | 0.08 | 5890 (62.9) | 5829 (62.3) | | -0.014 |
| Entresto | 6 (0.0) | 14 (0.2) | 0.04 | 5 (0.1) | 8 (0.1) | | 0.012 |
| Other  Anti HTNs | 18460 (65.2) | 6577 (69.5) | 0.09 | 6457 (69.0) | 6482 (69.3) | | 0.006 |
| Loop diuretics | 3025 (10.7) | 1050 (11.1) | 0.01 | 995 (10.6) | 1018 (10.9) | | 0.008 |
| Other diuretics | 7554 (26.7) | 2584 (27.3) | 0.01 | 2561 (27.4) | 2539 (27.1) | | -0.005 |
| Antianxieties | 10948 (38.7) | 3259 (34.4) | -0.09 | 3214 (34.3) | 3238 (34.6) | | 0.005 |
| Antipsychotics | 1138 (4.0) | 222 (2.3) | -0.10 | 218 (2.3) | 222 (2.4) | | 0.003 |
| Antidepressants | 4609 (16.3) | 1298 (13.7) | -0.07 | 1279 (13.7) | 1291 (13.8) | | 0.004 |
| Dementia | 4055 (14.3) | 864 (9.1) | -0.16 | 828 (8.9) | 861 (9.2) | | 0.012 |
| Antiparkinsons | 767 (2.7) | 129 (1.4) | -0.10 | 125 (1.3) | 129 (1.4) | | 0.004 |
| Anticonvulsants | 643 (2.3) | 137 (1.5) | -0.06 | 138 (1.5) | 137 (1.5) | | -0.001 |
| NSAIDs | 20959 (74.1) | 6957 (73.5) | -0.01 | 6933 (74.1) | 6886 (73.6) | | -0.011 |
| Bisphospho-nates | 914 (3.2) | 219 (2.3) | -0.06 | 218 (2.3) | 219 (2.3) | | 0.001 |
| Opioids | 11831 (41.8) | 3629 (38.3) | -0.07 | 3568 (38.1) | 3596 (38.4) | | 0.006 |

Values are represented as mean ± standard deviation or number (%); ACEis, angiotensin-converting enzyme inhibitors; AGIs, α-glucosidase Inhibitors; ARBs, angiotensin II receptor blockers; CABG, coronary artery bypass graft; CAD, coronary artery disease; COPD, chronic obstructive pulmonary disease; CV, cardiovascular; DM, diabetes mellitus; HONK, hyperglycaemic hyperosmolar nonketotic coma; HTN, hypertensive; MI, myocardial infarction; NSAIDs, non-steroidal anti-inflammatory drugs; PAD, peripheral artery disease; PUD, peptic ulcer disease; STD, standardized difference; SUs, sulfonylureas; UC, ulcerative colitis;

**Supplementary Table S14 Sensitivity analysis for cardiovascular outcomes after follow-up of patients who received at least 1 dose of study drug were observed until ≤30 days after a patient’s last intake of medication.**

| **Outcomes** | **EMPA-REG Outcome® (RCT)** | | **EMPA-REG Duplicate (RWE)** | | **STD** | **Agreement** | | |
| --- | --- | --- | --- | --- | --- | --- | --- | --- |
|  | **Rate/1000**  **Patient-yr** | **HR**  **(95% CI)** | **Rate/1000**  **Patient-yr** | **HR**  **(95% CI)** |  | **RA** | **EA** | **SD** |
| ***MACEs*** | | | | | | | | |
| Sitagliptin | 43.9 | 0.86  (0.74-0.99) | 20.6 | 0.88  (0.77-0.99) | 0.2 | Y | Y | Y |
| Empagliflozin | 37.4 |  | 18.1 |  |  |  |  |  |
| ***All-cause death*** | | | | | | | | |
| Sitagliptin | 28.6 | 0.68  (0.57-0.82) | 6.2 | 0.84  (0.66-1.07) | 1.3 | N | N | Y |
| Empagliflozin | 19.4 |  | 5.1 |  |  |  |  |  |
| ***Myocardial infarction*** | | | | | | | | |
| Sitagliptin | 19.3 | 0.87  (0.70-1.09) | 12.2 | 0.84  (0.71-0.99) | -0.2 | N | Y | Y |
| Empagliflozin | 16.8 |  | 10.3 |  |  |  |  |  |
| ***Stroke*** | | | | | | | | |
| Sitagliptin | 10.5 | 1.18  (0.89-1.56) | 11.5 | 0.85  (0.72-1.01) | -2.0 | N | N | N |
| Empagliflozin | 12.3 |  | 9.8 |  |  |  |  |  |
| ***Hospitalization for unstable angina*** | | | | | | | | |
| Sitagliptin | 10.0 | 0.99  (0.74-1.34) | 30.8 | 0.91  (0.82-1.01) | -0.5 | Y | Y | Y |
| Empagliflozin | 10.0 |  | 28.3 |  |  |  |  |  |
| ***Coronary revascularization*** | | | | | | | | |
| Sitagliptin | 29.1 | 0.86  (0.72-1.04) | 33.6 | 0.98  (0.89-1.07) | 1.2 | Y | Y | Y |
| Empagliflozin | 25.1 |  | 33.4 |  |  |  |  |  |
| ***Transient ischemic attack*** | | | | | | | | |
| Sitagliptin | 3.5 | 0.85  (0.51-1.42) | 8.6 | 0.83  (0.67-1.01) | -0.1 | Y | Y | Y |
| Empagliflozin | 2.9 |  | 7.1 |  |  |  |  |  |
| ***Hospitalization for heart failure*** | | | | | | | | |
| Sitagliptin | 14.5 | 0.65  (0.50-0.85) | 18.4 | 0.82  (0.71-0.95) | 1.5 | Y | Y | Y |
| Empagliflozin | 9.4 |  | 15.1 |  |  |  |  |  |

EA, estimate agreement; HR, hazard ratio; CI, confidence interval; MACEs, major adverse cardiovascular events; RA, regulatory agreement, RCT, randomized clinical trial; RWE, real-world evidence; SD, standardized difference; STD, standardized difference; Y, yes; N, no;

**Supplementary Table S15 Sensitivity analysis for** **cardiovascular outcomes after follow-up of patients who received study drug for ≥30 days (cumulative) including only events that occurred ≤30 days after a patient’s last intake of medication**

| **Outcomes** | **EMPA-REG Outcome® (RCT)** | | **EMPA-REG Duplicate (RWE)** | | **STD** | **Agreement** | | |
| --- | --- | --- | --- | --- | --- | --- | --- | --- |
|  | **Rate/1000**  **Patient-yr** | **HR**  **(95% CI)** | **Rate/1000**  **Patient-yr** | **HR**  **(95% CI)** |  | **RA** | **EA** | **SD** |
| ***MACEs*** | | | | | | | | |
| Sitagliptin | 43.9 | 0.86  (0.74-0.99) | 25.5 | 0.87  (0.79-0.96) | 0.1 | Y | Y | Y |
| Empagliflozin | 37.4 |  | 22.5 |  |  |  |  |  |
| ***All-cause death*** | | | | | | | | |
| Sitagliptin | 28.6 | 0.68  (0.57-0.82) | 9.7 | 0.87  (0.74-1.03) | 1.7 | N | N | Y |
| Empagliflozin | 19.4 |  | 8.7 |  |  |  |  |  |
| ***Myocardial infarction*** | | | | | | | | |
| Sitagliptin | 19.3 | 0.87  (0.70-1.09) | 13.3 | 0.89  (0.77-1.02) | 0.2 | Y | Y | Y |
| Empagliflozin | 16.8 |  | 12.0 |  |  |  |  |  |
| ***Stroke*** | | | | | | | | |
| Sitagliptin | 10.5 | 1.18  (0.89-1.56) | 13.0 | 0.85  (0.74-0.98) | -2.1 | N | N | N |
| Empagliflozin | 12.3 |  | 11.3 |  |  |  |  |  |
| ***Hospitalization for unstable angina*** | | | | | | | | |
| Sitagliptin | 10.0 | 0.99  (0.74-1.34) | 29.4 | 0.97  (0.88-1.07) | -0.1 | Y | Y | Y |
| Empagliflozin | 10.0 |  | 28.9 |  |  |  |  |  |
| ***Coronary revascularization*** | | | | | | | | |
| Sitagliptin | 29.1 | 0.86  (0.72-1.04) | 33.9 | 1.01  (0.93-1.10) | 1.5 | N | Y | Y |
| Empagliflozin | 25.1 |  | 34.6 |  |  |  |  |  |
| ***Transient ischemic attack*** | | | | | | | | |
| Sitagliptin | 3.5 | 0.85  (0.51-1.42) | 9.1 | 0.86  (0.72-1.02) | 0.0 | Y | Y | Y |
| Empagliflozin | 2.9 |  | 7.9 |  |  |  |  |  |
| ***Hospitalization for heart failure*** | | | | | | | | |
| Sitagliptin | 14.5 | 0.65  (0.50-0.85) | 19.6 | 0.87  (0.77-0.98) | 1.9 | Y | N | Y |
| Empagliflozin | 9.4 |  | 17.1 |  |  |  |  |  |

EA, estimate agreement; HR, hazard ratio; CI, confidence interval; MACEs, major adverse cardiovascular events; RA, regulatory agreement, RCT, randomized clinical trial; RWE, real-world evidence; SD, standardized difference; STD, standardized difference; Y, yes; N, no;

**Supplementary Table S16 Sensitivity analysis for safety outcomes after follow-up of patients who received at least 1 dose of study drug were observed until ≤30 days after a patient’s last intake of medication.**

| **Outcomes** | **EMPA-REG Outcome® (RCT)** | | **EMPA-REG Duplicate**  **(RWE)** | | **STD** | **Agreement** | | |
| --- | --- | --- | --- | --- | --- | --- | --- | --- |
|  | **Rate (%)** | **OR**  **(95% CI)** | **Rate (%)** | **OR**  **(95% CI)** |  | **RA** | **EA** | **SD** |
| ***Hypoglycemic adverse event*** | | | | | | | | |
| Sitagliptin | 27.9 | 1.00  (0.89-1.11) | 1.6 | 0.76  (0.61-0.95) | -2.1 | N | N | N |
| Empagliflozin | 27.8 |  | 1.2 |  |  |  |  |  |
| ***Urinary tract infection*** | | | | | | | | |
| Sitagliptin | 18.1 | 0.99  (0.87-1.13) | 16.6 | 0.91  (0.84-0.99) | -1.0 | N | Y | Y |
| Empagliflozin | 18.0 |  | 15.4 |  |  |  |  |  |
| ***Genital infection*** | | | | | | | | |
| Sitagliptin | 1.8 | 3.74  (2.70-5.19) | 5.7 | 1.74  (1.55-1.95) | -4.3 | Y | N | N |
| Empagliflozin | 6.4 |  | 9.5 |  |  |  |  |  |
| ***Acute kidney injury*** | | | | | | | | |
| Sitagliptin | 6.6 | 0.78  (0.63-0.96) | 2.1 | 0.62  (0.50-0.76) | -1.6 | Y | N | Y |
| Empagliflozin | 5.2 |  | 1.3 |  |  |  |  |  |
| ***Volume depletion*** | | | | | | | | |
| Sitagliptin | 4.9 | 1.04  (0.82-1.30) | 4.9 | 0.86  (0.75-0.98) | -1.4 | N | Y | Y |
| Empagliflozin | 5.1 |  | 4.3 |  |  |  |  |  |
| ***Diabetic ketoacidosis*** | | | | | | | | |
| Sitagliptin | 0.04 | 1.99  (0.2-17.8) | 0.3 | 1.23  (0.76-2.00) | -0.4 | Y | Y | Y |
| Empagliflozin | 0.1 |  | 0.3 |  |  |  |  |  |
| ***Thromboembolic event*** | | | | | | | | |
| Sitagliptin | 0.9 | 0.75  (0.42-1.31) | 3.0 | 0.83  (0.70-0.97) | 0.3 | N | Y | Y |
| Empagliflozin | 0.6 |  | 2.5 |  |  |  |  |  |
| ***Fracture*** | | | | | | | | |
| Sitagliptin | 3.9 | 0.98  (0.76-1.27) | 9.7 | 0.94  (0.85-1.03) | -0.3 | Y | Y | Y |
| Empagliflozin | 3.8 |  | 9.1 |  |  |  |  |  |

EA, estimate agreement; HR, hazard ratio; CI, confidence interval; MACEs, major adverse cardiovascular events; RA, regulatory agreement, RCT, randomized clinical trial; RWE, real-world evidence; SD, standardized difference; STD, standardized difference; Y, yes; N, no;

**Supplementary Table S17 Sensitivity analysis for safety outcomes after follow-up of patients who received study drug for ≥30 days (cumulative) including only events that occurred ≤30 days after a patient’s last intake of medication**

| **Outcomes** | **EMPA-REG Outcome® (RCT)** | | **EMPA-REG Duplicate**  **(RWE)** | | **STD** | **Agreement** | | |
| --- | --- | --- | --- | --- | --- | --- | --- | --- |
|  | **Rate (%)** | **OR**  **(95% CI)** | **Rate (%)** | **OR**  **(95% CI)** |  | **RA** | **EA** | **SD** |
| ***Hypoglycemic adverse event*** | | | | | | | | |
| Sitagliptin | 27.9 | 1.00  (0.89-1.11) | 2.3 | 0.75  (0.62-0.90) | -2.6 | N | N | N |
| Empagliflozin | 27.8 |  | 1.7 |  |  |  |  |  |
| ***Urinary tract infection*** | | | | | | | | |
| Sitagliptin | 18.1 | 0.99  (0.87-1.13) | 21.2 | 0.94  (0.87-1.01) | -0.7 | Y | Y | Y |
| Empagliflozin | 18.0 |  | 20.2 |  |  |  |  |  |
| ***Genital infection*** | | | | | | | | |
| Sitagliptin | 1.8 | 3.74  (2.70-5.19)) | 7.2 | 1.62  (1.46-1.79) | -4.8 | Y | N | N |
| Empagliflozin | 6.4 |  | 11.2 |  |  |  |  |  |
| ***Acute kidney injury*** | | | | | | | | |
| Sitagliptin | 6.6 | 0.78  (0.63-0.96) | 2.8 | 0.74  (0.63-0.88) | -0.3 | Y | Y | Y |
| Empagliflozin | 5.2 |  | 2.1 |  |  |  |  |  |
| ***Volume depletion*** | | | | | | | | |
| Sitagliptin | 4.9 | 1.04  (0.82-1.30) | 6.5 | 0.89  (0.79-1.00) | -1.2 | N | Y | Y |
| Empagliflozin | 5.1 |  | 5.8 |  |  |  |  |  |
| ***Diabetic ketoacidosis*** | | | | | | | | |
| Sitagliptin | 0.04 | 1.99  (0.2-17.8) | 0.3 | 1.13  (0.73-1.74) | -0.5 | Y | Y | Y |
| Empagliflozin | 0.1 |  | 0.4 |  |  |  |  |  |
| ***Thromboembolic event*** | | | | | | | | |
| Sitagliptin | 0.9 | 0.75  (0.42-1.31) | 3.9 | 0.96  (0.84-1.11) | 0.9 | Y | Y | Y |
| Empagliflozin | 0.6 |  | 3.7 |  |  |  |  |  |
| ***Fracture*** | | | | | | | | |
| Sitagliptin | 3.9 | 0.98  (0.76-1.27) | 12.5 | 1.02  (0.93-1.11) | 0.3 | N | Y | Y |
| Empagliflozin | 3.8 |  | 12.7 |  |  |  |  |  |

EA, estimate agreement; HR, hazard ratio; CI, confidence interval; MACEs, major adverse cardiovascular events; RA, regulatory agreement, RCT, randomized clinical trial; RWE, real-world evidence; SD, standardized difference; STD, standardized difference; Y, yes; N, no;
